# High-Throughput Venomics

**DOI:** 10.1021/acs.jproteome.2c00780

**Published:** 2023-04-03

**Authors:** Julien Slagboom, Rico J. E. Derks, Raya Sadighi, Govert W. Somsen, Chris Ulens, Nicholas R. Casewell, Jeroen Kool

**Affiliations:** † Amsterdam Institute of Molecular and Life Sciences, Division of BioAnalytical Chemistry, Department of Chemistry and Pharmaceutical Sciences, Faculty of Science, Vrije Universiteit Amsterdam, De Boelelaan 1085, Amsterdam 1081HV, The Netherlands; ‡ Center for Proteomics and Metabolomics, Leiden Universitair Medisch Centrum, Albinusdreef 2, Leiden 2333 ZA, The Netherlands; § Laboratory of Structural Neurobiology, Department of Cellular and Molecular Medicine, Faculty of Medicine, KU Leuven, Leuven 3000, Belgium; ∥ Centre for Snakebite Research and Interventions, Liverpool School of Tropical Medicine, Pembroke Place, Liverpool L3 5QA, U.K.

**Keywords:** proteomics, venomics, mass spectrometry, high-throughput, venoms, fractionation, RP-HPLC, high-throughput proteomics

## Abstract

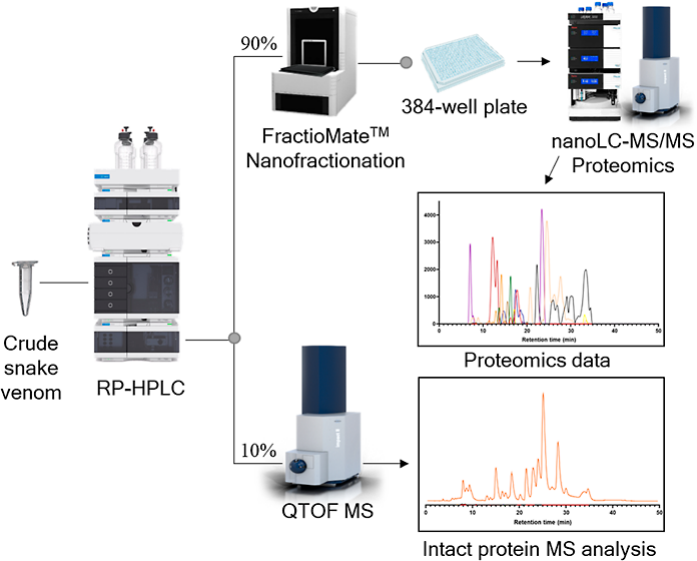

In this study, we present high-throughput (HT) venomics, a novel analytical strategy capable of performing a full proteomic analysis of a snake venom within 3 days. This methodology comprises a combination of RP-HPLC-nanofractionation analytics, mass spectrometry analysis, automated in-solution tryptic digestion, and high-throughput proteomics. In-house written scripts were developed to process all the obtained proteomics data by first compiling all Mascot search results for a single venom into a single Excel sheet. Then, a second script plots each of the identified toxins in so-called Protein Score Chromatograms (PSCs). For this, for each toxin, identified protein scores are plotted on the *y*-axis versus retention times of adjacent series of wells in which a toxin was fractionated on the *x*-axis. These PSCs allow correlation with parallel acquired intact toxin MS data. This same script integrates the PSC peaks from these chromatograms for semiquantitation purposes. This new HT venomics strategy was performed on venoms from diverse medically important biting species; *Calloselasma rhodostoma*, *Echis ocellatus*, *Naja pallida*, *Bothrops asper*, *Bungarus multicinctus*, *Crotalus atrox*, *Daboia russelii*, *Naja naja*, *Naja nigricollis*, *Naja mossambica*, and *Ophiophagus hannah*. Our data suggest that high-throughput venomics represents a valuable new analytical tool for increasing the throughput by which we can define venom variation and should greatly aid in the future development of new snakebite treatments by defining toxin composition.

## Introduction

Up to 5.4 million people suffer from snakebite annually, resulting in 2.7 million envenomings, 138,000 fatalities, and more than 400,000 cases of permanent disabilities.^1−4^ The greatest burden is suffered by agricultural workers and children from low-resource rural regions of the tropics, such as sub-Saharan Africa and South-east Asia.^2−4^ High incidence and fatality rates in these parts of the world are in part the consequences of the low socioeconomic status of those countries, which translates into restricted access to specialized medical care, which is, in turn, caused by limited logistical and health infrastructure.^1−5^ Antivenom is currently the only available therapy for the treatment of snakebite envenoming and comprises polyclonal antibody preparations derived from horses or sheep immunized with non-lethal doses of snake venom.^5,6^ The IgG antibodies in antivenoms, or fragments thereof, exhibit specificity toward venom toxins and can neutralize their activities and prevent severe pathology. When administration of antivenom occurs soon after envenoming, pathology symptoms can decline within a short period of time.^5,7^ Despite antivenoms being successfully used to treat hundreds of thousands of patients annually, there are several limitations associated with them.^6−9^ Major issues with current antivenoms available in the tropical world include limited paraspecific efficacy (limited to the snake species used for immunization), poor dose efficacy (10–20% of the generated antibodies are specific toward venom toxins), and a high chance of inducing severe side effects (up to 75% of reported incidences).^4−8,10^ However, for most victims, antivenom therapies are simply unaffordable since snakebites mainly affect the most impoverished regions of the world.^3,4^ The cost of a vial of antivenom can range over $50–350 in Africa, while treatment can require up to 20 vials, thereby making access to antivenom therapy for the majority of victims highly restrictive.^3,4,6,8^ Therefore, the development of specific, efficient, safe, and affordable next-generation antivenoms is urgently needed to tackle the devastating consequences of snakebite envenoming. To assist in the process of developing these next-generation antivenoms, a solid and ever-increasing knowledge basis on the in-depth composition of venom toxins in medically relevant snake venoms is required to identify the key similarities and distinctions between the variable pathogenic toxins observed across different snake species.

Snake venoms comprise many different proteins and peptides, which are primarily used for immobilizing and killing prey animals.^1,10,11^ However, snakes will also employ their venoms defensively as in the case of human snakebite. Resulting snake venom toxicities can be divided into three major classes, namely, those that cause hemotoxic, cytotoxic, and/or neurotoxic pathologies.^1,12,13^ Proteomics approaches have been used for many years to identify the variable toxins found across snake venoms as these can differ both inter- and intra-specifically.^14−17^ In 2004, the proteome of the dusky pigmy rattlesnake, *Sistrurus miliarius barbouri*, was unraveled and the name venomics was adopted.^18^ Venomics describes the use of an analytical strategy to identify the protein composition of snake venoms and has evolved “from low-resolution toxin-pattern recognition” to “toxin-resolved venom proteomes with absolute quantification” as described by Juarez et al.^18^ Despite great advancements made in the field of venomics, it remains laborious and time consuming due to the requirement for multidimensional separations (e.g., reversed phase liquid chromatography, RPLC, followed by gel electrophoresis), manual in-gel tryptic digestions, and long analysis run times, often combined with manual data processing.^14,16,17,19^

**Figure 1:**
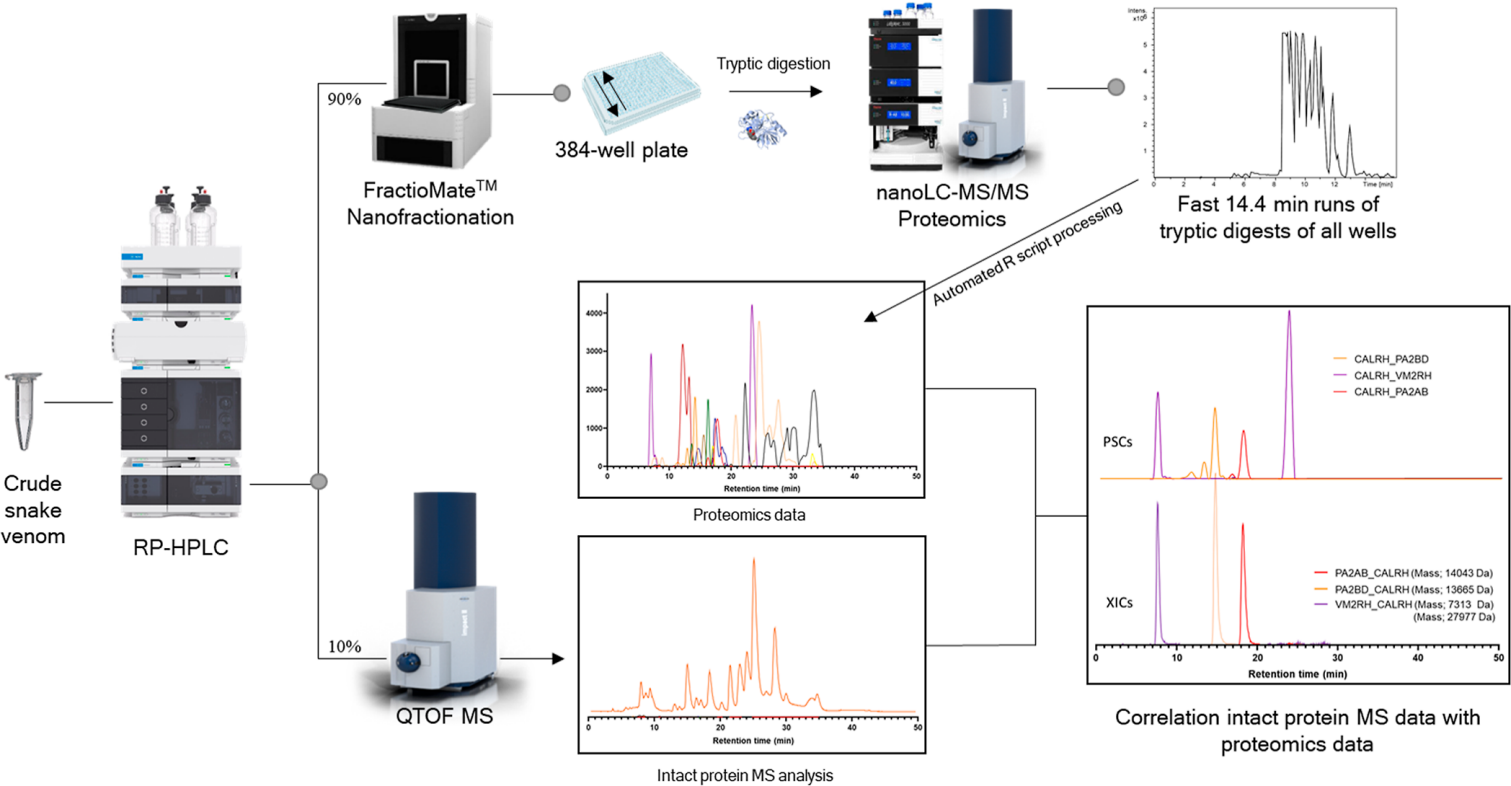
Graphical overview of the high-throughput venomics workflow. First, snake venom was subjected to nanofractionation analytics, which involves liquid chromatographic separation of venom toxins followed by a flow split of 10% to mass spectrometry (MS) for intact toxin analysis and 90% to parallel high-resolution fractionation of the separated venom toxins on to a 384-well plate. After vacuum-centrifugation of the well plate to evaporate the eluents, a tryptic digestion procedure is performed directly on the well plate using automated pipetting steps. The well plate is then directly transferred to nanoLC–MS/MS for analysis using a fast-analytical gradient runtime of 14.4 min, resulting in 100 measurements per day. The proteomics data obtained are then automatically subjected to Mascot database searching using Mascot Daemon. Next, using in-house written R scripts, all Mascot data are compiled into a single Excel sheet in which information on toxins identified is sorted by fractionation time (i.e., retention time of elution for each toxin; all toxins have eluted over a series of subsequent wells during the high-resolution fractionation). From there, for each identified toxin, a script plots so-called Protein Score Chromatograms (PSCs), in which protein scores are plotted on the *y*-axis versus retention times of adjacent series of wells in which a toxin was fractionated on the *x*-axis. The peaks in all PSCs were subsequently integrated to yield semiquantitation results on the toxins in a venom under study. Finally, the obtained venomics and intact MS data could be correlated for additional toxin characterization.

In this study, we describe a high-throughput venomics workflow capable of performing full proteomic analysis of a snake venom within 3 days of analysis and data processing time and involving only several hours of manual labor. A graphical schematic representation of the complete workflow is presented in Figure 1. The procedure includes scripts for automated data processing and data sorting. The workflow starts by subjecting a snake venom to nanofractionation analytics, which involves liquid chromatographic separation of the toxins in a venom followed by a flow split to mass spectrometry (MS) analysis and to parallel high-resolution fractionation on a 384-well plate. After vacuum-centrifugation of the well plate to evaporate the eluents, a tryptic digestion procedure is performed directly on the well plate using automated pipetting steps. The well plate is then directly transferred to nanoLC–MS/MS for analysis using a fast-analytical gradient runtime of 14.4 min, resulting in 100 measurements per day. The proteomics data obtained are then automatically subjected to Mascot database searching using Mascot Daemon. Next, using in-house written scripts, all Mascot data are compiled into a single Excel sheet in which information on toxins identified is sorted by fractionation time (i.e., retention time of elution for each toxin; all toxins have eluted over a series of subsequent wells during the high-resolution fractionation). From there, for each identified toxin, a script plots so-called Protein Score Chromatograms (PSCs) in which protein scores are plotted on the *y*-axis versus retention times of adjacent series of wells in which a toxin was fractionated on the *x*-axis. Additionally, in a similar manner, Sequence Coverage Chromatograms can also be plotted if desired. A last script developed integrates the peaks in all PSCs to yield semiquantitation results on the toxins in a venom under study. Here, we demonstrated this new venomics strategy using venoms of three medically relevant snakes, analyzed under different chromatographic conditions, to evaluate and compare proteomics results. From there, HT venomics was performed on a set of eight other snake venoms. Our study demonstrates the feasibility of using high-throughput venomics to characterize the diverse toxins found in medically relevant snake venoms.

## Materials and Methods

### Chemicals, Stock Solutions, and Venoms

All chemicals and solvents used in this study were of analytical grade. Acetonitrile (ACN) and formic acid (FA) were purchased from Biosolve (Valkenswaard, The Netherlands), and water was purified using a Milli-Q plus system (Millipore, Amsterdam, The Netherlands). Iodoacetamide, β-mercaptoethanol, and ammonium bicarbonate were obtained from Sigma-Aldrich (Zwijndrecht, The Netherlands). For calibration, the mass spectrometry instrument ESI-L low concentration tuning mix from Agilent was used. Mass spectrometry grade modified trypsin was purchased from Promega Benelux B.V. (Leiden, The Netherlands) and stored and handled according to the manufacturer’s instructions. Snake venoms from *Echis ocellatus* (Nigeria), *Calloselasma rhodostoma* (captive bred, Thailand ancestry), and *Naja pallida* (Tanzania) were the main venoms used for evaluation, optimization, and method validation in this study and were sourced from animals held or previously held in the herpetarium at the Liverpool School of Tropical Medicine, UK. Venoms of eight additional snake species (see the Supporting Information Document 1 Table S1 for details) were also analyzed using the new HT venomics methodology. All venoms were stored in lyophilized form at −80 °C until reconstitution in water to prepare 5 mg/mL stock solutions, which were then aliquoted and stored at −80 °C until use.

### Liquid Chromatography, Nanofractionation, and Mass Spectrometry

Liquid chromatography separation with parallel post-column nanofractionation and mass spectrometry analysis was performed in an automated fashion. A Shimadzu UPLC system (‘s Hertogenbosch, The Netherlands) was used for the LC separations and was controlled by Shimadzu Lab Solutions software. Fifty microliters of each venom sample was injected by a Shimadzu SIL-30AC autosampler. The two Shimadzu LC-30AD pumps were set to a total flow rate of 500 μL/min. A 150 × 4.6 mm Waters Xbridge Peptide BEH300 C_18_ analytical column with a 3.5 μm particle size and a 300 Å pore size equipped with a C18 Guard Cartridge with a 5 μm particle size and a 300 Å pore size was used for separation of the venoms. The separations were performed at 30 °C in a Shimadzu CTD-30A column oven. Mobile phase A comprised 98% H_2_O, 2% ACN, and 0.1% FA or TFA, and mobile phase B comprised 98% ACN, 2% H_2_O, and 0.1% FA. The gradient used for the viper venoms consisted of a linear increase of mobile phase B from 0 to 30% in 5 min followed by a linear increase from 30 to 50% B in 25 min, which was then followed by an increase from 50 to 90% in 4 min. A 5 min isocratic elution at 90% B then followed, prior to final column equilibration for 10 min at starting conditions (100% A). The gradient used for the elapid venoms consisted of a linear increase of mobile phase B from 0 to 20% in 5 min and was followed by a linear increase from 20 to 40% B in 25 min, which was then followed by an increase from 40 to 90% in 4 min. A 5 min isocratic elution at 90% B followed next, and finally, the column was equilibrated for 10 min at starting conditions (100% A). The column effluent was split post-column in a 1:9 volume ratio. The smaller fraction was sent to a Shimadzu SPD-M30A photodiode array detector followed by a maXis QTOF mass spectrometer (Bruker Daltonics, Germany). An electrospray ionization (ESI) source was equipped onto the mass spectrometer and operated in positive-ion mode. The ESI source parameters were capillary voltage 3.5 kV, source temperature 200 °C, nebulizer at 0.8 Bar, and dry gas flow 6 L/min. MS spectra were recorded in the *m/z* 800–5500 range, in-source collision-induced dissociation (CID) was set at 200 eV, and 1 average spectrum was stored per s. Bruker Compass software was used for the instrument control and data analysis. The larger fraction was sent to a FractioMate fraction collector (SPARK-Holland & VU, The Netherlands, Emmen and Amsterdam). LC fractions (1 every 6 s or 1 every 12 s) were collected column by column in serpentine-like fashion on clear 384-well plates (Greiner Bio One, Alphen aan den Rijn, The Netherlands) using FractioMator software. The FractioMate allowed for four-well plates to be used for nanofractionation, enabling separation and nanofractionation of four venoms using 6 s fractions or eight venoms using 12 s fractions in one sequence. On each 384-well plate, one chromatographic run was collected into 368 wells when using 6 s fractions and two chromatographic runs of 184 wells when using 12 s fractions. After fractionation, the volatile contents of the plates were evaporated overnight for approximately 16 h using a Christ Rotational Vacuum Concentrator RVC 2–33 CD plus (Salm en Kipp, Breukelen, The Netherlands). The plates were then stored at −20 °C until tryptic digestion.

### High-Throughput in-Well Tryptic Digestion and NanoLC–MS/MS Analysis for Venom Proteomics

After LC–UV/MS with parallel high-resolution fractionation of venoms followed by vacuum centrifuging of the well plates containing nanofractionated venom toxins, 25 μL of reduction buffer (25 mM ammonium bicarbonate and 0.05% β-mercaptoethanol; pH 8.2) was added to plate wells by robotic pipetting using a ThermoFisher Multidrop. Next, plates were incubated at 95 °C for 15 min in the oven of a Hewlett Packard HP 6890 GC System, and the plates were then allowed to cool to room temperature, after which 10 μL of an alkylating agent was added (12.5 mM Iodoacetamide) using the same Multidrop. Next, the plates were incubated in the dark for 30 min at room temperature. Subsequently, a stock solution of trypsin (1 μg/μL in 50 mM acetic acid) was diluted 100 times in 25 mM ammonium bicarbonate to a concentration of 0.01 μg/μL of which 10 μL was added using the Multidrop, and the plates were incubated overnight at 37 °C. Next, the plates were centrifuged at 1000 rpm for 1 min in an Eppendorf Centrifuge 5810 R followed by addition of 10 μL of 1.25% formic acid to the plates, again using the Multidrop. Finally, the plates were analyzed using nanoLC–MS/MS (or stored at −20 °C until analysis).

For nanoLC separation of the tryptic digests, an UltiMate 3000 RSLCnano system (Thermo Fisher Scientific, Ermelo, The Netherlands) was used. The autosampler (with a capacity of three well plates in the autosampler) was run in partial-loop injection mode and allowed direct sampling from 384-well plates. Injection sequences were performed in identical serpentine fashion as described for the fractionation in the liquid chromatography, nanofractionation, and mass spectrometry section to allow for the most efficient data processing. The injection volume was set to 1 μL and injection was followed by separation on an Acclaim PepMap 100 C18 HPLC Column (150 mm × 75 μm) with a particle size of 2 μm and a pore size of 100 Å in combination with an Acclaim PepMap 100 C_18_ trapping column (5 mm × 0.3 mm), with a particle size of 5 μm and a pore size of 100 Å, obtained from ThermoFisher Scientific. The mobile phase comprised eluent A (98% water, 2% ACN, and 0.1% FA) and eluent B (98% ACN, 2% water, and 0.1% FA). The gradient used for the separation of the digests was 3 min isocratic separation at 1% B, linear increase to 40% in 7.5 min followed by a linear increase to 85% in 0.1 min, isocratic elution at 85% B for 0.7 min, linear decrease to 1% B in 0.2 min, and finally the column was equilibrated for 3.7 min at 1% B. The column was kept at 45 °C in the column oven. Mass detection was performed with a maXis QTOF mass spectrometer (Bruker Daltonics, Germany), which was equipped with a Bruker Captivespray source operating in positive-ion mode. The source parameters were source temperature, 150 °C; capillary voltage, 1.2 kV; dry gas flow, 3.0 L/min; and nanoBooster pressure, 0.20 Bar. Spectral data were stored at a rate of 2 Hz in the range of 50 to 3000 *m/z*. MS/MS spectra were obtained using CID in data-dependent mode using 10 eV collision energy. Bruker Compass software version 3.0 was used for instrument control and data analysis.

### Data Processing of NanoLC–MS/MS Data of Tryptic Digests for Venom Proteomics

The mass spectrometry data obtained were processed by using Bruker DataAnalysis software (version 5.1). By using the ProcessWithMethod function, all data files of the analyzed tryptic digests obtained for a single snake species were automatically processed into the desired MGF format. These resulting MGF files were then processed by using Mascot Daemon software (version 2.3.3) to process all the files in one batch for database searching, which was done by searching two different databases. 1: Uniprot database containing only Serpentes accessions and 2: Species-specific venom gland transcriptomic databases. Search parameters used were instrument type; ESI-QUAD-TOF, digestion enzyme; semiTrypsin, allowing one missed cleavage, carbamidomethyl on cysteine as a fixed modification, as variable modifications; amidation (protein C-terminus) and oxidation on methionine, fragment mass tolerance; ±0.05 and ±0.2 Da peptide for mass tolerance. In-house written scripts (with R),^20^ for which a user manual with example and all necessary files are available in the Supporting Information (Manual files.zip), were then used to extract and merge the information obtained from the Mascot searches with Daemon, resulting in a single Excel file for each of the snake venoms analyzed. The first script extracts comma-separated values (CSV) data from all Mascot search logs (which contain all the information obtained by the Mascot searches) and saves these data from each Mascot search as a separate CSV file using file names matching well identifier numbers of the wells containing the analyzed tryptic digests (all these resulting files are available in the Supporting Information folder “CSV files”). To facilitate this, the script reads a pre-made Excel file with information on which the Mascot search result (i.e., specific job number in the Mascot search log) corresponds to which well identifier number on a well plate. This script can be found in the Supporting Information as Script 1_export.R or Script1_export_2_Mascot2_8.R when using Mascot version 2.8. A template of such a pre-made Excel file is provided in the Supporting Information under the file named mascot_export_all_384_wells.xlsx. The second script filters out all relevant (script selectable) information from the CSV files and merges it into a single Excel file by plotting per protein retrieved from each well in separate worksheet columns: protein accession, protein score, sequence coverage, protein description, full protein sequence, found peptide sequences, and a link to the original Mascot search (all these files are available in a single Excel file in the Supporting Information named “All Mascot results”). This second script then plots this information sorted at fractionation retention time for the proteins found per well to obtain a clear overview of the present venom components in each well. Specifically, when multiple protein toxins are retrieved by Mascot for a tryptic digest result from a well, for each protein toxin found, a separate row is used in the Excel worksheet with the same retention time of fractionation in the first column. This script can be found in the Supporting Information under Script 2_merge_export_csv.R. A third script then plots protein scores (*y*-axis) from each of the detected venom proteins in all wells that they were detected in against the corresponding retention times of fractionation (*x*-axis) to generate so-called PSCs. Additionally, this third script calculates the peak area of each of the separate PSCs to allow for semiquantification of the relative abundance of the proteins in venoms. The third script can be found in the Supporting Information under Script 3_Protein Chromatogram plotting and Integration.R. The fourth and final script (found in the Supporting Information as Script 4_ combine *Y* values protein chroms.R) combines all the *X* and *Y* information of all the individual proteins found, generated with script 3, in a single Excel file to facilitate plotting all the data in one graph in Excel or as done in this study, in Graphpad Prism version 8. All scripts developed, merged files, CSV files, pie charts, PSCs, PSC peak areas, UV data, and all GraphPad files used in this study are available in the Supporting Information.

## Results and Discussion

### Proteomics Data Processing

**Figure 2:**
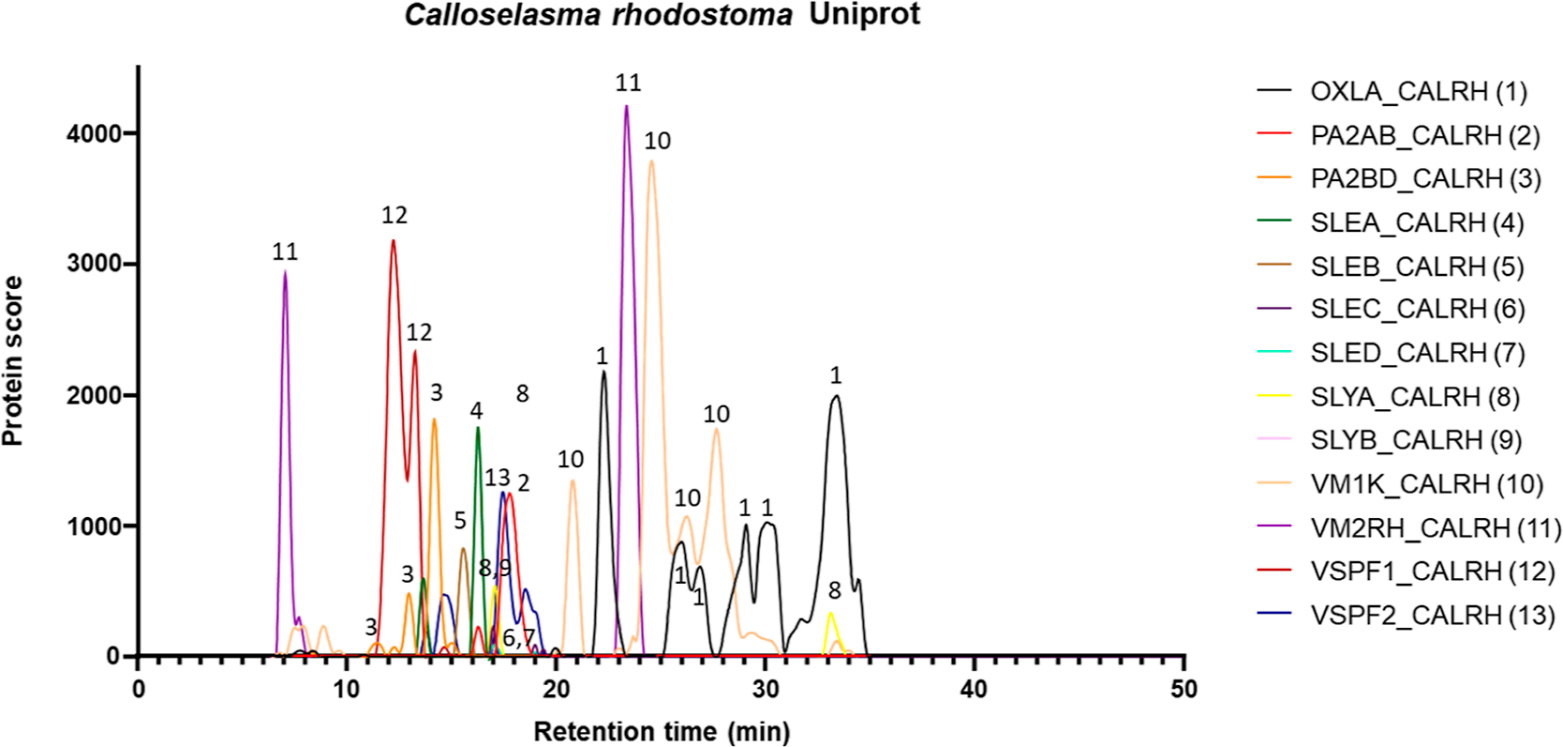
PSCs of the *Calloselasma rhodostoma* venom. The protein scores from each of the toxin IDs obtained with Mascot database searching are plotted against the retention time from the wells they were detected in. Each of the individual toxin traces is numbered with its corresponding protein identifier on the right. This results in so-called PSCs, which can, for example, be used as a method for the identification of venom toxins through different detection methods.

In this study, a high-throughput venomics workflow was developed and applied, which allowed for rapid, full, proteomic analysis of a snake venom within 3 days, with the capability of identifying more than 50 proteins with varying molecular weights. The high-resolution fractionation ability of the nanofractionation setup reduces the sample complexity of the collected fractions but, in turn, results in many samples per well plate. To keep this methodology high throughput, it was necessary to automate the tryptic digestion workflow, which was achieved by performing tryptic digestions directly on the well plates using a pipetting robot for every step. Due to the high resolution of fractionation, the resulting reduced sample complexity allowed for a significant reduction in nanoLC–MS/MS runtimes, down to 14.4 min, and enabling 100 measurements per day. The obtained nanoLC–MS/MS data were processed in batch with Bruker DataAnalysis software, resulting in MGF files. These MGF files were in turn run through both Uniprot (Serpentes taxonomy) and through venom gland transcriptome databases by using Mascot software with Mascot Daemon. To rapidly process the resulting Mascot search data, custom scripts were developed. The first script extracted all Mascot database search information from each tryptic digest from the Mascot server, sorted it by well number, and saved it as CSV files with well identifier numbers as file names. The second script merged the contents of all the CSV files into one Excel file and sorted the information according to fractionation retention time by well number. The following information was collected for each retrieved result from each well: species, protein accession, protein score, protein mass, protein sequence coverage, protein description, full protein sequence, found peptide sequences, and a link to the online search result. An example can be found in the Supporting Information (i.e., document Example merged CSV files Bothrops asper.xlsx). The third script extracted the protein scores of each individual venom toxin retrieved from each tryptic digest and sorted them by retention time, allowing for the construction of so-named PSCs (i.e., the plotting of protein score on the *y*-axis vs. retention time on the *x*-axis for each venom toxin). This resulted in representation of the proteomics data in a visually appealing and easy-to-interpret manner and, to the best of our knowledge, is the first time such data are presented this way. See Figure 2 for an example of such PSCs. The third script simultaneously calculated the peak areas of all peaks in the PSCs, allowing for semiquantification of toxin abundances. The fourth and final script combined all the *X* and *Y* information generated with script 3 of all the individual proteins found in a single Excel file to facilitate plotting all the data in Graphpad Prism in one simple step.

### Combining the Processed Proteomics PSC Data with LC–UV and LC–MS Data

**Figure 3:**
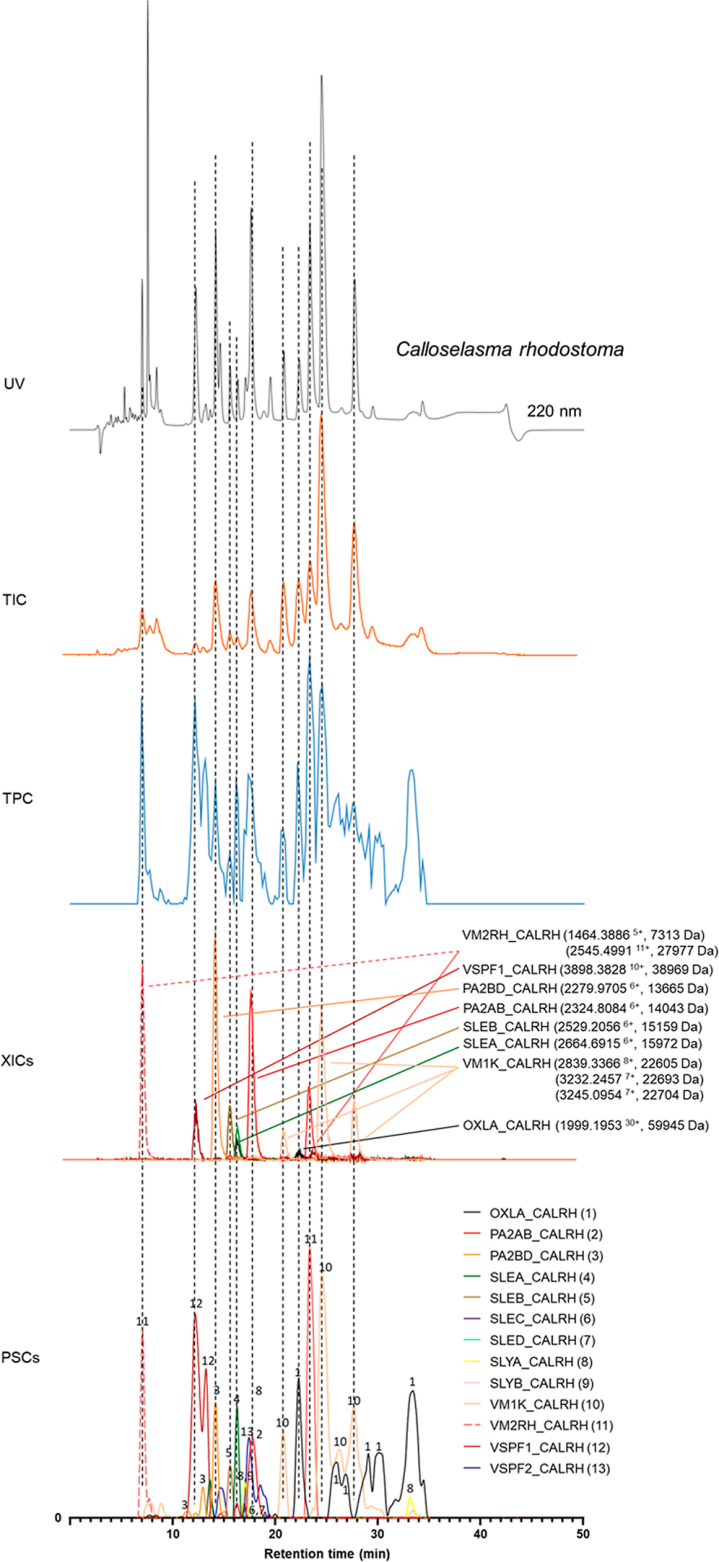
LC–UV-MS-PSC data superimposed from *Calloselasma rhodostoma* venom. The data for *C. rhodostoma* venom from the three detection techniques used in this study were superimposed to obtain a comprehensive figure that facilitates the identification of venom toxins. The top graph shows the UV (220 nm) trace. The second graph shows the total ion chromatogram (TIC) from the mass spectrometry data. The middle trace shows the total protein chromatogram (TPC) consisting of the sum of all protein scores obtained from the proteomics data. The penultimate graph shows the extracted ion chromatograms (XICs) obtained from the mass spectrometry data. The XICs shown here are believed to match to the toxin IDs found in the bottom graph (PSCs) based on their matching retention times and peak shapes. The bottom graph shows the PSCs, which represent the individual venom proteins found with the Mascot database searching of the digested contents in the wells.

The presented methodology comprises a combination of analytical and proteomics methods that each provides a unique data set. LC–UV, LC–MS, and PSC data sets can be correlated, thereby resulting in comprehensive figures comprising superimposed chromatograms from each of the distinct data sets. The LC–UV data are used as a reference point for the separation, providing initial information on snake venom composition, and can be used for semiquantitative analysis when combined with complementary MS and proteomics data. After LC–UV, MS analysis was performed, which allows for accurate masses to be assigned to venom toxins observed in the UV trace, from which tentative toxin classes can be proposed. Via an implemented post-column flow split, eluting venom toxins are also fractionated onto 384-well plates, subjected to robotically operated tryptic digestion, and then analyzed with nanoLC–MS/MS for venom proteomics. These data obtained are then processed into PSCs (as discussed in the previous section). The chromatographic data sets are finally combined into one comprehensive figure (see Figure 3 for an example), providing a detailed and easily interpretable overview of the venom composition.

For all venoms analyzed in this study, Uniprot database searching was performed and, when a species-specific venom gland transcriptome database was available, this database was also searched to improve resolution. Both these searches gave complementary results. The transcriptomics database allows the searching of the transcriptome of the venom gland of the same species as the venom proteome under analysis, thereby resulting in the most accurate protein matches, and we utilized this approach for *E. ocellatus*,^21^*C. rhodostoma* (unpublished transcriptome data), *Naja pallida*,^22^*Naja naja*,^22^*Naja nigricollis*,^22^ and *Naja mossambica*.^22^ On the other hand, these searches only provide non-informative protein identified numbers. While Uniprot will only retrieve the toxins of which detailed information is available in the database, it does also retrieve results of venom toxins from similar species, thus potentially overcoming a limitation with an incomplete species-specific database or where intraspecific venom variation might occur. Importantly, Uniprot also conveniently provides valuable information on toxin families and toxin names and on publications describing the toxins retrieved. Also, additional information, such as toxin functionality and/or in vitro assay bioactivities, can easily be identified if associated with the proteins deposited in Uniprot. In addition, we compared the ability of Mascot database searches performed using the Uniprot or transcriptomic databases to provide toxin identifications. The conclusion was that transcriptomic database searches resulted in three times more toxin identifications compared to Uniprot database searches (56 for Uniprot vs 163 for transcriptomic summed across six species). However, as mentioned above, Uniprot provides valuable information on toxin families and toxin names and on publications describing the toxins retrieved. These evaluations are described in detail in Supporting Information document 1 Section 1.

### Optimization and Evaluation of the Analytical Methodology

#### Robotic versus Manual Tryptic Digestion

To make this methodology high throughput, the time required to tryptically digest all wells on 384-well plates had to be substantially reduced. Therefore, all manual steps in the digestion protocol were performed using a pipetting robot. Both manual and automated tryptic digestion were performed on *C. rhodostoma* and *E. ocellatus* venoms. Downstream results demonstrated no substantial differences observed between the two methods in terms of the toxins recovered, with 24 vs 22 toxins detected via transcriptome database searches with the manual and automated digestion approaches, respectively, for the *C. rhodostoma* venom and 29 vs 28 toxins detected for the *E. ocellatus* venom. These data therefore validated the use of an automated tryptic digestion step in our analytical pipeline. These evaluations are described in detail in Supporting Information document 1 Section 1.

#### NanoLC–MS/MS Gradients

Snake venoms were fractionated in high resolution on 384-well plates to enable subsequent proteomic analysis on tryptic digested fractions. This approach limits the average number of toxins collected in each well typically to only a few per well and thereby decreases sample complexity and facilitates very short LC runtimes. In our study, LC runtimes of 14.4 min per run were used, which translate to a capacity of 100 nanoLC runs per day. The effect of these short runtimes on downstream proteomics results was evaluated in quadruplicate for well N5 from *C. rhodostoma*. Our resulting data demonstrated that a runtime of 14.4 min does not have a substantial impact on toxin resolutions obtained with longer LC runtimes. Protein scores found in the 14.4 min runs were on average 38% lower than the 60 min runs. For the sequence coverages, it was found that on average 19% less sequence coverage was found for the toxins with the 14.4 min runs as compared to the 60 min runs. However, the same proteins were retrieved in the 14.4 min analyses and from the 60 min analyses. These experiments are discussed in detail in Supporting Information document 1 Section 2.

#### LC–MS with Nanofractionation

Generally, FA is used as an acidifier while performing LC–MS experiments due to its positive effect on LC separation and MS ionization. TFA is another acidifier that is frequently used in LC and can achieve higher separation resolution than FA. However, despite this advantage, TFA is rarely used in measurements that include MS due to its negative impact on ion suppression. In this study, it was shown that TFA can be successfully used for LC–MS analysis of snake venom. This was achieved due to the post column flow split implemented in the at-line nanofractionation setup, which allows only 10% of the total flow to be sent to the MS, resulting in elimination of ion suppression created by TFA. These experiments are discussed in detail in Supporting Information document 1 Section 3.

#### Comparison of Proteomics Results after LC–MS Runs with FA or TFA as an Acidifier

We compared the effect of the different eluent acidifiers on the resulting proteomics data obtained after LC–MS runs with FA and TFA. To do so, nanofractionated venom toxins from *C. rhodostoma*, *E. ocellatus*, and *Naja pallida* were subjected to the tryptic digestion procedure followed by nanoLC–MS/MS analyses, database searching, and script-controlled data processing. All PSC results obtained for these three species, via database searches of the Uniprot database, are provided in the Supporting Information document: All PSCs.pzfx. The overall conclusion drawn from these experiments is that comparable results were obtained for both FA and TFA for each of the three venoms analyzed. The use of TFA resulted in higher numbers of total venom toxins identified (FA Uniprot: 27. FA transcriptomic: 70. TFA Uniprot: 29. TFA transcriptomic: 79) and improved chromatographic resolution of the separated toxins. A detailed description of these results is provided in Supporting Information document 1 Section 4.

#### Resolution of Fractionation and NanoLC–MS/MS Analysis Time per Venom

The resolution of fractionation is an important parameter that influences sample complexity and determines the number of wells used for fraction collection. In addition to the standard 6 s resolution used, a resolution of 12 s was also tested for the venoms of *C. rhodostoma*, *E. ocellatus*, and *Naja pallida*. Lowering the resolution to 12 s decreased the number of wells needed for fractionating a complete venom by 50%, which subsequently led to a 50% decrease of nanoLC–MS/MS data analysis/processing time. In addition, it was found that the lower fractionation resolution (i.e., 12 s) did not compromise the resolution of the PSCs as comparable results were achieved. The total combined numbers of venom toxins retrieved for these three venoms at 12 s vs 6 s resolution were 32 and 29, respectively, however with approximately 1.6x higher protein scores for the 12 s fractionation (i.e., more toxin was collected per fraction) and with similar sequence coverages (deviation ∼10%). A detailed description including corresponding figures is provided in Supporting Information document 1 Section 5.

### Demonstration of the Analytical Methodology

#### Processing Proteomics Data

**Figure 4:**
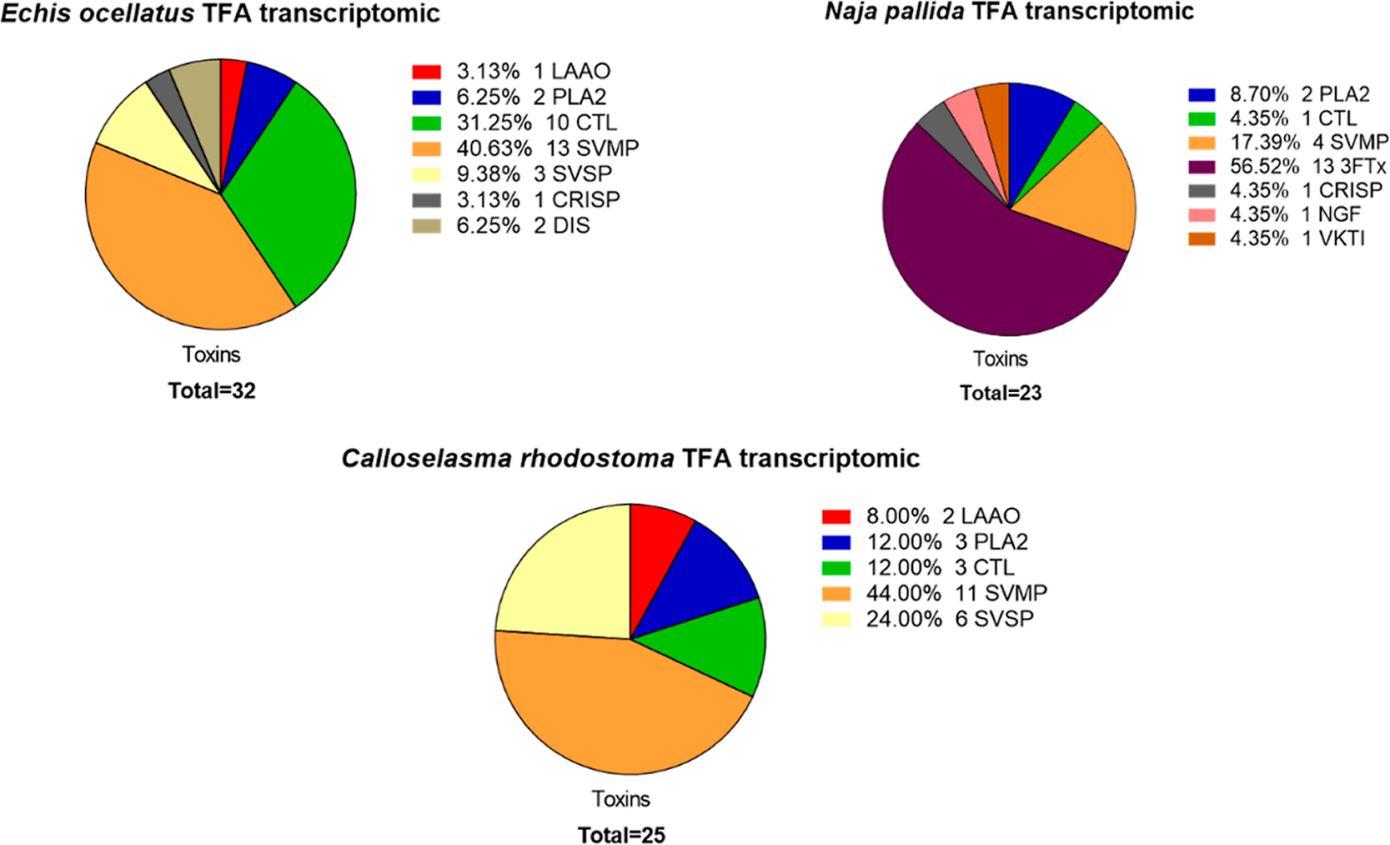
Pie charts showing the number of toxins identified and their respective toxin families when using the transcriptomic databases for venom sourced from *Calloselasma rhodostoma*, *Echis ocellatus*, and *Naja pallida.*

All proteomics results were also processed visually into pie charts quantifying relative venom toxin isoform abundance. The pie charts from the database searches using the transcriptomics databases are provided in Figure 4. The pie charts from the database searches using both the transcriptomics databases and the Uniprot database are shown in Supporting Information document 1 Section 7a: Pie charts of toxins found for each HT Venomics analysis. For each analysis, a separate pie chart shows the toxins recovered, sorted by the number of toxins per toxin family. Our findings demonstrated that the breadth of venom toxin families typically found distributed across viperid and elapid snake venoms were detected (i.e., SVMP, PLA_2_, CTL, SVSP, LAAO and 3FTx, PLA_2_, SVMP, and VKTI).^12^ All information from all analyses on all exact toxins retrieved including sequence coverages, protein scores, and peptides found per toxin can be found in Excel document: All Mascot results.xlsx in the Supporting Information.

#### Studying Similar Toxins and Post-Translational Modifications

**Figure 5:**
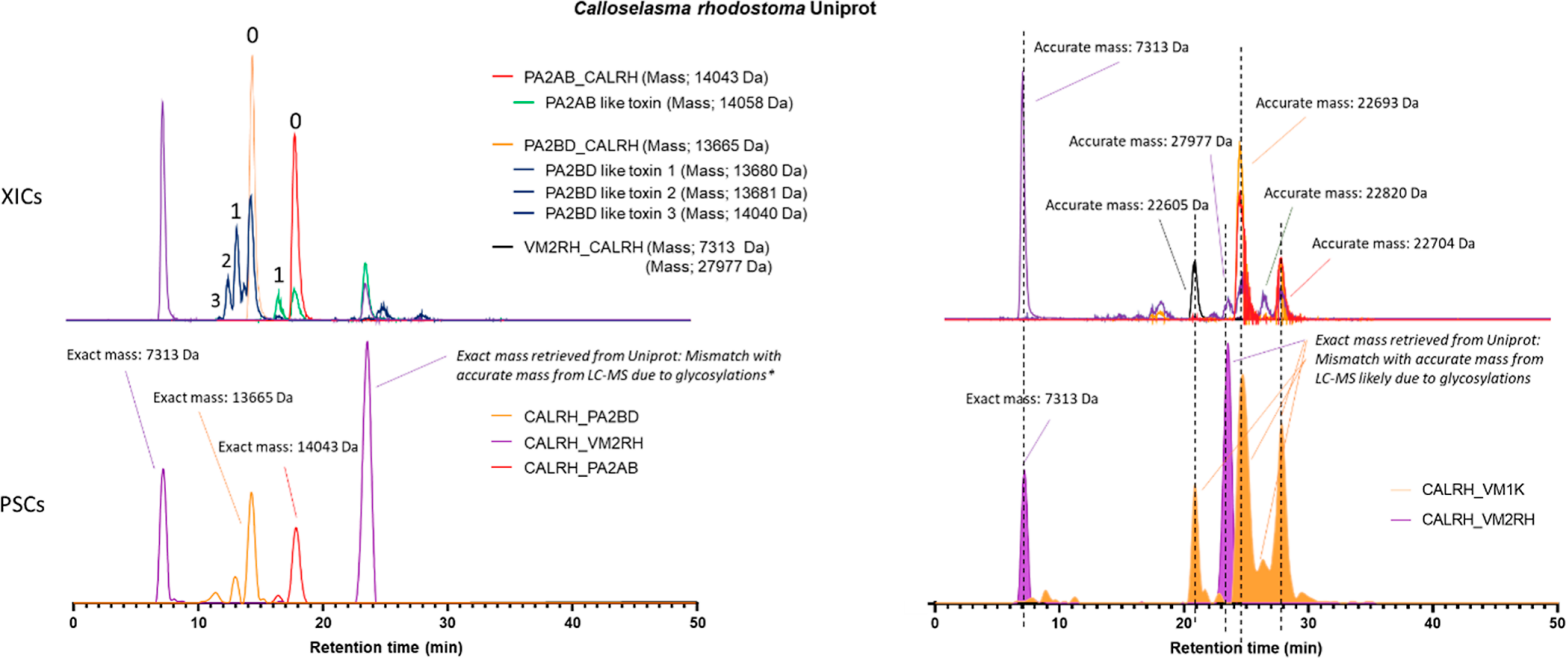
Detection of multiple PSC peaks from *Calloselasma rhodostoma* venom corresponding to a single protein accession. Bottom Graphs (C,D) show PSCs of venom toxins PA2BD (Phospholipase A_2_), PA2AB (PLA_2_), and VM2RH (snake venom metalloproteinase). Upper graphs (A,B) show XICs and their accurate masses correlating to the PSCs. In graph C, toxin VM2RH shows two peaks of which one corresponds to the disintegrin rhodostomin from which the exact mass can be exactly matched to an accurate mass in the MS (graph A), contrary to its SVMP rhodostoxin from which the exact mass cannot be determined due to its exact glycosylations being unknown. In graph C, toxin PA2BD displays three peaks of which the largest peak can be exactly correlated to the correct accurate mass (0) in graph A. The other PSC peaks corresponding to toxin PA2BD correlate to other accurate masses (1,2,3) shown in graph A. This could be due to PTMs or sequence similarities in different toxins present that are not yet known in the database and therefore are recognized to their closest homologue (PA2BD). In graph C toxin PA2AB displays two peaks from which the largest peak can be exactly correlated to the correct accurate mass (0) in graph A. The other PSC peak corresponding to toxin PA2AB correlates to the other accurate mass (1) shown in graph A. This could be due to PTMs or sequence similarities in the other toxin present that is not yet known in the database and therefore is recognized to its closest homologue (PA2AB). Graph D shows PSCs from SVMPs VM2RH and VM1K with multiple peaks. The two VM2RH peaks can be explained by one that corresponds to its disintegrin rhodostomin and the other to the SVMP rhodostoxin. For VM1K, there are multiple peaks present that correlate to different accurate masses, which are most likely different toxins with sequence similarities but are not yet known in the database and therefore are recognized to their closest homologue (VM1K).

Analysis of the resulting PSCs derived from the venom analyses revealed, in some cases, multiple related peaks or a single relatively broad peak. These profiles are likely the result of either different proteins with high sequence similarities (i.e., isoform variants) or the same protein with different post-translational modifications (PTMs). After tryptic digestion and proteomics analysis, the same peptides were often retrieved from adjacent peaks, thereby resulting in the same toxin in a PSC apparently eluting as different peaks. When analyzing the corresponding LC–MS data, it was commonly observed that at those times when different PSC peaks corresponded to the same protein, different masses were detected. Combined, these data support the hypothesis that these proteins are either highly similar to one another or reflect detection of the same protein containing PTMs. This combined set of information can be used to predict potential toxin PTMs based on mass differences, which in turn can guide the selection of secondary analytic methods specific to characterizing a PTM. For cases where the same masses are detected, this may be an artifact of conserved digested peptides that are shared across highly related toxin isoforms being detected proteomically. A viable solution to this challenge is to re-measure the samples on a higher-resolution mass spectrometer, and since all well plates with tryptic digests described in our approach can be stored for long time periods at −20 °C after nanoLC–MS/MS analysis, they can also be re-analyzed using another analytical setup later in time. Optionally, at the cost of time, using a longer nanoLC–MS gradient could also be used to get higher sequence coverages that provide the requisite differentiation. Some typical results involving multiple peaks found in a PSC are shown in Figure 5. In this figure, PSCs from the Uniprot database are plotted next to the relevant XICs.

#### Quantification Possibilities for HT Venomics

**Figure 6:**
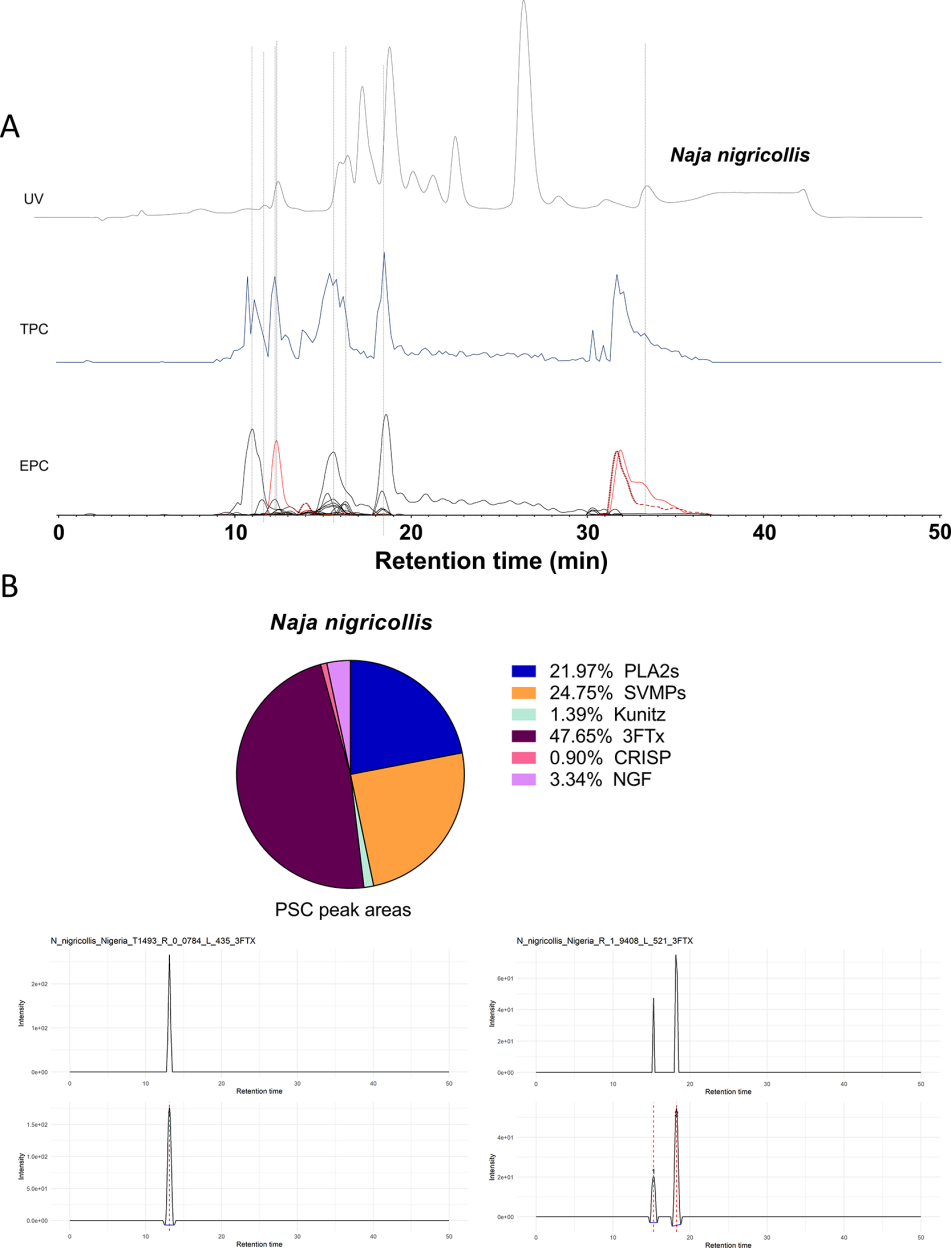
Proteomics analysis and venom composition analysis by PSC peak integration of *Naja nigricollis*. (A) Superimposed data of UV, TPC, and EPC data from the *Naja nigricollis* venom used to correlate UV observed peaks to toxins identified by proteomics. (B) Pie chart of the venom composition of *Naja nigricollis* based on the PSC peak areas of the identified proteins and their respective toxin families. In addition, two examples of PSC peak integration are shown to illustrate the integration process of the script.

**Figure 7:**
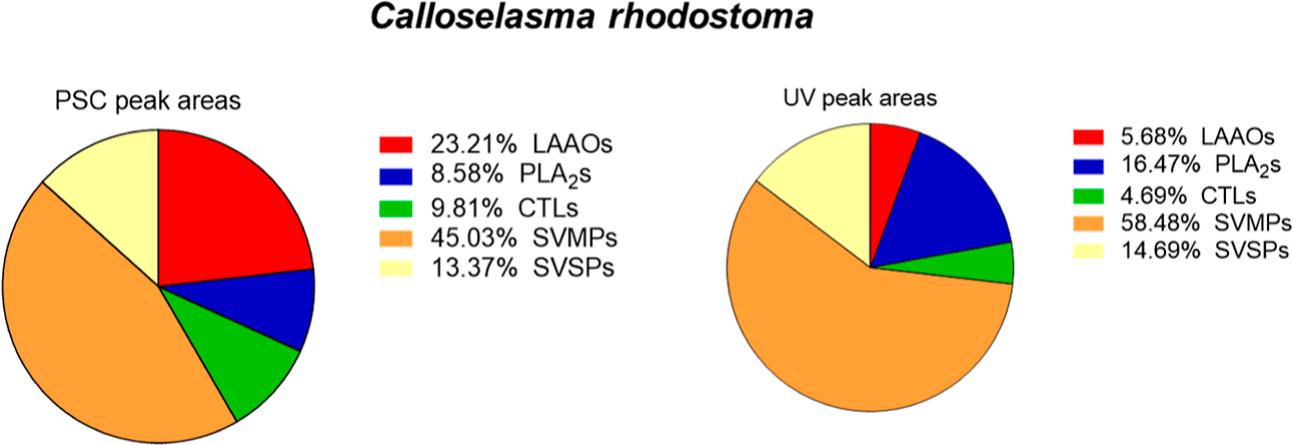
Comparison PSC peak areas and UV peak areas as means for semiquantitation for the venom composition of *Calloselasma rhodostoma*. The two pie charts show the venom composition based on the integrated PSC and UV peak areas and for each of the found proteins and their respective toxin classes. Comparable results were obtained for both methods regarding the SVSPs (snake venom serine protease) and SVMPs, while more deviation was observed for the LAAOS, PLA_2_s, and CTLs (C-type lectins).

Our described HT venomics strategy has clear advantages, namely, its intrinsic throughput and almost fully automated operational and data processing characteristics. In contrary to other venomics studies, this approach allows analysis and comparison of many different venoms rapidly, which could facilitate the study of inter- and intra-specific venom variation. Traditional venomics approaches retain certain strengths; despite being elaborate and time consuming, they have proven to deliver qualitative and comprehensive data, including most recently the absolute quantitation of all venom toxins in a given venom.^14,23,24^ However, the ability of any venomics approach to identify all venom toxins, including those of low abundance, depends on the brand and model, and ionization and fragmentation capabilities of the mass spectrometer(s) used in a study, making comparisons of different studies challenging. Although our HT venomics methodology retrieved the majority of toxins present in a venom when performing database searches using species-specific transcriptome databases, we next wished to assess the quantitation potential of the current methodology. To do so, we used the venom of *Naja nigricollis*, for which absolute venomics data are published,^14^ and applied our HT venomics approach with TFA as the acidifier. The resulting superimposed LC–UV and PSC data are shown in Figure 6A. Using the script developed in this study, which can integrate the peaks from the PSC data as well as sum all the protein scores found for each toxin, we analyzed the results and revealed comparable data outputs as displayed in Supporting Information document 1 Figure 7c: Comparison PSC peak area and protein score summing. The peak area method was used for further analysis since it was the most representative in terms of general quantification as shown in Figure 6B. Following the calculation of toxin peak areas, toxins were sorted into toxin families, after which the total peak area per toxin family was calculated. From there, a pie chart was constructed, which represents the relative abundance of each toxin family. Subsequently, the LC–UV data were used to facilitate semiquantification of proteins, i.e., when UV data are acquired at 220 nm (mainly amide bond absorption) and/or at 254 and 280 nm (aromatic ring absorption of aromatic amino acids). For this, the peaks found in the PSCs were overlaid with the LC–UV data (220 nm), and UV peaks with the same retention time and peak shape were integrated. In some cases, when overlapping co-eluting toxins were present such as observed with *N. nigricollis*, we faced difficulties in assessing the peak areas from the LC–UV data and which impaired robust correlations between PSC and UV data. However, such correlations between the PSC and UV data were achievable with the *C. rhodostoma*venom as shown in Figure 6.

**Figure 8:**
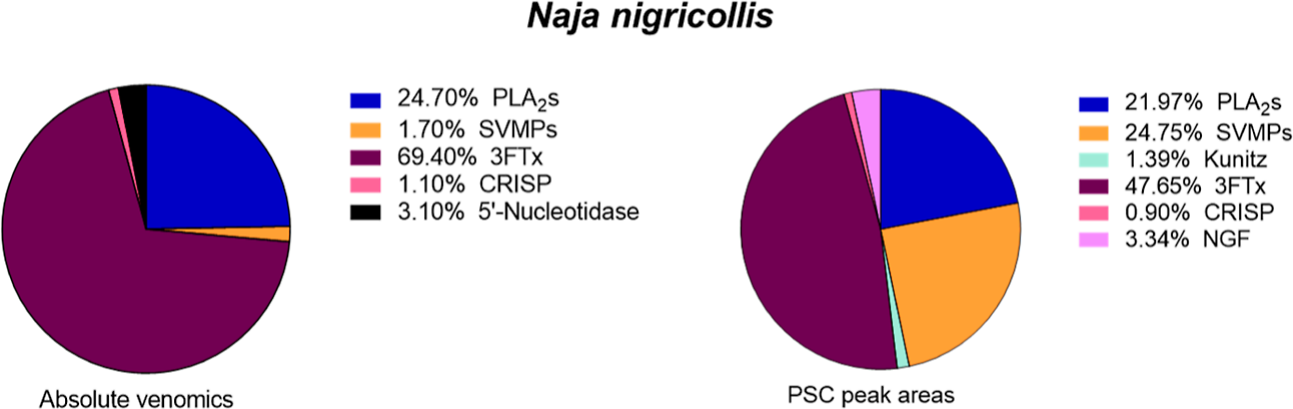
Comparison of the venom compositions of *Naja nigricollis* obtained absolute venomics and high-throughput venomics approaches. The two pie charts show the venom composition based on the absolute venomics from Calderón-Celis et al. (2017) and the integrated PSC peak areas for each of the found proteins and their respective toxin classes from this study. Comparable results were obtained for both methods regarding the PLA_2_s and CRISPs, while more deviation was observed for the 3FTx’s and SVMPs.

Finally, the absolute venomics data from Calderón-Celis et al.^14^ were used to visualize *N. nigricollis* venom toxin families by their measured abundances, resulting in comparable pie chart summaries between the different approaches (Figure 8). When comparing the data retrieved from the PSCs with the LC–UV data pie chart, similar results were obtained for the majority of toxin families, except for the SVMPs as shown in Figure 7. In the study by Calderón-Celis et al., an ESI-QTOF mass spectrometer was used for the identification of intact venom proteins. The detection of large intact proteins with this instrumentation is challenging due to ionization efficiencies and toxin abundances. This likely explains the considerable increased SVMP identifications in this study, which was performed based on proteomics approaches. In combination, these results demonstrate the potential of the current HT venomics strategy to facilitate semiquantitation of toxins found in snake venoms. Finally, it should be noted that absolute venomics can also allow absolute quantities of venom toxins to be analyzed via ICP triple quadrupole MS and 32S/34S isotope dilution analysis.^14^ Our described HT venomics strategy is currently not capable of performing absolute quantification as internal standards and/or labeling approaches are required. To allow real quantitation, non-venom toxins or pure toxins from a different venom than the venom under study could be spiked into a venom prior to analysis and used as internal standards.

#### Application of HT Venomics on Diverse Snake Venoms

**Figure 9:**
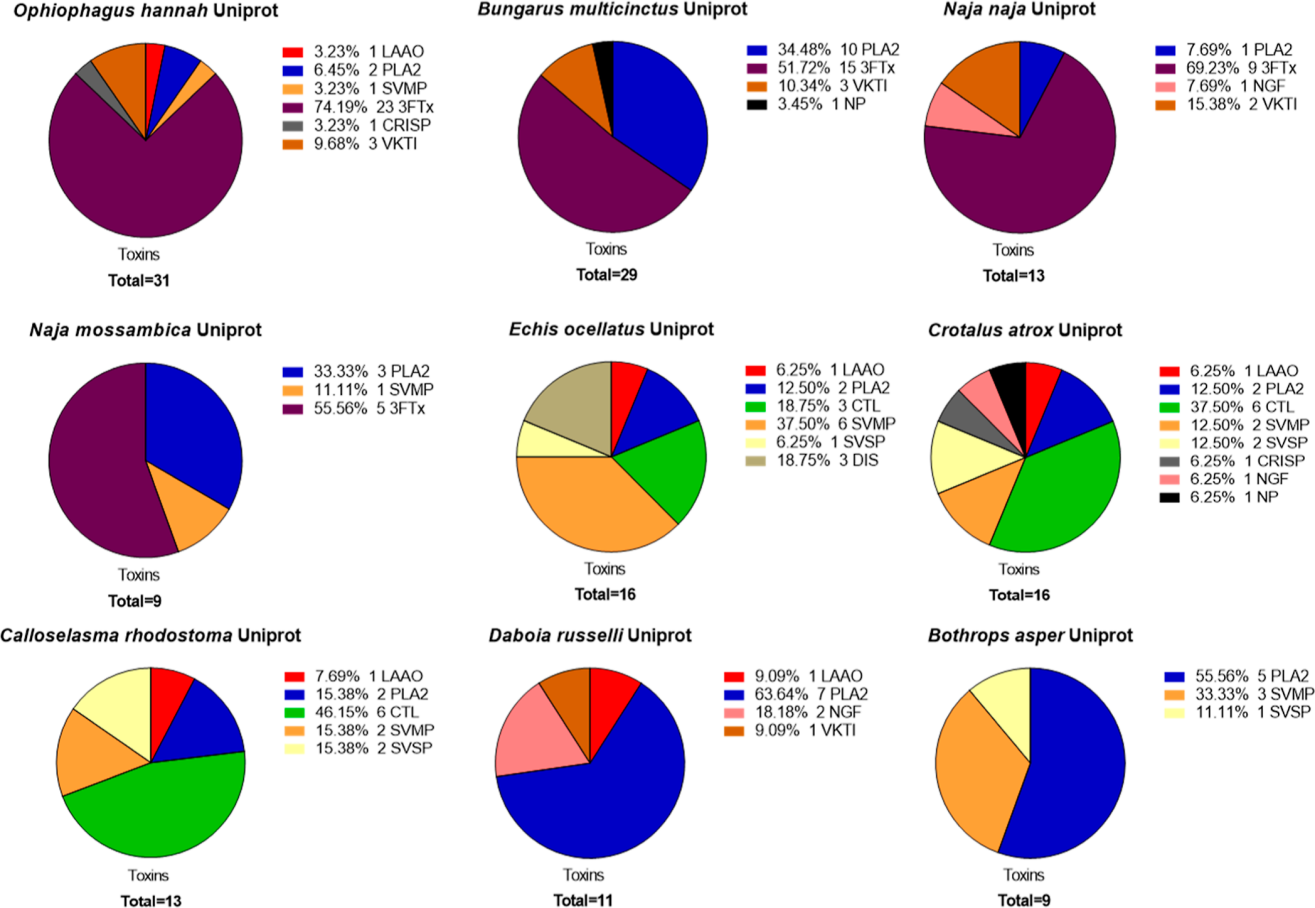
Pie charts of venom composition derived from medically relevant snake species obtained through high-throughput venomics analysis. After the proteomics data are obtained and processed into PSCs, as described in the Materials and Methods, the number of toxins and their respective toxin families were compiled into summary pie charts. The proteomics results obtained through the Uniprot database were used rather than the transcriptomic databases due to the absence of transcriptomic databases for several snake species. In addition, the results of *Naja nigricollis* and *Naja pallida* are not shown here due to a limited number of toxins present in the Uniprot database (two and three, respectively; though see Supporting Information document 1 Section 7 for details).

Finally, to provide venom researchers with an initial HT Venomics data set of value for future comparative analysis, venoms sourced from a diversity of medically relevant snake venoms were analyzed and processed using the HT Venomics approach (Figure 9). The venoms utilized were from *C. rhodostoma*, *E. ocellatus*, *B. asper*, *Crotalus atrox*, *Daboia russelii*, (all vipers), *Bungarus multicinctus*, *Naja pallida*, *N. naja*, *N. nigricollis*, *N. mossambica*, and *Ophiophagus hannah* (all elapids), thus incorporating representative viper and elapid venoms from across the world. Full details on these venoms and their analysis with HT Venomics can be found in Supporting Information Table S1, where specific information is provided on the gradient used, FA or TFA used for the LC separation, if a species-specific transcriptomics database was available, and if LC–MS data were acquired and on which mass spectrometer. Of the data acquired, all LC–UV chromatograms, CSV files, and script-processed data such as the PSCs and PSC peak integration results are provided in the Supporting Information. When measured and processed, the Supporting Information document also contains superimposed UV-MS-PSC data in Section 7g: Superimposed PSCs, UV and MS data for *C. atrox*, *E. ocellatus*, *N. nigricollis*, and *N. pallida* venom analyzed under optimized HT Venomics conditions. Note that the higher the toxin mass, on average, the lower the MS sensitivity in traditional LC–MS analyses will become. For this reason, the larger toxins including venom proteases are less likely to be observed in the LC–MS data. In summary, for viper venoms, a total of 65 toxins were identified of which PLA_2_s, CTLs, and SVMPs were the most dominant toxin families accounting, on average, for 28, 23, and 20% percent of the venom contents. In the elapid venoms, a total of 87 toxins were found of which the 3FTx and PLA_2_ toxin families were the most dominant, representing 63 and 21% of the total venom composition. When compared to global summaries of snake proteomes published in the literature, the findings obtained here are highly representative as the same abundant toxin families are recovered.^12,25^

## Conclusions

In this study, a new venom characterization workflow, named high-throughput Venomics, is introduced. A detailed conclusion describing the method evaluation, optimization, and validation of the procedure is provided in Supporting Information document 1 Section 6. Our workflow allows rapid and efficient semiautomated profiling of venoms to obtain their respective venom proteomes. These proteomes are conveniently plotted as so-called PSCs, which facilitate direct correlation with parallelly obtained LC–MS and LC–UV data of the venom. Automation of data processing and data plotting into easily interpretable formats is performed by in-house custom scripts, including enabling the integration of peaks detected in the PSCs for semiquantitation purposes. By comparing multiple PSC peaks in one PSC TSP plot with peak shape- and retention time-matching XICs from the parallel acquired LC–MS data, venom toxin PTMs can be investigated further. In addition to characterizing the toxin variations that exist between different medically important snake species, for the field of (anti)venomics, this methodology could greatly assist in developing the next generation of antivenoms due to its high-throughput/resolution capabilities to identify the toxins bound and not bound by existing snakebite therapies.

## Supporting Information Available

Snake venoms used in this study with information on their origin and analytical information; evaluation of robotically operated tryptic digestion and Mascot database searching complementarity of Uniprot versus Transcriptomic; nanoLC–MS/MS gradients; LC–MS with nanofractionation; comparison of proteomics results after LC–MS runs with FA or TFA as the acidifier; resolution of fractionation and nanoLC–MS/MS analysis time per venom; additional data; pie charts of toxins found for each HT venomics analysis; PSCs of *Naja nigricollis* 12 s TFA Mascot searches; comparison PSC peak area and protein score summing; comparison of 6 s and 12 s fractionation for three species; comparison between FA and TFA as the acidifier in LC–UV–MS separation; charge states of FA and TFA for *Calloselasma rhodostoma* toxins; comparison of charge states of venom toxins when using FA and TFA; comparison of the proteomics results between the 60 and 14.4 min nanoLC gradient; superimposed PSCs, UV, and MS data for *C. atrox*, *E. ocellatus*, *Naja nigricollis*, and *Naja pallida* analyzed under optimized HT venomics conditions; superimposed PSCs and UV data for *B. multicinctus*, *D. russelii*, *Naja*, *O. hannah*, *Naja mossambica*,and *Bothrops**asper* analyzed under optimized HT venomics conditions (PDF)
R Scripts.zip (ZIP)
All UV Data for paper.zip (ZIP)
mascot_export_all_384_wells.xlsx (XLSX)
Example merged CSV files *B. asper*.xlsx (XLSX)
CSV Files.zip (ZIP)
PSC Peak areas.zip (ZIP)
All PSCs.zip (ZIP)
Manual files.zip (ZIP)
