# Supplementary material for: High-Throughput Venomics
Source: J Proteome Res. 2023 Apr 3;22(6):1734–46. doi: 10.1021/acs.jproteome.2c00780 (PMC10243144; doi:10.1021/acs.jproteome.2c00780)
Supplement: Supplementary file 1 — pr2c00780_si_001.pdf [file pr2c00780_si_001.pdf]

Supplementary Information Document 1

High throughput venomics

Julien Slagboom[1], Rico J. E. Derks[2], Raya Sadighi[1], Govert W. Somsen[1], Chris Ulens[3], Nicholas R. Casewell[4], Jeroen Kool[1,*]

[1] Amsterdam Institute of Molecular and Life Sciences, Division of BioAnalytical Chemistry, Department of Chemistry and Pharmaceutical Sciences, Faculty of Science, Vrije Universiteit Amsterdam, De Boelelaan 1085, Amsterdam 1081HV, The Netherlands

[2] Center for Proteomics and Metabolomics, Leiden Universitair Medisch Centrum, Albinusdreef 2, 2333 ZA Leiden, The Netherlands

[3] Laboratory of Structural Neurobiology, Department of Cellular and Molecular Medicine, Faculty of Medicine, KU Leuven, 3000, Leuven, Belgium.

[4] Centre for Snakebite Research and Interventions, Liverpool School of Tropical Medicine, Pembroke Place, Liverpool L3 5QA, UK

*Corresponding author. Tel.: +31 20 5987542.
E-mail address: j.kool@vu.nl (Jeroen Kool).

Table of Contents

Suppl.Table 1 Snake venoms used in this study with information on their origin, and analytical information
Section 1. *SI Evaluation of robotically operated tryptic digestion and Mascot database searching complementarity of Uniprot versus Transcriptomic.*
Section 2. *SI nanoLC-MS/MS gradients*
Section 3. *SI LC-MS with nanofractionation*
Section 4. *SI Comparison of proteomics results after LC-MS runs with FA or TFA as acidifier*
Section 5. *SI Resolution of fractionation and nanoLC-MS/MS analysis time per venom*
Section 6. *SI Conclusion*
Section 7. *SI Figures & Tables*
7a: Pie charts of toxins found for each HT Venomics analysis
7b: Protein Score Chromatograms of *Naja Nigricollis* 12 sec TFA Mascot searches
7c: Comparison PSC peak area and protein score summing
7d: Comparison 6sec and 12sec fractionation for 3 species
7e: Comparison between FA and TFA as acidifier in LC-UV-MS separation
7f: Charge states FA and TFA for *Calloselasma rhodestoma* toxins.
Suppl. Table 2. Comparison charge states of venom toxins when using FA and TFA.
Suppl. Table 3. Comparison of the proteomics results between the 60 and 14.4 min nanoLC gradient.
7g: Superimposed PSCs, UV and MS data for *Crotalus atrox, Echis ocellatus, Naja nigricollis* and *Naja pallida* analysed under optimised HT Venomics conditions.
7h: Superimposed PSCs and UV data for *Bungarus multicinctus*, *Daboia russelii*, *Naja naja, Ophiophagus hannah, Naja mossambica and Bothrops asper* analysed under optimised HT Venomics conditions.

Suppl. Table 1. Snake venoms used in this study with information on their origin, and analytical information

Species	Family	Origin	Continent	LC Acidifier used for analysis	Database	MS Data available
Echis ocellatus	Viperidae	Nigeria	Africa	Both FA and TFA	Uniprot+Transcriptomic	Yes
Daboia russelii	"	Sri Lanka	Asia	TFA	Uniprot	No
Calloselasma rhodostoma		captive bred	Asia	Both FA and TFA	Uniprot+Transcriptomic	Yes
Crotalus atrox	"	USA	N. America	TFA	Uniprot	Yes
Bothrops asper	"	Costa Rica	C. America	TFA	Uniprot	No
Naja nigricollis	Elapidae	Togo	Africa	TFA	Uniprot+Transcriptomic	Yes
Naja mossambica	"	Historical collection	Africa	TFA	Uniprot+Transcriptomic	No
Naja pallida	"	Tanzania	Africa	Both FA and TFA	Uniprot+Transcriptomic	Yes
Naja naja	"	captive bred	Asia	TFA	Uniprot+Transcriptomic	No
Ophiophagus hannah	"	Thailand	Asia	TFA	Uniprot	No
Bungarus multicinctus	"	Historical collection	Asia	TFA	Uniprot	No

Section 1. *SI Evaluation of robotically operated tryptic digestion and Mascot database searching complementarity of Uniprot versus Transcriptomic.*

Whereas in our previous studies involving venom proteomics the tryptic digestions were performed in a manual fashion, in this study most of the workflow was automated. For the tryptic digestions, this was achieved by performing each of the steps of the digestion procedure with a Thermo Scientific™ Multidrop™ 384 Reagent Dispenser. Due to the large dead volume (±7 mL) of the aspiration tubes from the Multidrop, a relatively large amount of the used solvents is wasted compared to when they are manually pipetted. A large part of this dead volume can however be reused as the Multidrop has an option to empty the aspiration tubes by back flushing. In case of the reduction buffer and alkylation agent the amount wasted this way is cost wise negligible. Unfortunately, this is not the case for trypsin as the manufacturer states that once trypsin is reconstituted in a solvent around pH 8.0 it will lose its enzymatic activity over time and thus cannot be reused. Therefore, the trypsin solution can alternatively be pipetted using a multichannel pipet. On the other hand, when many plates are prepared for tryptic digestion simultaneously, in our case up to 10 plates were digested in one go, the relative loss of expensive trypsin solution is drastically reduced when also using the Thermo multidrop for pipetting the trypsin solution. It has to be noted in this regard that a study by Giansanti *et al* states that they did not experience a loss in enzymatic activity when the trypsin was reconstituted in 50 mM NH4HCO3, pH 8 when stored long term at -80.

To compare results of performing the tryptic digestions directly on the well plates after nanofractionation with performing them after transfer to Eppendorf tubes in a manual fashion, venom of the Malayan pit viper (*Calloselasma rhodostoma*) was analysed by the HT venomics approach in two different ways. First, analysis was done according to the in this study described procedure. Second, the same procedure was followed except for the tryptic digestions, which were performed manually according to Slagboom *et al*. Figure 1 shows typical results obtained with the HT venomics approach. In manuscript Figure 1, superimposed chromatograms are shown of the LC-UV data, of the Total Ion Chromatogram (TIC) and relevant eXtracted Ion Chromatograms (XICs) from the MS data, and of the Protein Score Chromatograms (PSCs) resulting from Mascot database searching using a Uniprot database containing only accessions from Serpentes. For all venoms analysed in this study, Uniprot database searching was performed and when a venom gland transcriptome database was available, this database was searched in addition. Both these searches give complementary results. The transcriptomics database allows to search the transcriptome of the venom gland of the same species of which the venom proteome is under analysis thereby resulting in the

most accurate protein matches. Especially if the venom gland transcriptome is derived from venom glands of the same species of which the venom is analysed, which is the case in our study for *Echis ocellatus, Calloselasma rhodostoma, Naja pallida, Naja naja, Naja nigricollis and Naja mossambica* of the venoms analysed. It on the other hand does only provide non-informative protein identified numbers. Uniprot on the other hand only will retrieve the toxins of which information is available in the database. Also, it will retrieve results of venom toxins of similar species when peptides were found in the tryptic digests that are identical to peptide sequences in these similar toxins from other species. This can be a disadvantage, but can also be an advantage in terms of providing information on similar toxins in different species. Importantly, Uniprot also conveniently provides valuable information on toxin families and toxin names, and on publications describing the toxins retrieved. Also, additional information such as toxin pathologies and/or *in vitro* assay bioactivities can this way easily be found, if available in published work referred to by Mascot via the Uniprot database.

In order to make this procedure high-throughput all the pipetting steps in the tryptic digestion were automated. To compare this to the standard manual procedure a comparison experiment was performed in which venom of *Calloselasme rhodostoma* was subjected to manual and automated tryptic digestion and subsequently analysed with nanoLC-MS/MS. When comparing PSCs from both digestion procedures, highly similar results were obtained. The same toxins were found and similar protein scores and sequence coverages were obtained. Therefore, the automated tryptic digestion procedure used directly on the well plates in which separated venom toxins were collected was shown to be successful and used further in this study.

Section 2. SI nanoLC-MS/MS gradients

NanoLC-MS/MS run times in proteomics studies are often time consuming and can take up to 60-120 min per run. Also, in typical venomics research these run times are applied. In this study, snake venoms were fractionated in high resolution on 384 well plates for subsequent proteomic analysis on all relevant collected fractions. When subjected to standard nanoLC separation run times, the full proteomic analysis of one snake venom could take more than a month solely for the LC runs due to each venom yielding hundreds of fractions when subjected to our methodology. Since the high-resolution fractionation of the nanofractionation setup, as previously described, limits the average number of toxins collected in each well significantly and thereby decreases sample complexity, it allows for a drastic reduction of LC runtime. In our study, we managed to reduce LC runtimes down to 14.4 min per run. This translates to 100 nanoLC runs per day. Due to the dead volume delays in our nanoLC setup and dead

volume in the column, further reduction in runtime would compromise the gradient part of the nanoLC runs. With 14.4 min runtimes all tryptic peptides eluted between 7.2 and 14.2 min. Still, decreasing the analytical separation run time of course does have a substantial effect on the chromatography and ability of the MS to measure all the peptides despite the limited complexity of the samples. By having shorter runtimes, the acquisition speed of a mass spectrometer can become a bottleneck. To test the impact of significantly reducing the nanoLC runtimes, the contents of several wells of the HT venomics analysis of the *Calloselasma rhodostoma* venom described in the previous section were used for re-measuring with a 60 min nanoLC gradient. Typical results from measuring wells in quadruplicate using both the short and the long gradient are given for well N5 in SI Table 3. For this particular sample it was determined that the protein scores found in the 14.4 min runs were on average 38% lower than the 60 min runs. For the sequence coverages it was found that on average 19% less sequence coverage was found for the toxins with the 14.4 min runs as compared to the 60 min runs. However, the same proteins were retrieved from in the 14.4 min analyses and from the 60 min analyses. The same trend was observed when analysing other wells using both gradients. In practically all cases the same venom toxins were retrieved for which lower protein scores were found and lower sequence coverages. These results showed that the fast gradient can be applied successfully in the HT venomics methodology. It has to be noted that in our study we used a maXis QTOF. Evidently, when using a state-of-the-art mass spectrometer dedicated for proteomics research higher protein scores and sequence coverages are to be expected.

Section 3. SI LC-MS with nanofractionation

Often when performing LC separations in combination with positive ElectroSpray Ionisation mass spectrometry, formic acid (FA) is used in the eluents as acidifier. Also, this is true for LC separations of venoms. When separating venom toxins in crude venoms, especially for the larger toxins, often significant to severe peak broadening occurs. Much higher separation efficiencies can be obtained by using TriFluoroAcetic Acid (TFA) instead of FA in the eluents. TFA however is well known to be incompatible with MS analysis. We hypothesised that this is only partly true and depends on the TFA concentration and flowrate going to MS. Since we use a flow split after which only 10% of the original LC flowrate goes to MS, we tested compatibility of MS analysis using TFA as eluent acidifier. Testing compatibility of LC with MS when having 0.1% FA or 0.1% TFA in the LC eluent at both low (~50µL/min) and high (~450 µL/min) flowrates going to MS resulted in similar sensitivities obtained in case of both low and high flowrates going to MS for FA and only for low flowrates for TFA. For these TFA experiments, the toxins measured did have slightly different charge state envelopes, which

however did not give other accurate masses (due to possible ion clustering) as those found for the FA experiments. When testing TFA at the high flowrate, gradually the sensitivity decreased during the first LC-MS run, and no venom toxins could be detected anymore in the second analysis run. Figures showing actual data can be found in the SI section 7f. These results showed that in our setup, both FA and TFA can be used.

For testing the compatibility of LC with MS when having FA or TFA in the LC eluent at both low (~50µL/min) and high (~450 µL/min) flowrates going to MS, we first tested FA in the eluent and did LC-MS runs as described for the HT venomics procedure. In this procedure, 10% of the eluent flow was split to MS. We then swapped the flow split and directed the 90% fraction of the eluent to MS. Both setups resulted in reproducibly obtaining good quality LC-MS data on analysed venoms. The sensitivities obtained were similar for both setups even though the large difference between the two flowrates going to MS tested. (typical data on the 10% of the flow going to MS is shown in Figure 2), Next, TFA was tested using the larger fraction after the flow split going to MS. During the first analysis run, gradually the sensitivity decreased, and no venom toxins could be detected anymore in the second analysis run (data not shown). Subsequently infusing standard tune solution (3 microlitre/min) did not give a measurable signal in MS. Only after several hours of infusing the tune solution, the signal returned to normal. When then again running the same LC-MS analyses runs, the similar results were obtained (i.e. graduate sensitivity drop until no measurable signal anymore after the first LCMS run). Then, the eluent flow after the flow splitter was swapped again so the lower eluent flow rate (i.e. 50 microliter/min) fraction was directed to MS. When performing LC-MS measurements using these conditions, it was found that sensitive and reproducible results were obtained. Sequences of LC-MS runs could be operated for days without sensitivity losses. Sensitivities found were similar to those obtained when having FA in the eluents as acidifier. The same toxin accurate masses were retrieved. (typical results obtained are given in section 7f). Using these conditions, the clear advantages of TFA, namely high resolution venom toxin separations, could be combined with acquiring sensitive and reproducible LC-MS data. Results obtained this way for *Calloselasma rhodostoma, Echis ocellatus* and *Naja pallida* venom are given in section 7g. The increase in separation resolution as compared to using FA as acidifier is clearly seen from the LCMS data in section 7e.

Section 4. SI Comparison of proteomics results after LC-MS runs with FA or TFA as acidifier

From the proteomics data obtained for venoms of the three snake species that were analysed after separation with FA or TFA as acidifier in the LC eluents, detailed information will now be given:

For *Calloselasma rhodostoma* the number of Uniprot entries present is 17 from which 9 were found by using the method used by Slagboom et al. When using the HT venomics approach developed in this study, with the same LC-MS and nanofractionation parameters, we found 10 of the 17 proteins back. When having TFA as acidifier instead of FA, 13 out of 17 Uniprot entries were found. From these findings together with those of the previously described individual analyses of the contents of wells of *Calloselasma rhodostoma* in the nanoLC-MS/MS section it can be concluded that the shortened nanoLC-MS/MS method does not affect the number of found proteins in a negative manner. However, one must consider that when attempting to analyse low abundant proteins with the method developed in this study, they might not be detected. This was in fact shown when using the species-specific transcriptome database for database searching. For the Slagboom *et al* method with FA, 25 out of 37 protein IDs were found compared to 22 out of 37 for the method developed in this study. For the method developed in this study using TFA instead of FA however, 27 out of 37 protein IDs were found. It was anticipated that the improved separation resolution when using TFA instead of FA resulted in a higher number of protein IDs and therefore makes it more suitable for the HT venomics approach, and probably for other venomics approaches as well.

For *Echis ocellatus* the analyses were done in exactly the same fashion as described for *Calloselasma rhodostoma*. For the method described by Slagboom *et al*, 12 out of 22 Uniprot protein IDs were found compared to 14 out of 22 for the method described in this study using FA. For TFA, 13 out of 22 Uniprot protein IDs were found. This (slight) difference in protein IDs is opposite of what was observed for *Calloselasma rhodostoma* and could be accounted for by the fact that these measurements were performed on different days. Sensitivity of the mass spectrometer can differ between periods of time and this could well be the reason. With FA, 29 out of 32 IDs were found for the species-specific database both for the Slagboom *et al* data and the data acquired in this study. When using TFA, 31 out of 32 protein IDs where found from the species-specific database.

For *Naja pallida* only data was acquired using the methodology developed in this study as this venom was not included in the study of Slagboom *et al*. Only few proteins were available in the Uniprot database which reduced the significance of database searching using this database. Nevertheless, 3 out of 3 protein IDs were found for both FA and TFA when using the Uniprot database. When using the species-specific database 20 out of 26 protein IDs were found for FA and 22 out of 26 for TFA.

Section 5. SI Resolution of fractionation and nanoLC-MS/MS analysis time per venom

A straightforward manner of reducing the HT venomics workflow time required to process one venom by about half is to double the fractionation time resolution (i.e. from 6 sec to 12 sec). This way also more of a venom toxin will be collected in one well thereby anticipating that eventual proteomics results will be better. For this, we investigate the fractionation resolution with *Calloselasma rhodostoma*, *Echis ocellatus* and *Naja pallida* venoms and found that the lower fractionation resolution (i.e. 12 sec) did not compromise the resolution of the PSCs as very similar results were achieved. Also, the same number of venom toxins were retrieved, however with approximately 1.6x higher protein scores for the 12s fractionation resolution (more toxin was collected per fraction) and with similar sequence coverages (deviation 10%). Figures are shown in section 7d.

Using too low fractionation resolutions on the other hand will cause separated venom toxins to be collected in the same well again thereby compromising the achieved separation resolution. This is especially the case when TFA is used in the eluents as acidifier as high separation resolutions are achieved using TFA. Another issue with lowering the fractionation resolution is that at some point the eluent volume to be collected per well exceeds the well volume of the 384-wel plates used. Lowering the LC separation flow rate can counteract this partly, but in turn also will affect the separation. We tested a fractionation resolution of 12 sec with the venom of *Calloselasma rhodostoma*, *Echis ocellatus* and *Naja pallida.* This indeed reduced the total HT venomics time to analyse and process a venom by half and resulted in less than two days for the complete analysis. To evaluate if the reduced fractionation resolution compromised the peak resolution in the PSCs, for the venoms analysed this was compared in section 7d. When comparing the results from the 12 sec fractionation time with those of the 6 sec fractionation resolution it was concluded that the lower fractionation resolution did not compromise the resolution of the PSCs as very similar results were achieved. Also, the same number of venom toxins were retrieved, however with approximately 1.6x higher protein scores for the 12s fractionation resolution but similar sequence coverages were obtained (deviation 10%).

Section 6. SI Conclusion

In this study high-throughput venomics is introduced. The approach allows rapid and efficient almost automated profiling of venoms to obtain the respective venom proteomes. These proteomes are conveniently plotted as so-called protein score chromatograms (PSCs) which allow direct correlation with parallel obtained LC-MS data and with LC-UV data of venoms. Automation of data processing and data plotting into easy interpretable formats was done by in-house made scripts of which one script enabled peak integration of peaks in the PSCs for semi-quantitation purposes. By comparing multiple PSC peaks in one PSC with peak shape and retention time matching XICs from the parallel acquired LC-MS data, PTMs on venom toxins can be investigated further.

The HT venomics procedure implemented parts of our previously developed nanofractionation methodology and combined this with a rapid and automated tryptic digestion protocol for the proteomics part. By automation of the proteomics protocol, by reducing the nanoLC-MS/MS run times drastically, and by automating the data processing by using scripts it became possible to do a full proteomic analysis of a single snake venom in less than two days when analysing multiple venoms in a sequence and using a 12 sec fractionation resolution. During one working day, approximately 8 to 10 venoms can be nanofractionated on well plates using the current LC-MS gradient. When 12 sec fractionations are used, two venoms can be nanofractionated on one well plate. For 6 sec fractions, one well plate is fully used for one venom. The vacuum-centrifuge evaporator has a capacity of 12 well plates. The next day, the dried well plates can be stored or prepared for tryptic digestion. As this procedure is performed robotically, the number of plates to be processed in one day is practically unlimited. We usually processed between 6 to 12 plates in one day. The following day, quenching solution was robotically pipetted to all wells and three plates could directly be transferred to the autosampler of the nanoLC-MS/MS system, while the other plates were stored at -20 °C until analysis. The nanoLC-MS/MS system measured 100 tryptic digests per day and after all measurements of a snake venom were completed, the acquired data was automatically processed through the scripts described in this study.

Using FA or TFA as eluent acidifier for the LC-MS venom separations and using the Uniprot and/or species-specific transcriptome database Mascot searches were investigated with the venoms of *Calloselasma rhodostoma, Echis ocellatus* and *Naja pallida*. It was concluded that TFA could be used for the LC-MS analyses when splitting 10% of the LC eluent post-column to MS, which is standard to the HT venomics procedure. This resulted in much higher separation resolutions then using FA. For the database searches also on average more

protein IDs were retrieved when TFA was used for separation of the venom toxins. Uniprot and species-specific databases gave complementary results and the species-specific databases retrieved more venom toxin IDs. Also, these IDs represent exact matches as venom from the same species is searched on its respective transcriptome database.

Reducing the nanoLC-MS/MS run times came with the price of loss in nanoLC separation and in MS data acquisition time per tryptic peptide. As a result, lower protein scores and sequence coverages were found. Nevertheless, still the same number of protein IDs were retrieved with the new fast 14.4 min nanoLC-MS/MS method as compared to the standard 60 min long gradient. From this it was concluded that the new nanoLC method reduced analysis time drastically and still retains the necessary sensitivity for the proteomic analyses.

An estimation could be made on the relative abundance of toxins and of toxin families in the venoms analysed. However, for the current HT venomics approach its quantitative abilities remain relative. In order to achieve absolute quantitation as demonstrated by Calderón-Celis et al and Calvete et al additional features need to be implemented such as standards and/or labeling procedures. Nevertheless, the here presented venomics workflow is orders of magnitude faster than any other venomics approach described in literature to date.

Section 7. *SI Figures & Tables*

7a: Pie charts of toxins found for each HT Venomics analysis

***Bothrops asper* TFA Uniprot**

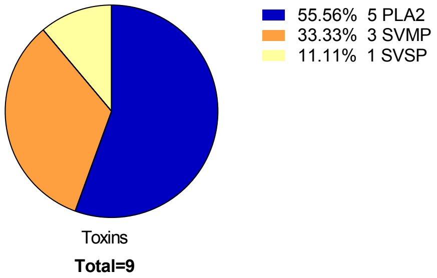

***Bungarus multicinctus* TFA Uniprot**

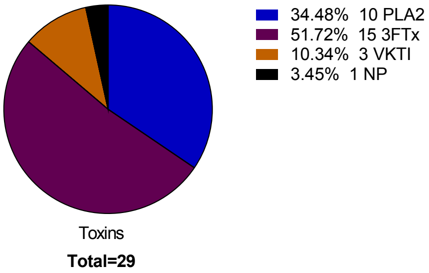

***Crotalus atrox* TFA Uniprot**

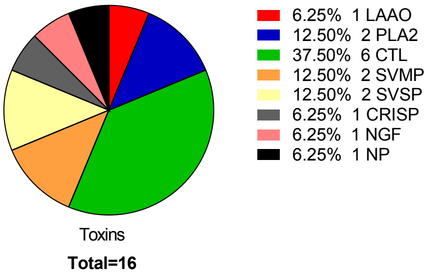

***Daboia russelli* TFA Uniprot**

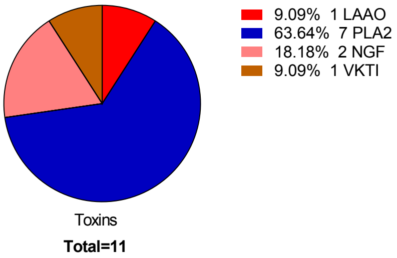

***Echis ocellatus* TFA Half transcriptomic**

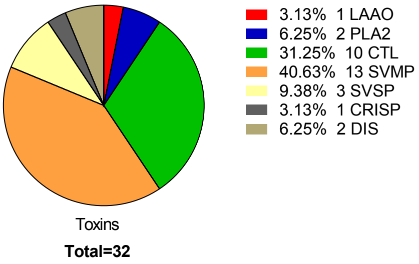

***Echis ocellatus* TFA Half Uniprot**

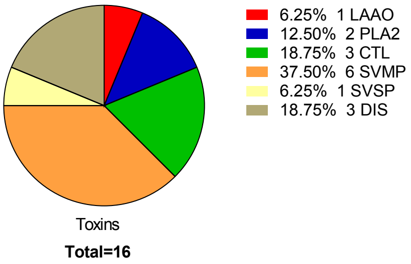

***Naja naja* TFA Uniprot**

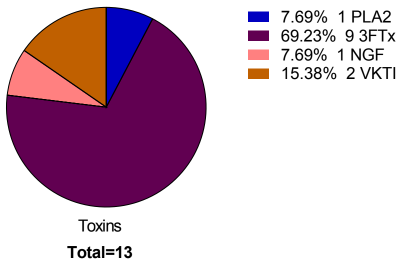

***Naja naja* TFA transcriptomic**

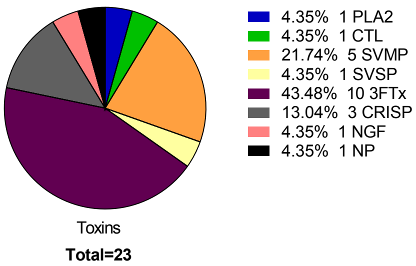

***Naja nigricolis* TFA Uniprot**

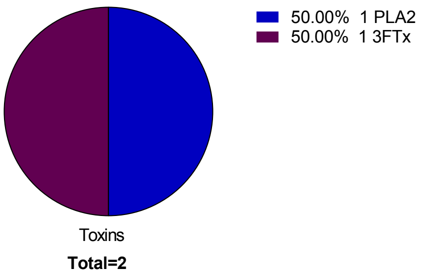

***Naja nigricolis* TFA transcriptomic**

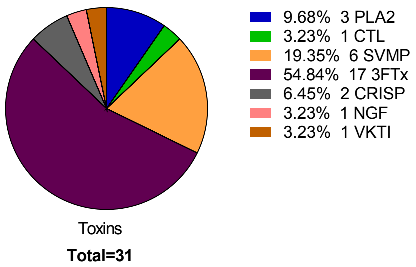

***Naja pallida* TFA Uniprot**

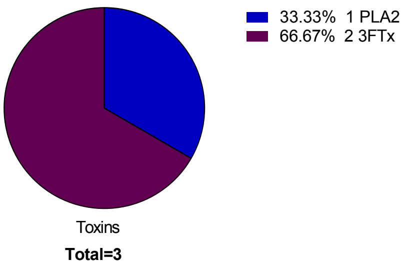

***Naja pallida* TFA transcriptomic**

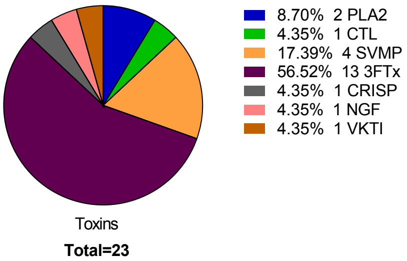

Naja mossambica* TFA *Uniprot

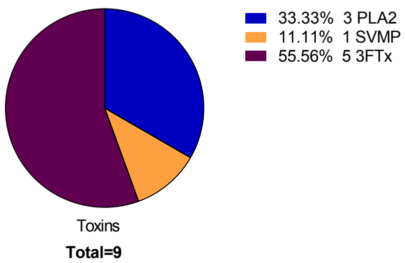

***Naja mossambica* TFA Transcriptomic**

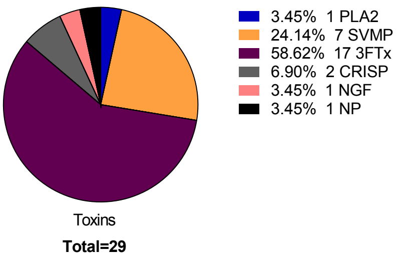

***Ophiophagus hannah* TFA Uniprot**

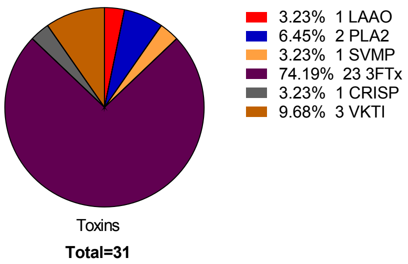

***Calloselasma rhodostoma* TFA Uniprot**

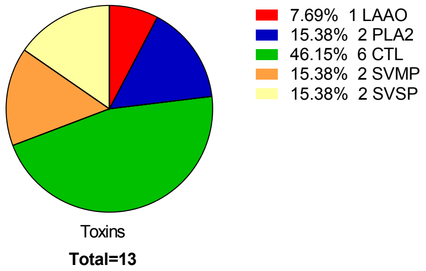

***Calloselasma rhodostoma* TFA transcriptomic**

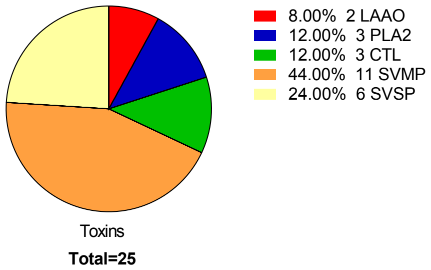

***Calloselasma rhodostoma* FA manual Uniprot**

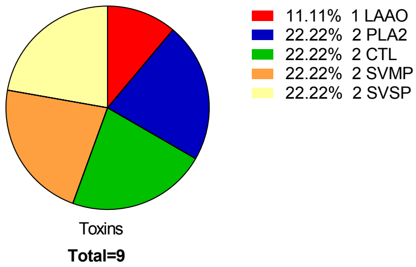

***Calloselasma rhodostoma* FA manual transcriptomic**

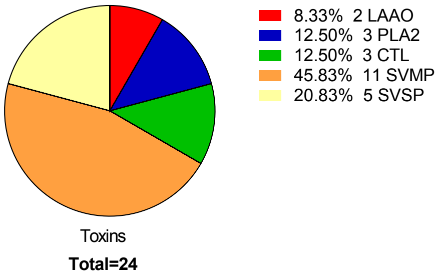

***Calloselasma rhodostoma* FA automatic Uniprot**

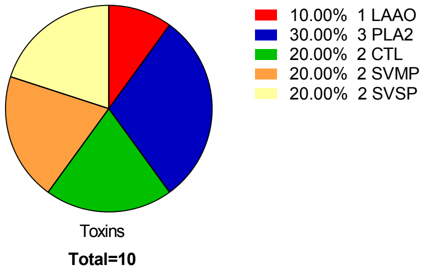

***Calloselasma rhodostoma* FA automatic transcriptomic**

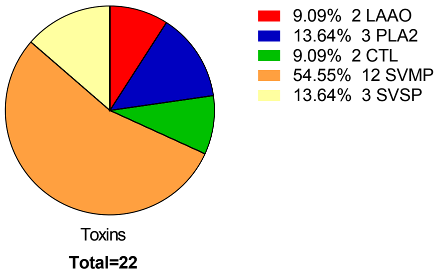

***Calloselasma rhodostoma* TFA automatic Uniprot**

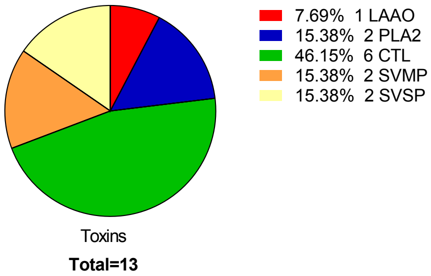

***Calloselasma rhodostoma* TFA automatic Transcriptomic**

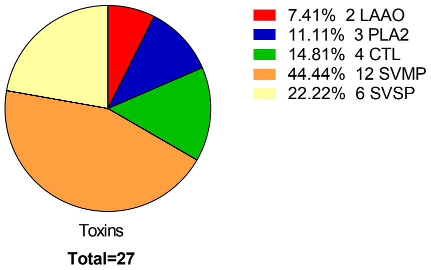

***Echis ocellatus* FA automatic Uniprot**

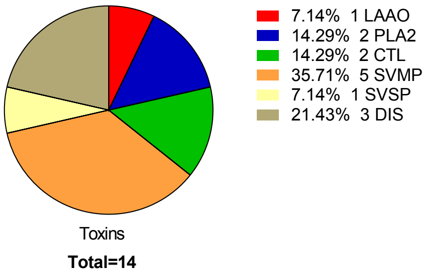

***Echis ocellatus* FA automatic transcriptomic**

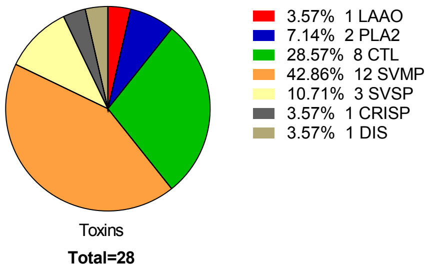

***Echis ocellatus* TFA automatic Uniprot**

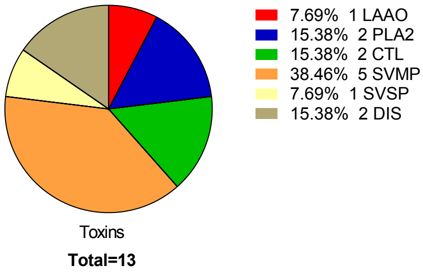

***Echis ocellatus* TFA automatic transcriptomic**

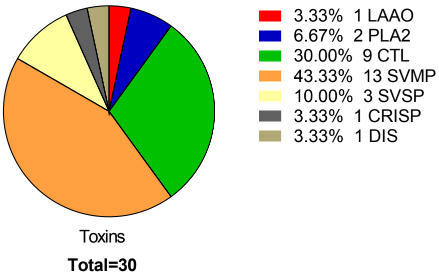

***Echis ocellatus* FA manual Uniprot**

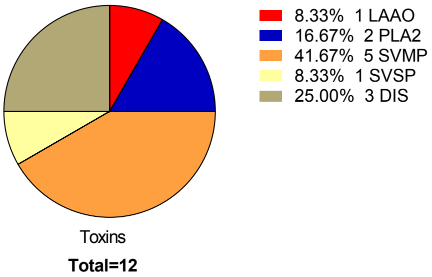

***Echis ocellatus* FA manual transcriptomic**

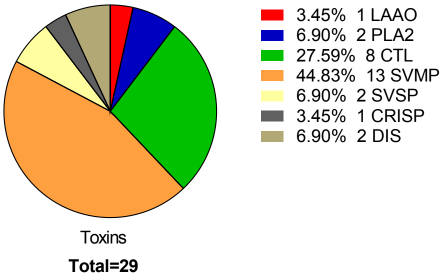

***Naja pallida* FA automatic Uniprot**

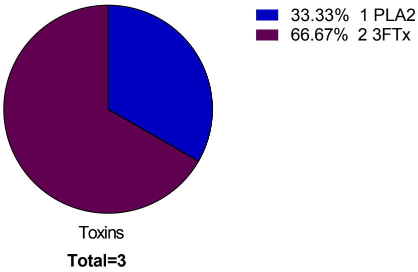

***Naja pallida* FA automatic transcriptomic**

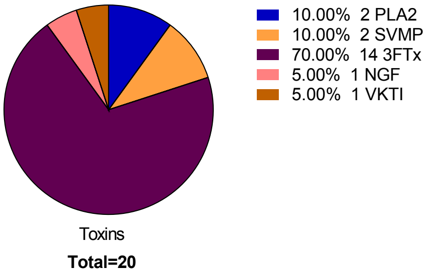

***Naja pallida* TFA automatic Uniprot**

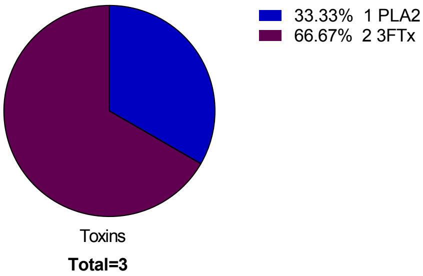

***Naja pallida* TFA automatic transcriptomic**

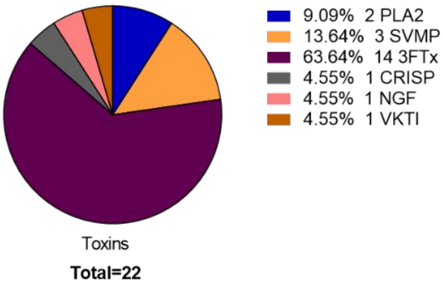

***Naja nigricollis* 12s Uniprot**

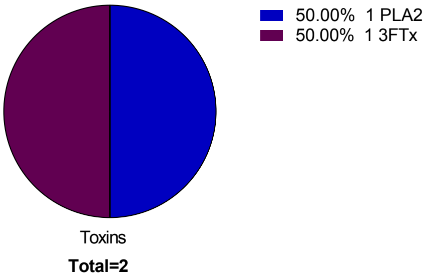

***Naja nigricollis* 12s Transcriptomic**

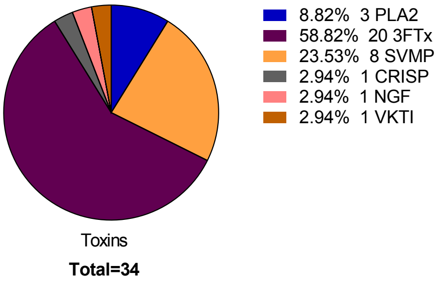

7b: Protein Score Chromatograms of *Naja Nigricollis* 12 sec TFA Mascot searches

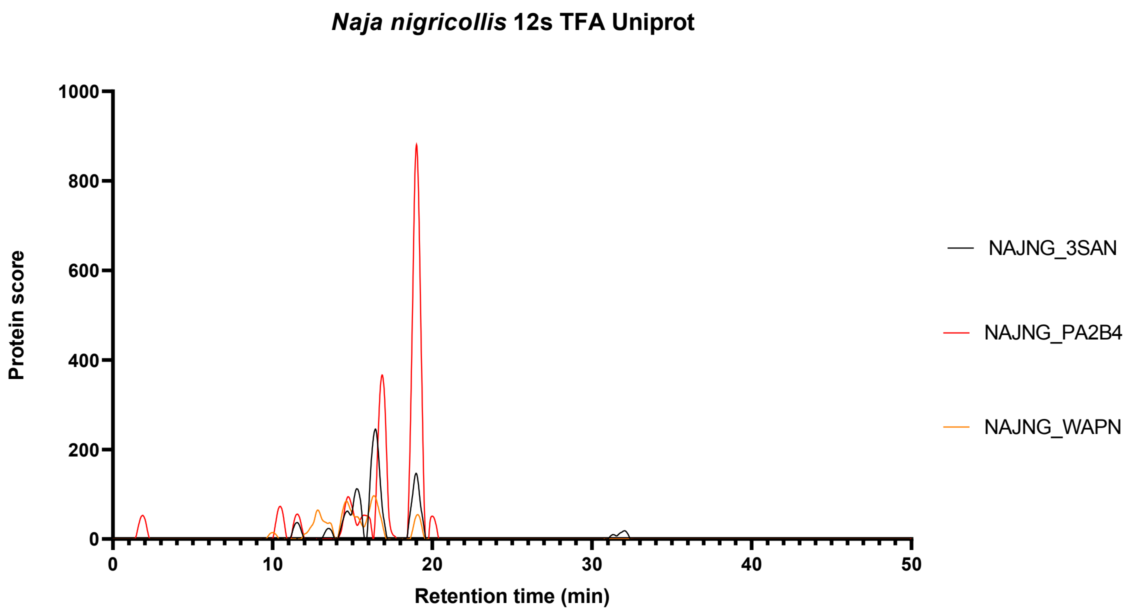

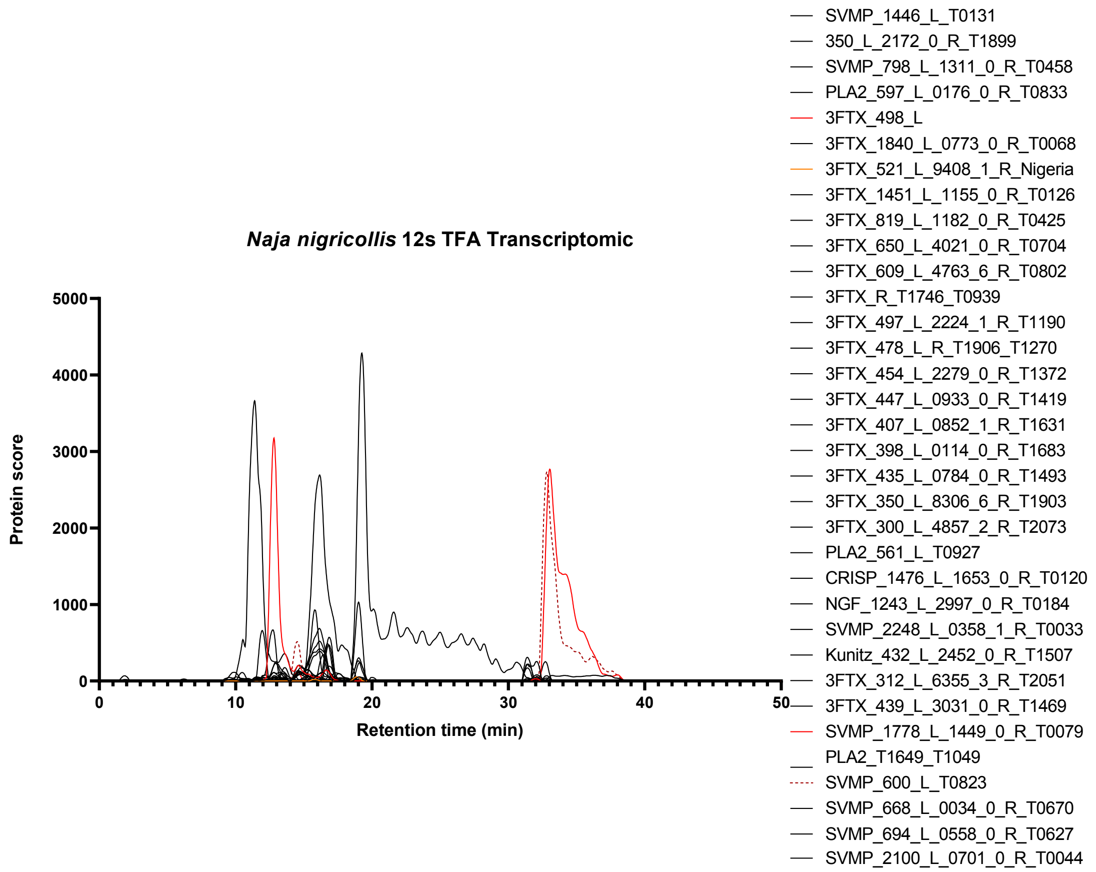
Naja nigricollis 12s TFA Transcriptomic
Protein score
5000
4000
3000
2000
1000
0
0
10
20
30
40
50
Retention time (min)
SVMP_1446_L_T0131
350_L_2172_0_R_T1899
SVMP_798_L_1311_0_R_T0458
PLA2_597_L_0176_0_R_T0833
3FTX_498_L
3FTX_1840_L_0773_0_R_T0068
3FTX_521_L_9408_1_R_Nigeria
3FTX_1451_L_1155_0_R_T0126
3FTX_819_L_1182_0_R_T0425
3FTX_650_L_4021_0_R_T0704
3FTX_609_L_4763_6_R_T0802
3FTX_R_T1746_T0939
3FTX_497_L_2224_1_R_T1190
3FTX_478_L_R_T1906_T1270
3FTX_454_L_2279_0_R_T1372
3FTX_447_L_0933_0_R_T1419
3FTX_407_L_0852_1_R_T1631
3FTX_398_L_0114_0_R_T1683
3FTX_435_L_0784_0_R_T1493
3FTX_350_L_8306_6_R_T1903
3FTX_300_L_4857_2_R_T2073
PLA2_561_L_T0927
CRISP_1476_L_1653_0_R_T0120
NGF_1243_L_2997_0_R_T0184
SVMP_2248_L_0358_1_R_T0033
Kunitz_432_L_2452_0_R_T1507
3FTX_312_L_6355_3_R_T2051
3FTX_439_L_3031_0_R_T1469
SVMP_1778_L_1449_0_R_T0079
PLA2_T1649_T1049
SVMP_600_L_T0823
SVMP_668_L_0034_0_R_T0670
SVMP_694_L_0558_0_R_T0627
SVMP_2100_L_0701_0_R_T0044

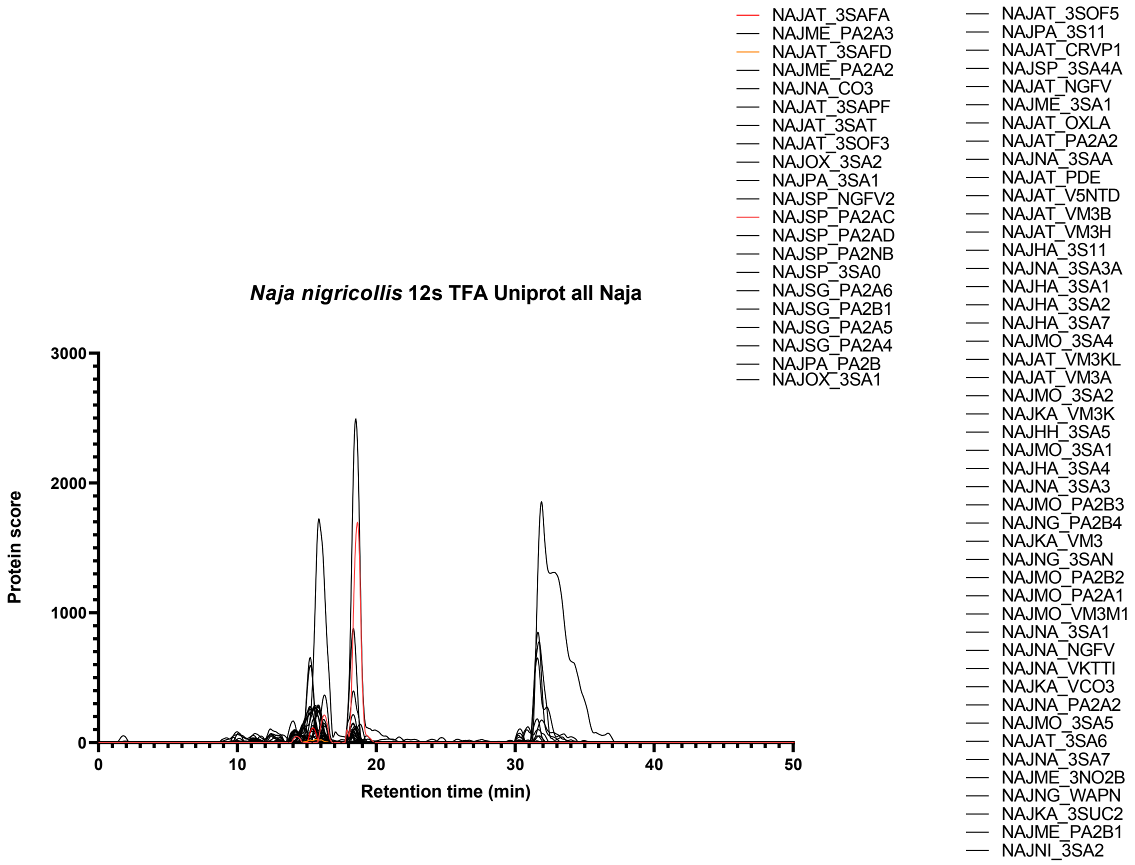
Naja nigricollis 12s TFA Uniprot all Naja
Protein score
3000
2000
1000
0
0
10
20
30
40
50
Retention time (min)
NAJAT_3SAFA
NAJME_PA2A3
NAJAT_3SAFD
NAJME_PA2A2
NAJNA_CO3
NAJAT_3SAPF
NAJAT_3SAT
NAJAT_3SOF3
NAJOX_3SA2
NAJPA_3SA1
NAJSP_NGFV2
NAJSP_PA2AC
NAJSP_PA2AD
NAJSP_PA2NB
NAJSP_3SA0
NAJSG_PA2A6
NAJSG_PA2B1
NAJSG_PA2A5
NAJSG_PA2A4
NAJPA_PA2B
NAJOX_3SA1
NAJAT_3SOF5
NAJPA_3S11
NAJAT_CRVP1
NAJSP_3SA4A
NAJAT_NGFV
NAJME_3SA1
NAJAT_OXLA
NAJAT_PA2A2
NAJNA_3SAA
NAJAT_PDE
NAJAT_V5NTD
NAJAT_VM3B
NAJAT_VM3H
NAJHA_3S11
NAJNA_3SA3A
NAJHA_3SA1
NAJHA_3SA2
NAJHA_3SA7
NAJMO_3SA4
NAJAT_VM3KL
NAJAT_VM3A
NAJMO_3SA2
NAJKA_VM3K
NAJHH_3SA5
NAJMO_3SA1
NAJHA_3SA4
NAJNA_3SA3
NAJMO_PA2B3
NAJNG_PA2B4
NAJKA_VM3
NAJNG_3SAN
NAJMO_PA2B2
NAJMO_PA2A1
NAJMO_VM3M1
NAJNA_3SA1
NAJNA_NGFV
NAJNA_VKTTI
NAJKA_VCO3
NAJNA_PA2A2
NAJMO_3SA5
NAJAT_3SA6
NAJNA_3SA7
NAJME_3NO2B
NAJNG_WAPN
NAJKA_3SUC2
NAJME_PA2B1
NAJNI_3SA2

7c: Comparison PSC peak area and protein score summing

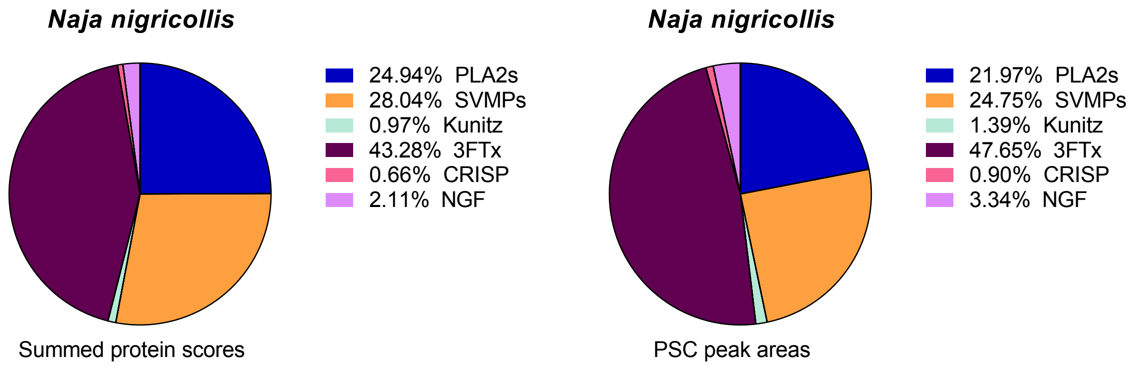

For semi-quantitation of the found venom toxins either the PSC peak areas could be calculated or the total amount of protein scores could be summed. A comparison between these two approaches was made to determine if there was a significant difference between the two. The results show that the deviation was minimal and the two approaches yield similar results.

7d: Comparison 6sec and 12sec fractionation for 3 species

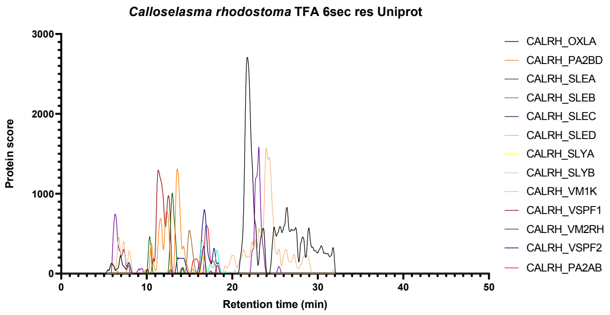

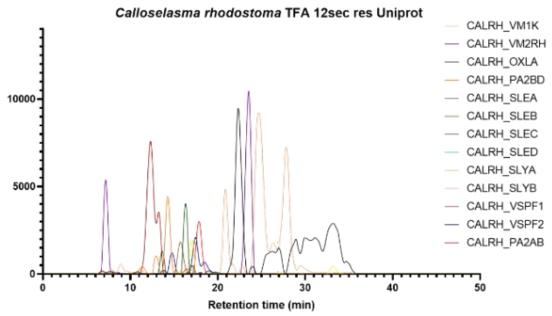

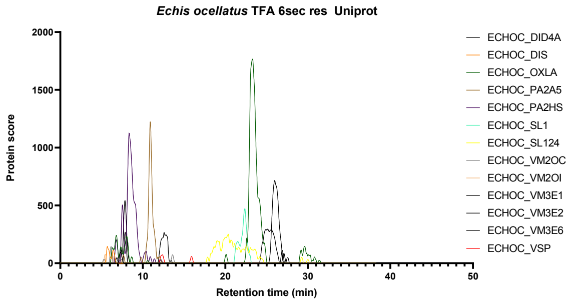

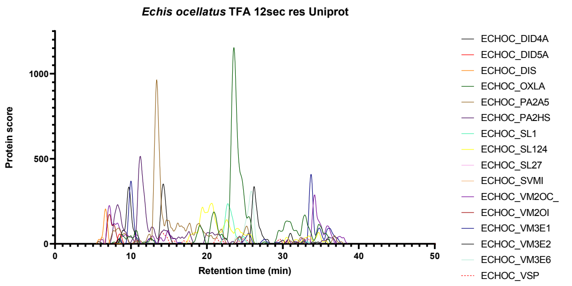

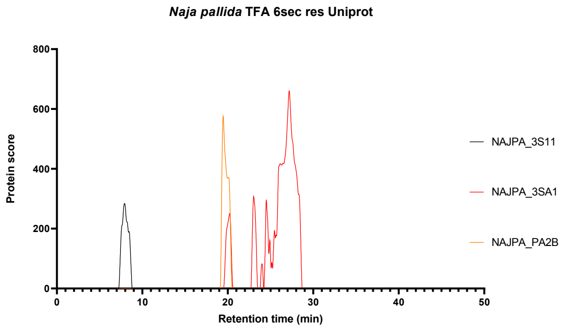

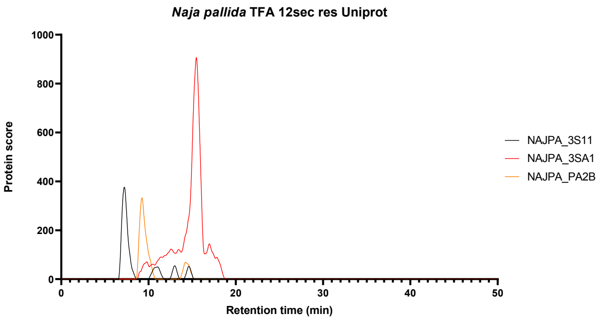

A comparison was made between the proteomic results after 6 sec and 12 sec fractionation of snake venoms. Despite collecting double the amount of volume per well this did not have an negative effect on the PSC resolution of the found venom toxins. This is due to the fact that venom toxins have a longer elution time (± 30-60 sec) than 12 sec. Therefore, the 12 sec fractionation resolution resulted in a decrease of 50% of the nanoLC-MS/MS analysis time while maintaining the same PSC resolution.

7e: Comparison between FA and TFA as acidifier in LC-UV-MS separation.

LC-UV Chromatograms

Calloselasma rhodostoma

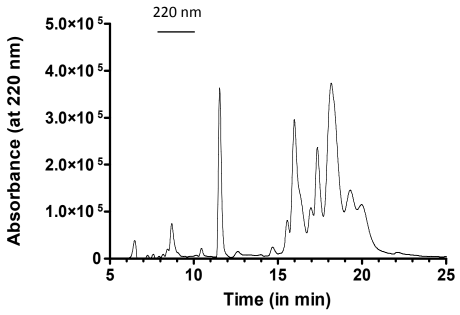

Calloselasma rhodostoma

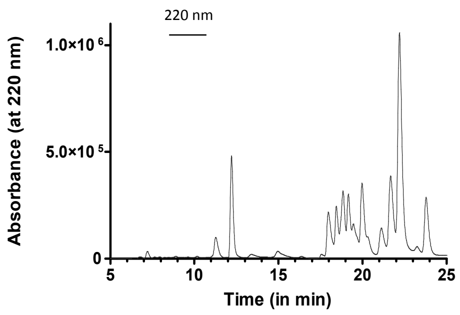

Naja pallida (FA)

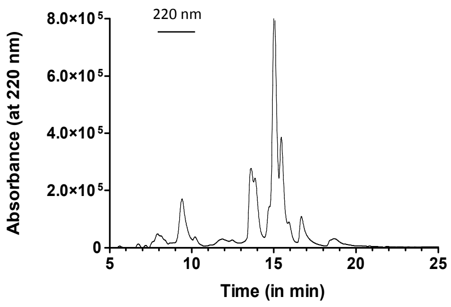

Naja pallida (TFA)

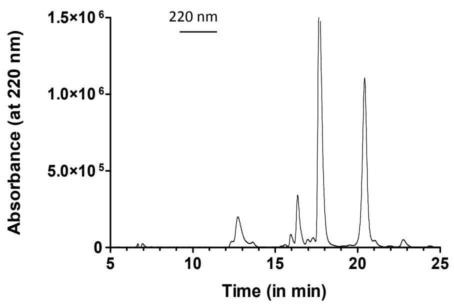

FA and TFA were compared when used as acidifiers in LC-UV separation of snake venoms. As seen in the graphs above, using TFA results in a better separation between the venom toxins than when using FA. This is in line with what is known in literature. Therefore, TFA was used as acidifier in all of the following venom separations.

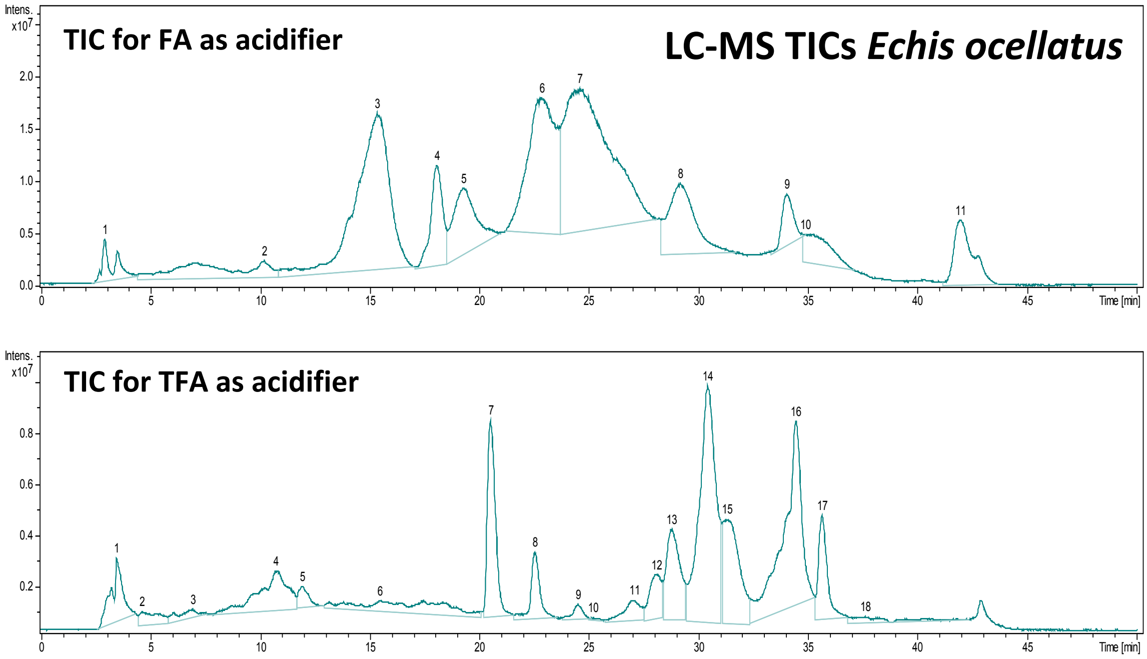

FA and TFA were compared when used as acidifiers in LC-MS separation of snake venoms. As seen in the graphs above, using TFA results in a better separation between the venom toxins than when using FA. This is in line with what is known in literature. However, FA is usually used when doing MS measurements due to TFA causing ion suppression. In our set-up only 10% of the total flow (0,5 mL/min) goes to the MS, which negates the suppressing effect of TFA. Therefore, TFA was used as acidifier in all of the following venom separations.

7f: Charge states FA and TFA for *Calloselasma rhodestoma* toxins.

Suppl.Table 2. Comparison charge states of venom toxins when using FA and TFA.

	FA		
Toxin	m/z	charge(+)	mass
1	887.428	1	886.421
2	2305.028	6	13815.09
3	2774.006	5	13855.95
4	2533.751	12	30404.18
5	2470.151	12	29620.46
	TFA		
Toxin	m/z	charge(+)	mass
1	887.424	1	866.417
2	2305.018	6	13815.03
3	2773.974	5	13855.79
4	2534.072	12	30389.71
5	2470.799	12	29637.5

Comparison charge states found for a selection of toxins from *Calloselasma rhodostoma* when measured with FA and TFA. When comparing toxins found when analysing the venom with FA and TFA, it is evident that the same charge states are observed. This shows that TFA does not have an negative effect on venom toxin charge states.

Comparison charge states with 0 and 200 isCID.

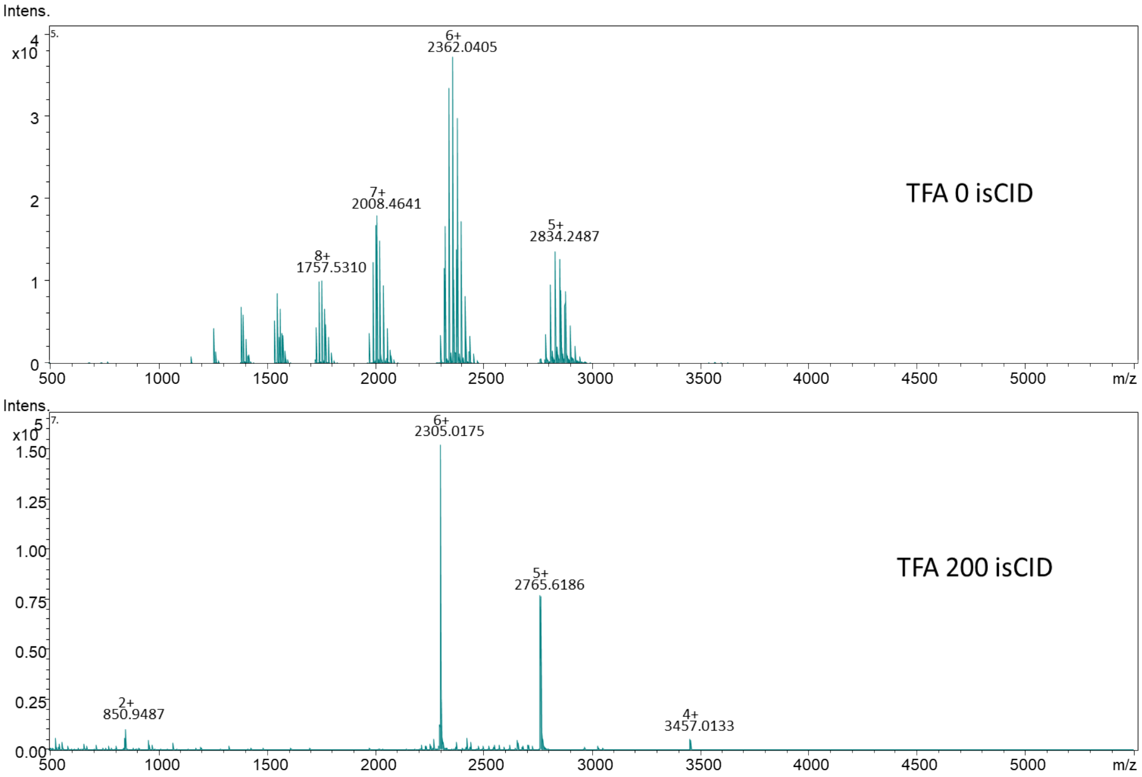

When using TFA in MS measurements the forming of TFA clusters is inevitable. To reduce this isCID is used to “shoot” off these clusters by means of collision. When comparing 0 and 200 isCID a significant decrease is observed in TFA clusters, which results in the remaining actual toxin m/z values.

Effect of TFA on MS performance after infusion of a high LC flow.

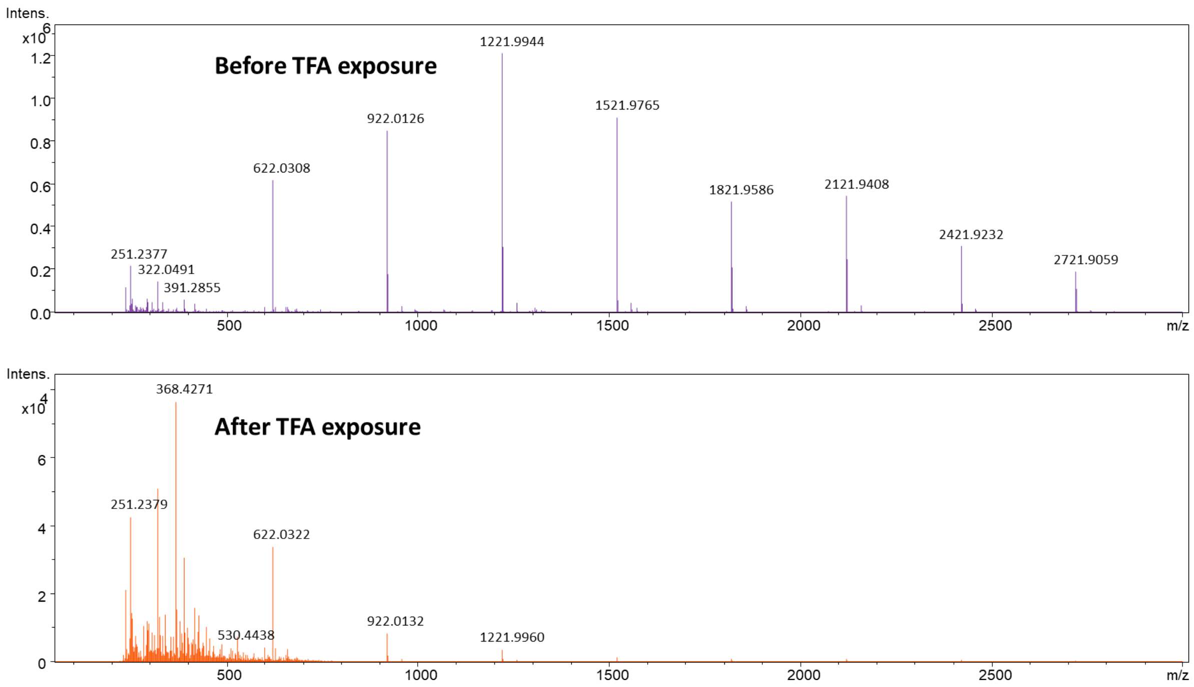

After infusion of a high LC flow with TFA (0.45 mL/min) for 50 min, a massive decrease in signal is observed when infusing a calibration mix. This is due to the negative influence of TFA on ionisation.

Visual representation of the comparison between FA and TFA for toxin charge states.

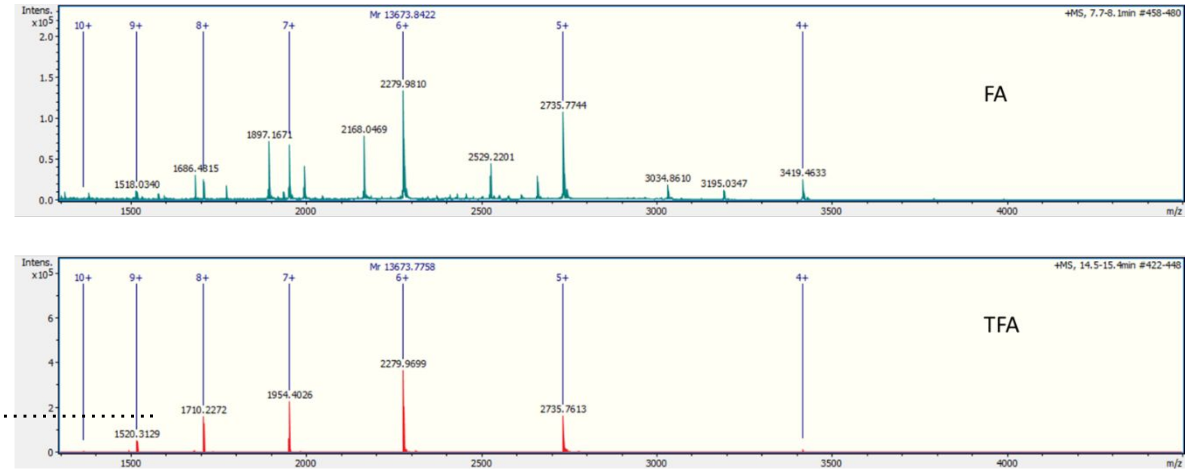

In this figure the comparison is made between the charge states of a venom toxin when using FA and TFA. The charge states between FA and TFA are highly similar and results in roughly the same accurate mass of the toxin. In addition, a “cleaner” spectrum is observed due to better separation of venom toxins while using TFA.

Suppl. Table 3. Comparison of the proteomics results between the 60 and 14.4 min nanoLC gradient.

**Method**	**Species**	**Database**	**Protein accession**	**Toxin class**	**Protein score**	**Sequence coverage**
60 min nanoLC method	*Calloselasma rhodostoma*	Uniprot	VSPF1_CALRH	SVSP	1450	46,2
			VSPF2_CALRH	SVSP	564	18,6
			SLEA_CALRH	CTL	510	71,4
14.4 min nanoLC method	*Calloselasma rhodostoma*	Uniprot	VSPF1_CALRH	SVSP	692	40,6
			VSPF2_CALRH	SVSP	154	8,9
			SLEA_CALRH	CTL	497	67,7

The nanoLC gradient was shortened from 60 to 14.4 min in order to increase the throughput of the methodology. Shortening the gradient will have a negative effect on the separation of peptides, which leads to a decrease in found protein scores and sequence coverages as observed in table 2 above. However, a lesser separation did not lead to a decrease in the number of identified venom toxins.

7g: Superimposed PSCs, UV and MS data for *Crotalus atrox, Echis ocellatus, Naja nigricollis* and *Naja pallida* analysed under optimised HT Venomics conditions.

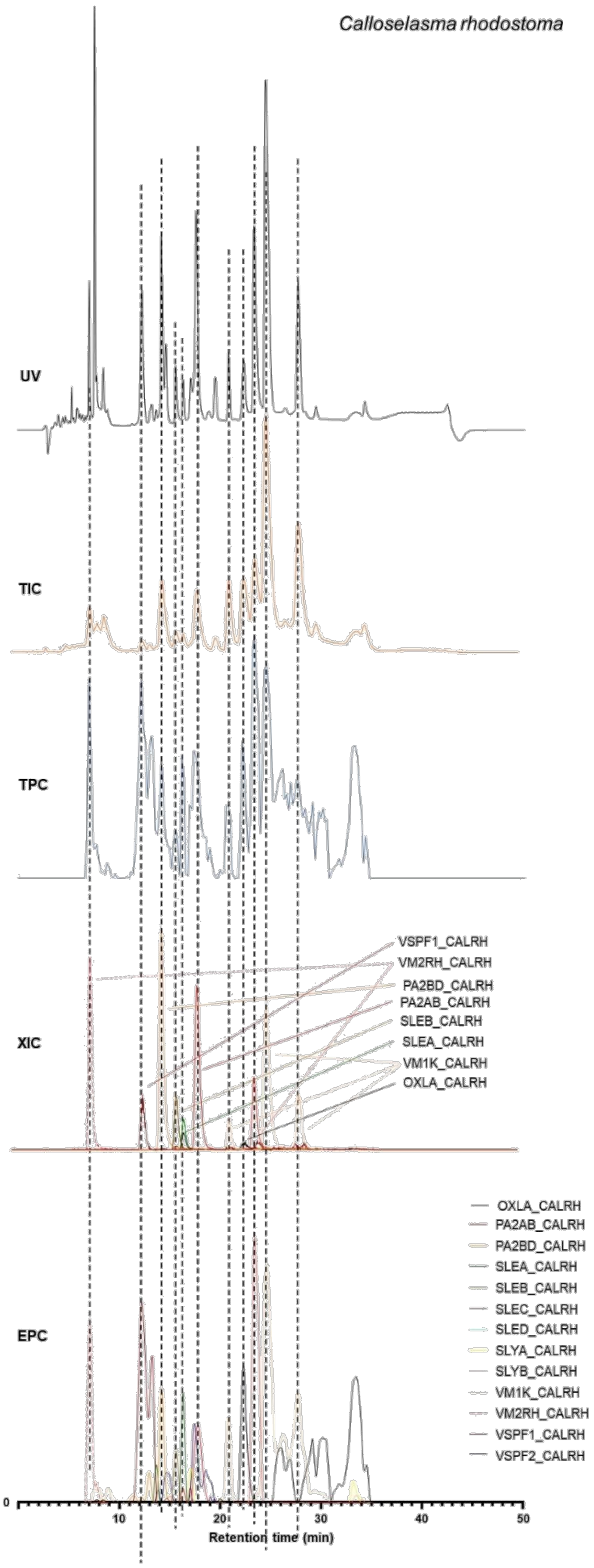

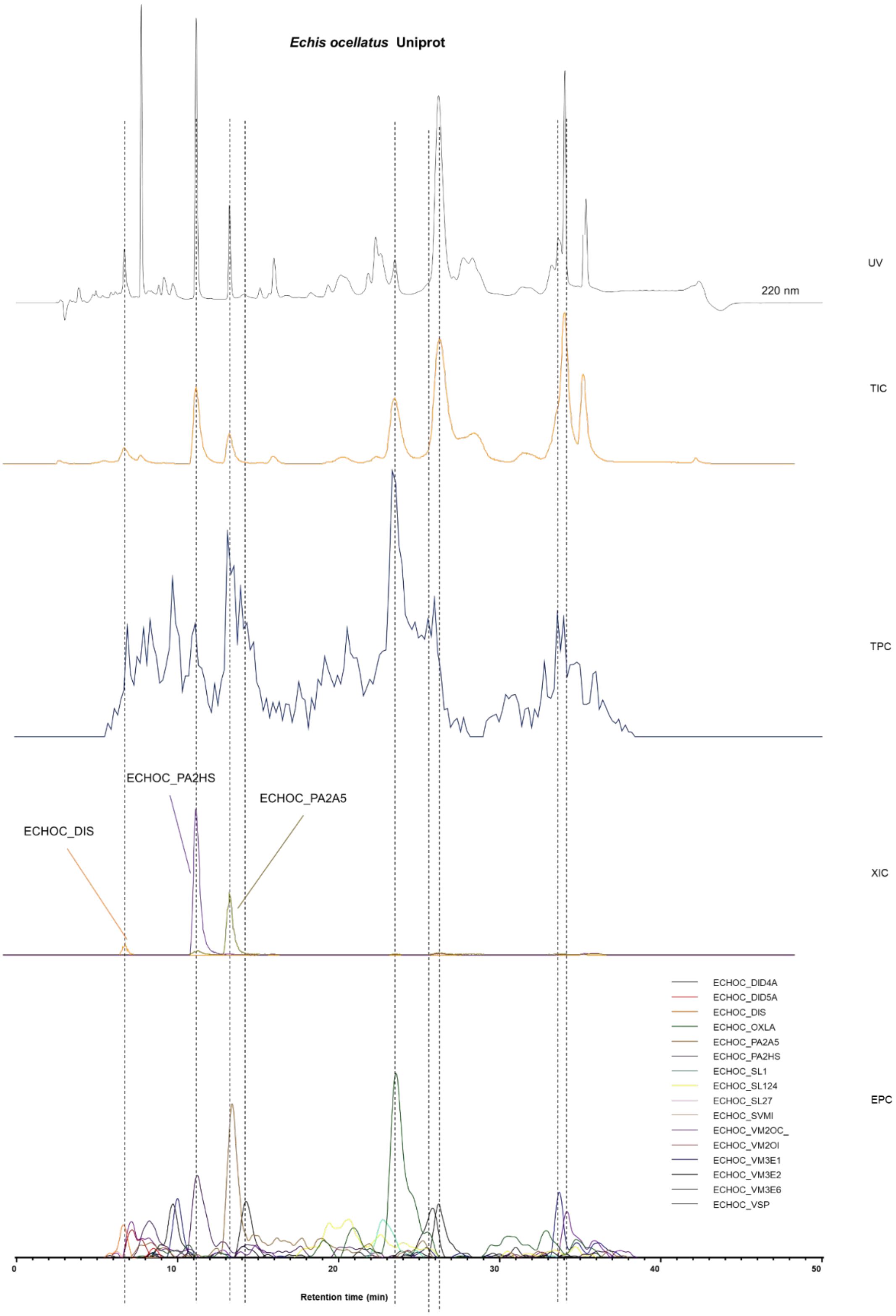

Echis ocellatus Uniprot
UV
220 nm
TIC
TPC
XIC
ECHOC_DIS
ECHOC_PA2HS
ECHOC_PA2A5
EPC
ECHOC_DID4A
ECHOC_DID5A
ECHOC_DIS
ECHOC_OXLA
ECHOC_PA2A5
ECHOC_PA2HS
ECHOC_SL1
ECHOC_SL124
ECHOC_SL27
ECHOC_SVMI
ECHOC_VM2OC_
ECHOC_VM2OI
ECHOC_VM3E1
ECHOC_VM3E2
ECHOC_VM3E6
ECHOC_VSP
0
10
20
30
40
50
Retention time (min)

LC-UV-MS-PSC data superimposed from *Echis ocellatus*. The data for *Echis ocellatus* from the three detection techniques used in this study were superimposed in order to obtain a comprehensive figure that facilitates identification of venom toxins. The top graph shows the UV (220 nm) trace. The 2nd graph from the top shows the Total Ion Chromatogram (TIC) from the mass spectrometry data. The middle trace shows the Total Protein Chromatogram (TPC), consisting of the sum of all protein scores obtained from the proteomics data. The 2nd to last graph from the bottom shows the Extracted Ion Chromatograms (XICs) obtained from the mass spectrometry data. The XICs shown here are believed to match to the toxin IDs found in the bottom graph (PSCs) based on their matching retention times and peak shapes. The bottom graph shows the PSCs, which represent the individual venom proteins found with Mascot database searching of the digested contents in the wells.

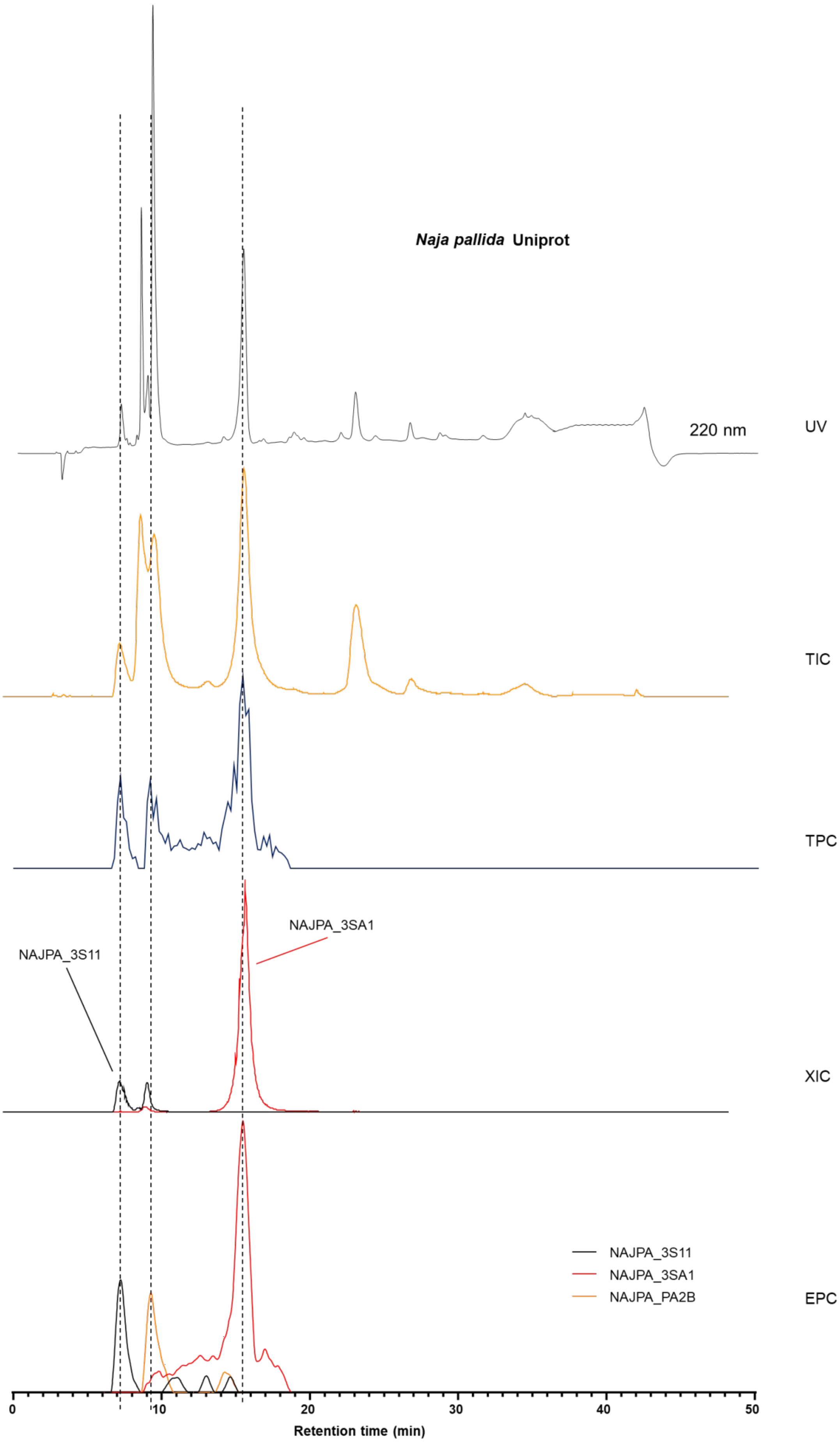
Naja pallida Uniprot
220 nm
UV
TIC
TPC
NAJPA_3SA1
NAJPA_3S11
XIC
NAJPA_3S11
NAJPA_3SA1
NAJPA_PA2B
EPC
0
10
20
30
40
50
Retention time (min)

LC-UV-MS-PSC data superimposed from *Naja pallida*. The data for *Naja pallida* from the three detection techniques used in this study were superimposed in order to obtain a comprehensive figure that facilitates identification of venom toxins. The top graph shows the UV (220 nm) trace. The 2nd graph from the top shows the Total Ion Chromatogram (TIC) from the mass spectrometry data. The middle trace shows the Total Protein Chromatogram (TPC), consisting of the sum of all protein scores obtained from the proteomics data. The 2nd to last graph from the bottom shows the Extracted Ion Chromatograms (XICs) obtained from the mass spectrometry data. The XICs shown here are believed to match to the toxin IDs found in the bottom graph (PSCs) based on their matching retention times and peak shapes. The bottom graph shows the PSCs, which represent the individual venom proteins found with Mascot database searching of the digested contents in the wells.

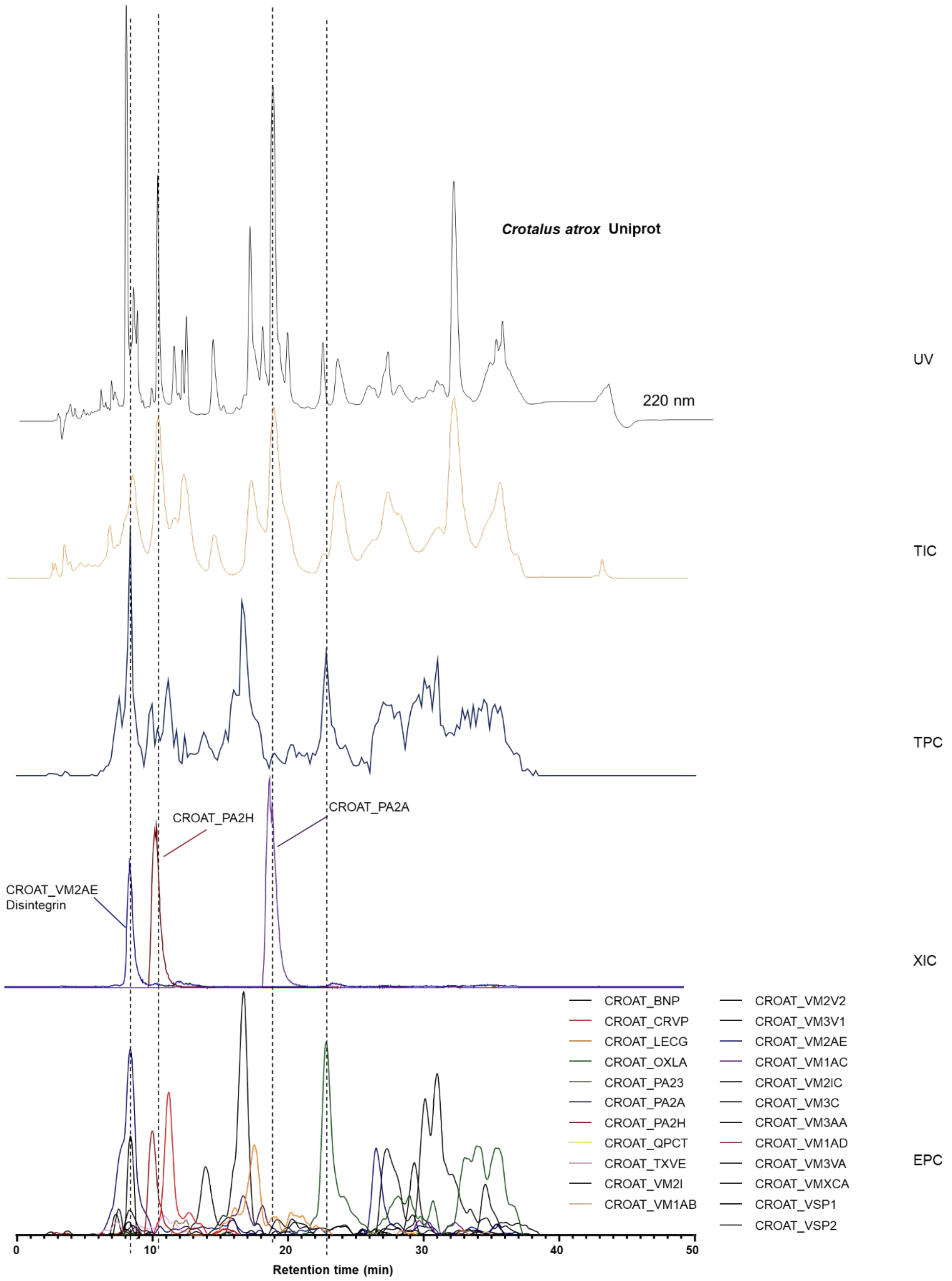

Crotalus atrox Uniprot
UV
220 nm
TIC
TPC
CROAT_PA2H
CROAT_PA2A
CROAT_VM2AE
Disintegrin
XIC
CROAT_BNP
CROAT_CRVP
CROAT_LECG
CROAT_OXLA
CROAT_PA23
CROAT_PA2A
CROAT_PA2H
CROAT_QPCT
CROAT_TXVE
CROAT_VM2I
CROAT_VM1AB
CROAT_VM2V2
CROAT_VM3V1
CROAT_VM2AE
CROAT_VM1AC
CROAT_VM2IC
CROAT_VM3C
CROAT_VM3AA
CROAT_VM1AD
CROAT_VM3VA
CROAT_VMXCA
CROAT_VSP1
CROAT_VSP2
EPC
0
10
20
30
40
50
Retention time (min)

LC-UV-MS-PSC data superimposed from *Crotalus atrox*. The data for *Crotalus atrox* from the three detection techniques used in this study were superimposed in order to obtain a comprehensive figure that facilitates identification of venom toxins. The top graph shows the UV (220 nm) trace. The 2nd graph from the top shows the Total Ion Chromatogram (TIC) from the mass spectrometry data. The middle trace shows the Total Protein Chromatogram (TPC), consisting of the sum of all protein scores obtained from the proteomics data. The 2nd to last graph from the bottom shows the Extracted Ion Chromatograms (XICs) obtained from the mass spectrometry data. The XICs shown here are believed to match to the toxin IDs found in the bottom graph (PSCs) based on their matching retention times and peak shapes. The bottom graph shows the PSCs, which represent the individual venom proteins found with Mascot database searching of the digested contents in the wells.

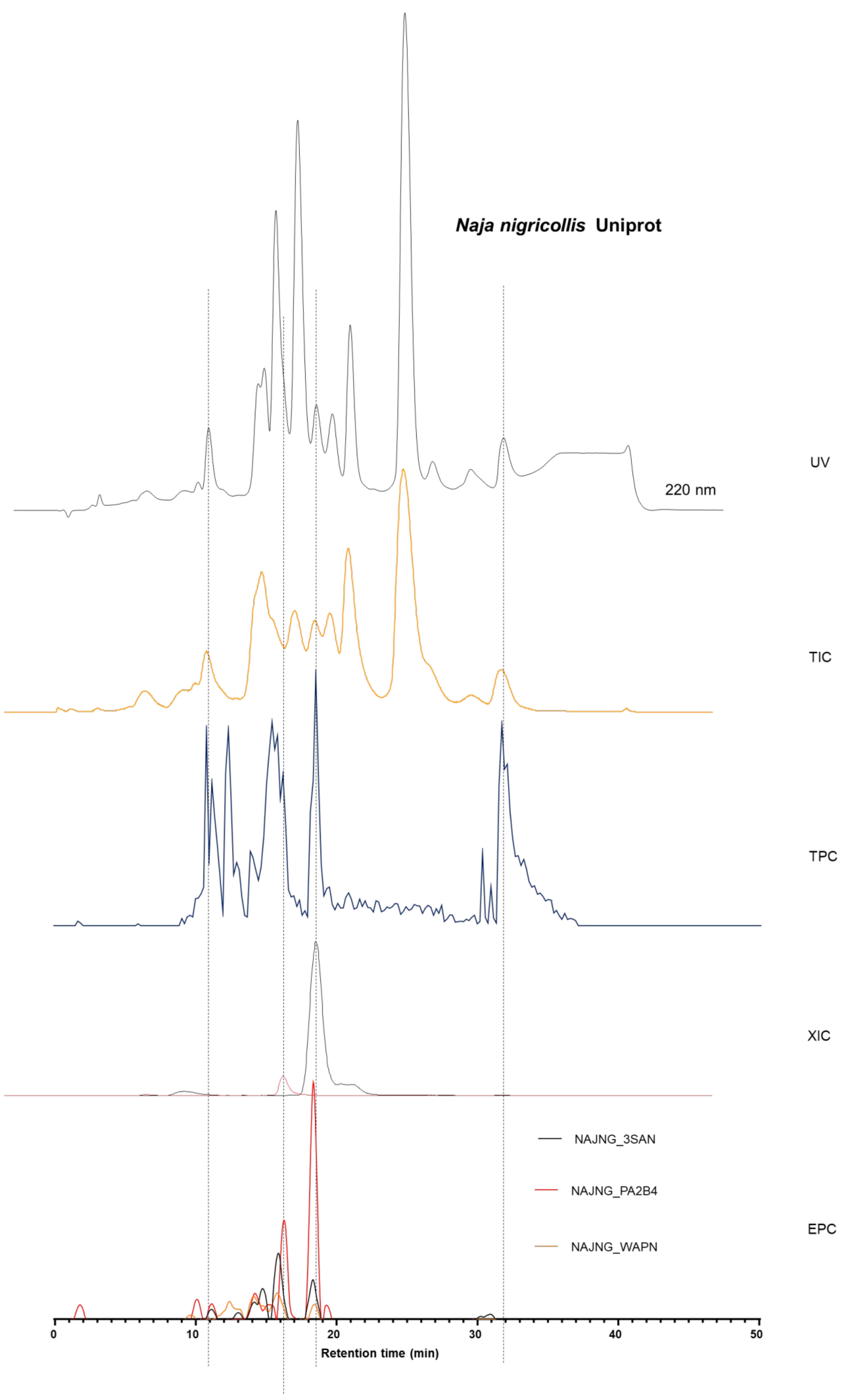

Naja nigricollis Uniprot
UV
220 nm
TIC
TPC
XIC
NAJNG_3SAN
NAJNG_PA2B4
NAJNG_WAPN
EPC
0
10
20
30
40
50
Retention time (min)

LC-UV-MS-PSC data superimposed from *Naja nigricollis*. The data for *Naja nigricollis* from the three detection techniques used in this study were superimposed in order to obtain a comprehensive figure that facilitates identification of venom toxins. The top graph shows the UV (220 nm) trace. The 2nd graph from the top shows the Total Ion Chromatogram (TIC) from the mass spectrometry data. The middle trace shows the Total Protein Chromatogram (TPC), consisting of the sum of all protein scores obtained from the proteomics data. The 2nd to last graph from the bottom shows the Extracted Ion Chromatograms (XICs) obtained from the mass spectrometry data. The XICs shown here are believed to match to the toxin IDs found in the bottom graph (PSCs) based on their matching retention times and peak shapes. The bottom graph shows the PSCs, which represent the individual venom proteins found with Mascot database searching of the digested contents in the wells.

7h: Superimposed PSCs and UV data for *Bungarus multicinctus*, *Daboia russelii*, *Naja naja, Ophoiphagus hannah, Naja mossambica and Bothrops asper* analysed under optimised HT Venomics conditions.

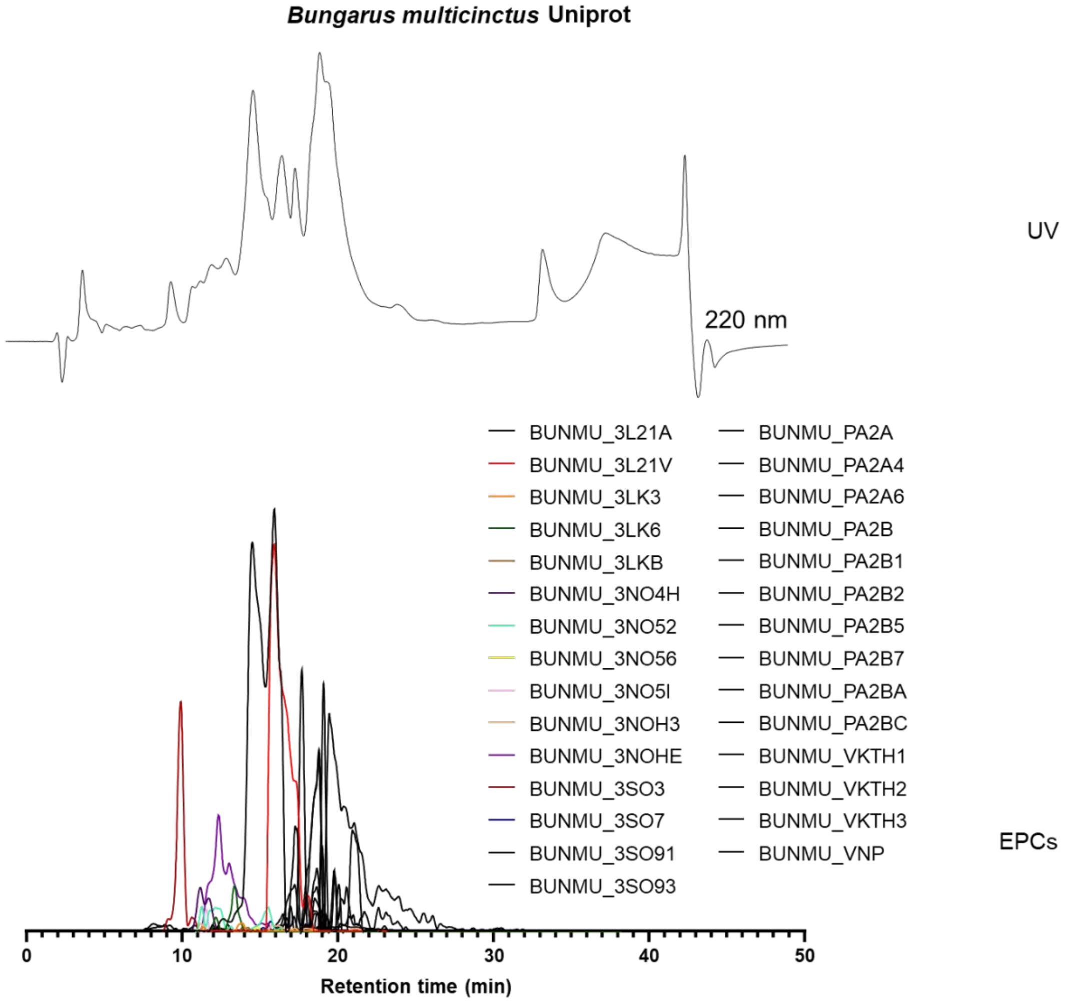

\LC-UV-PSC data superimposed from *Bungarus multicintus*. The data for *Bungarus multicintus* from the two detection techniques used in this study were superimposed in order to obtain a comprehensive figure that facilitates identification of venom toxins. The top graph shows the UV (220 nm) trace. The bottom graph shows the PSCs, which represent the individual venom proteins found with Mascot database searching of the digested contents in the wells.

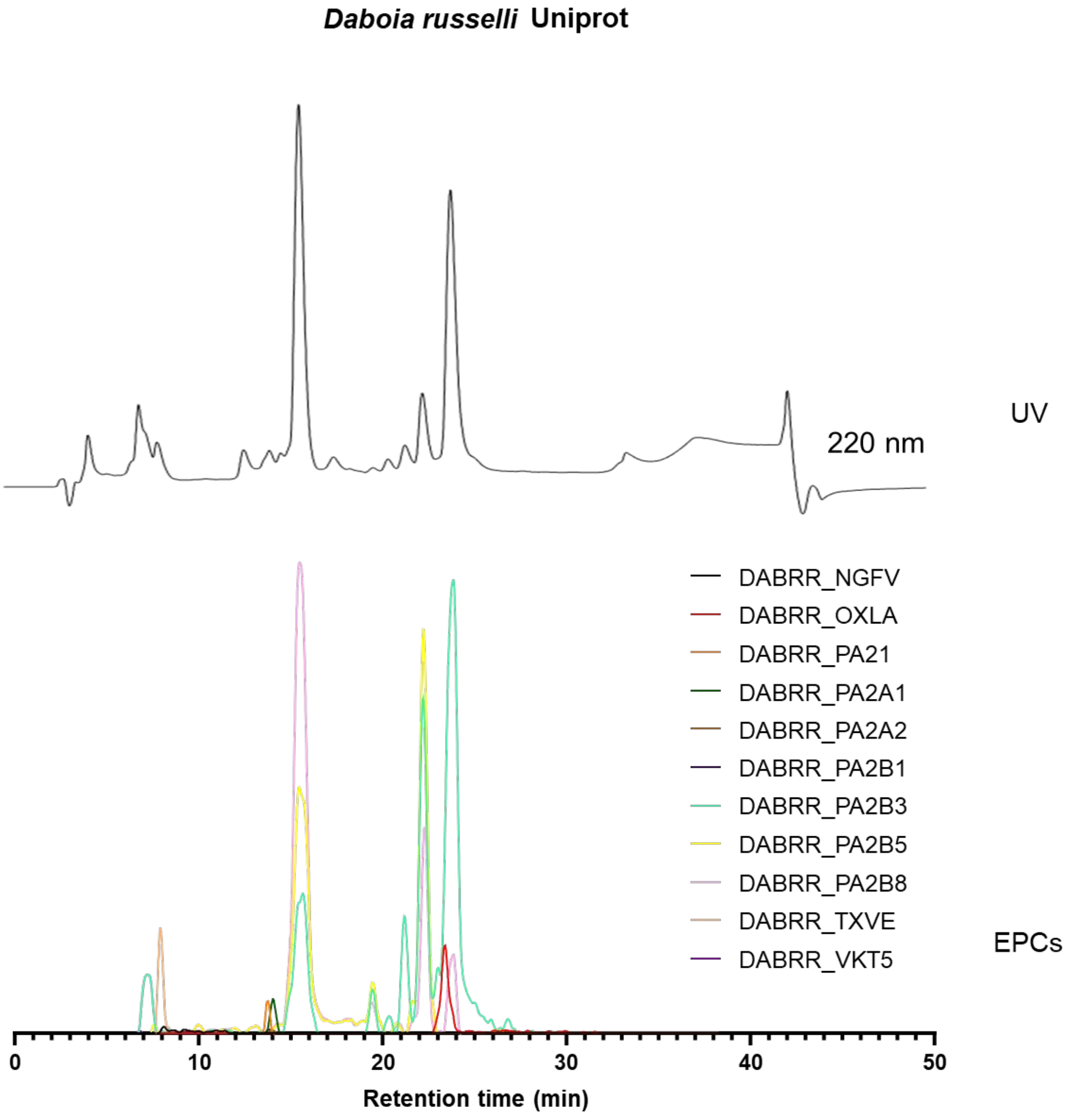

LC-UV-PSC data superimposed from *Daboia russelii*. The data for *Daboia russelii* from the two detection techniques used in this study were superimposed in order to obtain a comprehensive figure that facilitates identification of venom toxins. The top graph shows the UV (220 nm) trace. The bottom graph shows the PSCs, which represent the individual venom proteins found with Mascot database searching of the digested contents in the wells.

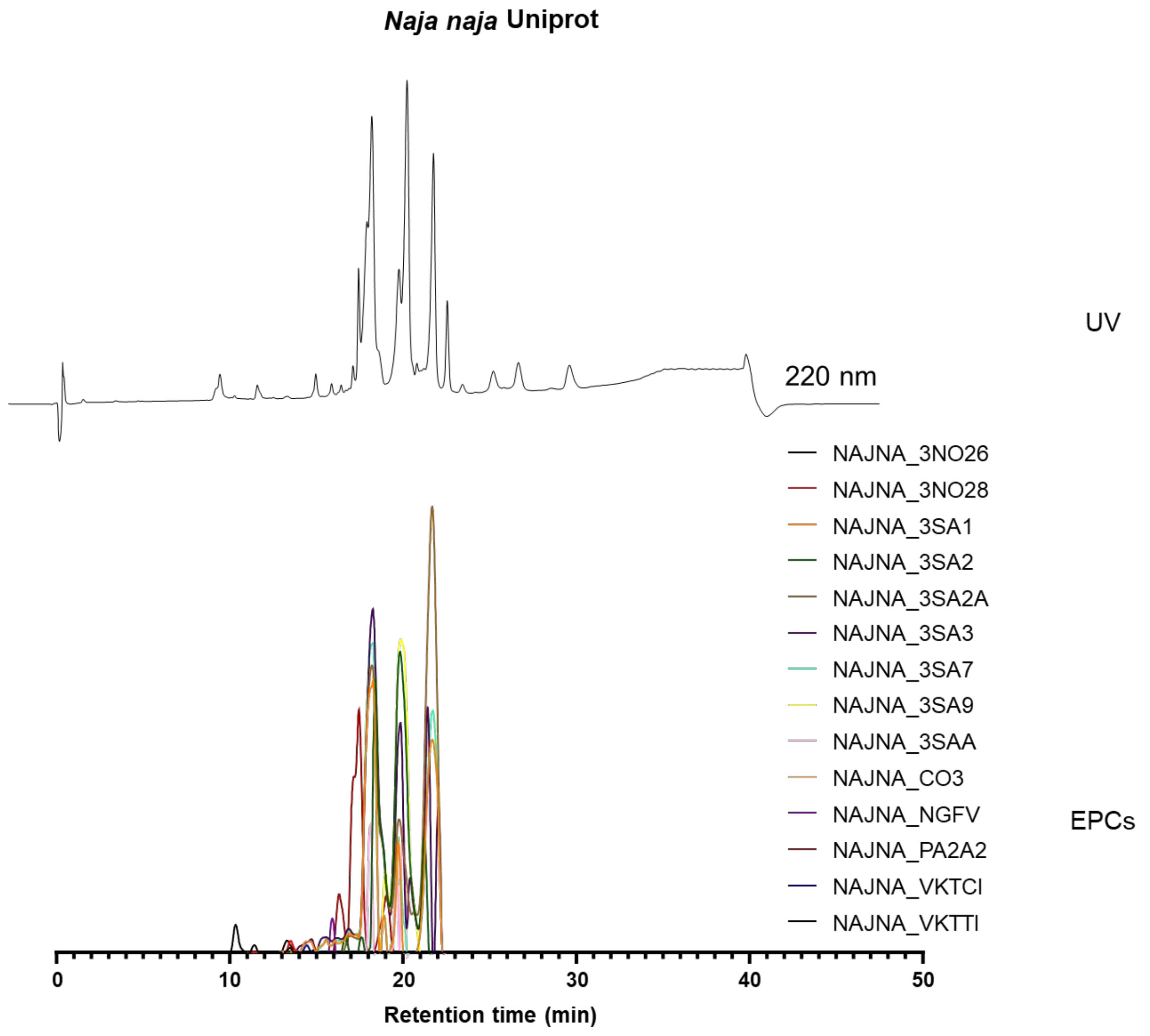

LC-UV-PSC data superimposed from *Naja naja*. The data for *Naja naja* from the two detection techniques used in this study were superimposed in order to obtain a comprehensive figure that facilitates identification of venom toxins. The top graph shows the UV (220 nm) trace. The bottom graph shows the PSCs, which represent the individual venom proteins found with Mascot database searching of the digested contents in the wells.

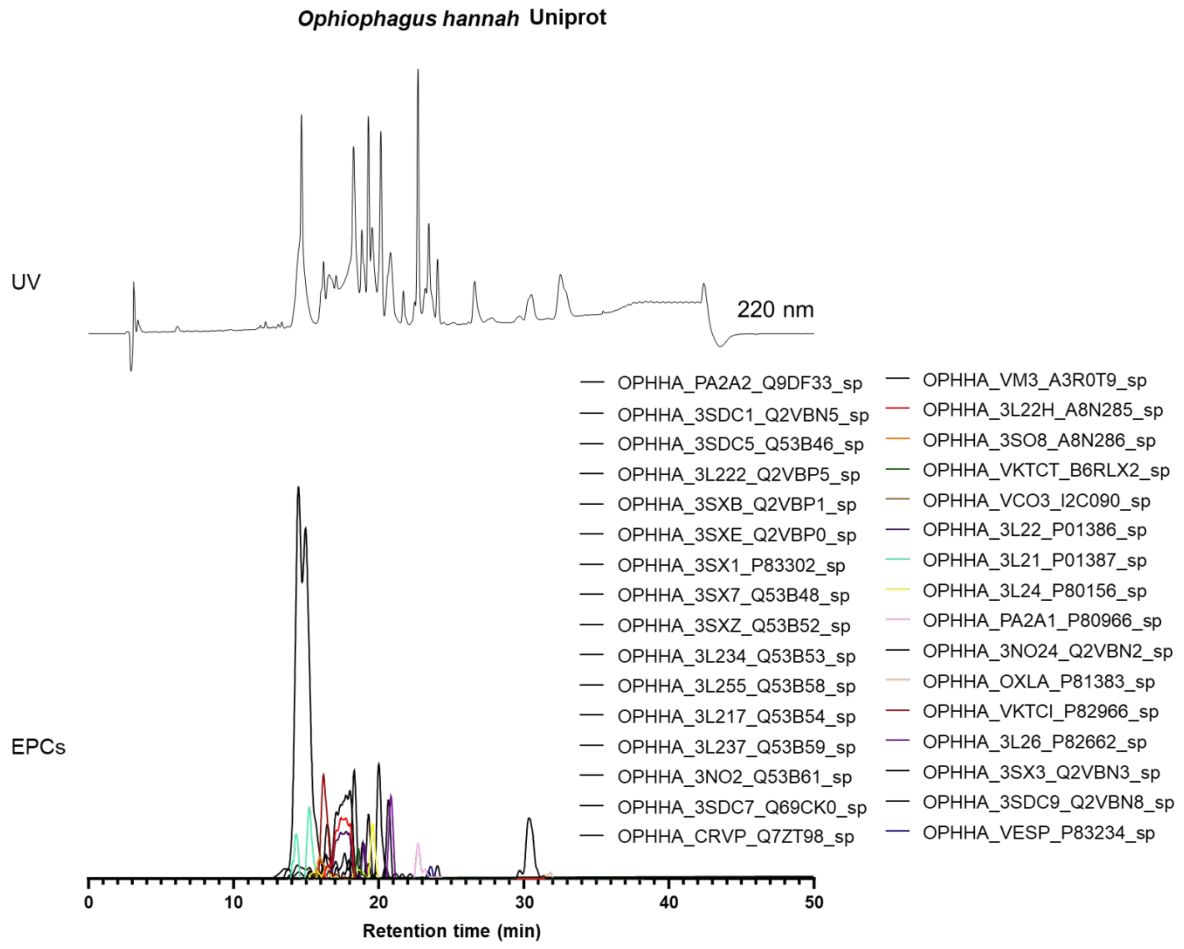

LC-UV-PSC data superimposed from *Ophiophagus hannah*. The data for *Ophiophagus hannah* from the two detection techniques used in this study were superimposed in order to obtain a comprehensive figure that facilitates identification of venom toxins. The top graph shows the UV (220 nm) trace. The bottom graph shows the PSCs, which represent the individual venom proteins found with Mascot database searching of the digested contents in the wells.

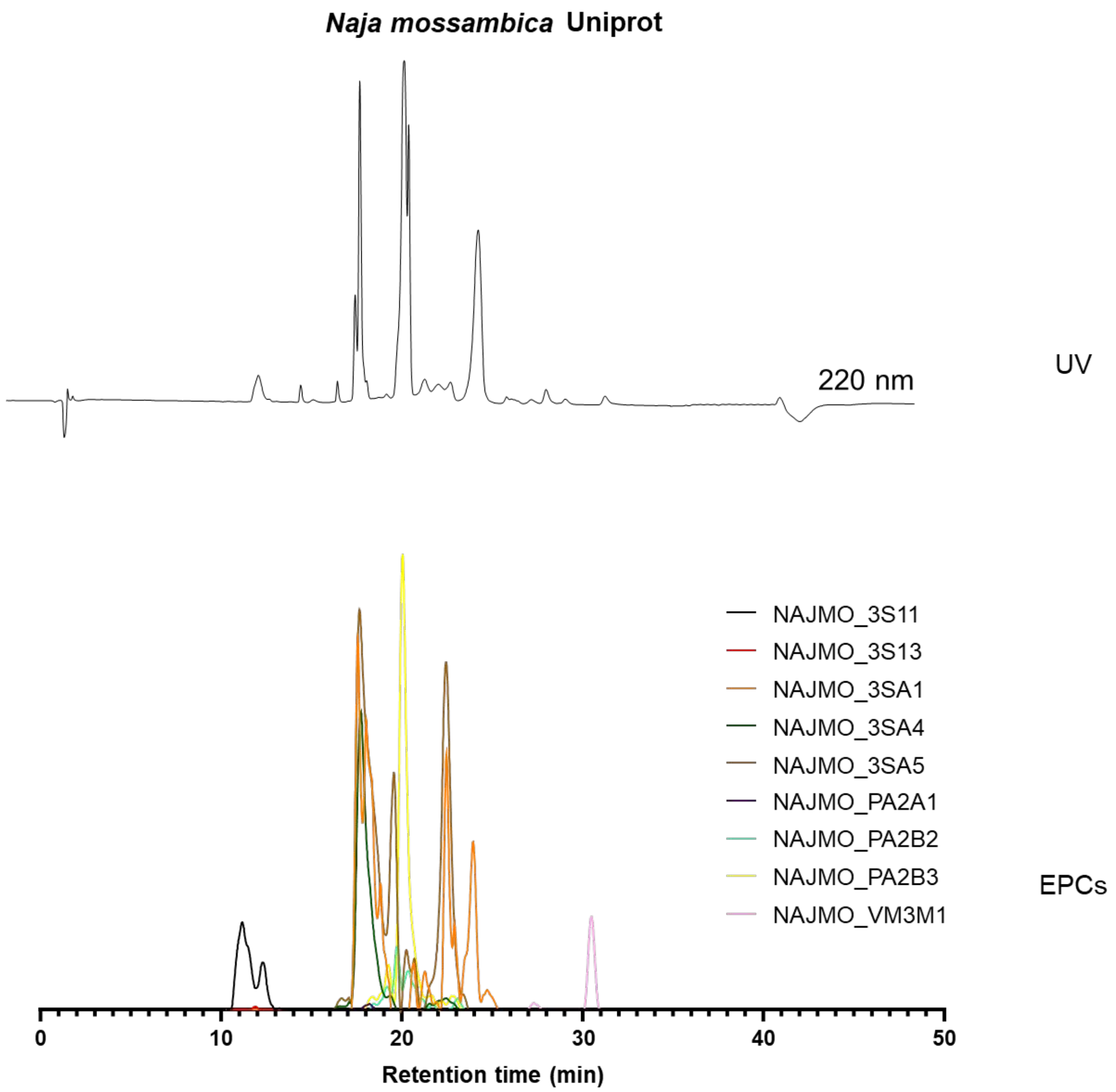

LC-UV-PSC data superimposed from *Naja mossambica*. The data for *Naja mossambica* from the two detection techniques used in this study were superimposed in order to obtain a comprehensive figure that facilitates identification of venom toxins. The top graph shows the UV (220 nm) trace. The bottom graph shows the PSCs, which represent the individual venom proteins found with Mascot database searching of the digested contents in the wells.

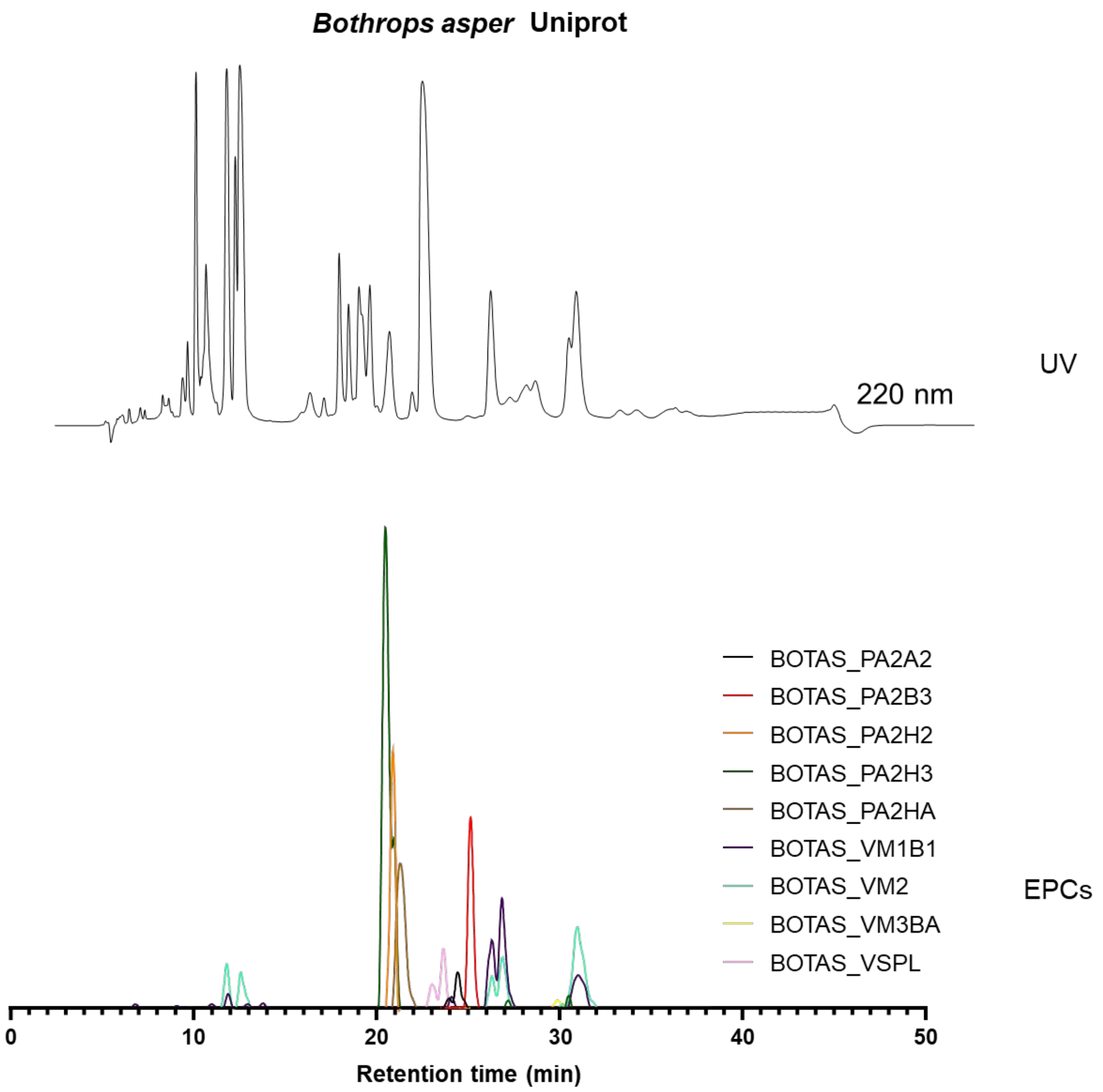

LC-UV-PSC data superimposed from *Bothrops asper*. The data for *Bothrops asper* from the two detection techniques used in this study were superimposed in order to obtain a comprehensive figure that facilitates identification of venom toxins. The top graph shows the UV (220 nm) trace. The bottom graph shows the PSCs, which represent the individual venom proteins found with Mascot database searching of the digested contents in the wells.
