# Supplementary material for: High-Throughput Venomics
Source: J Proteome Res. 2023 Apr 3;22(6):1734–46. doi: 10.1021/acs.jproteome.2c00780 (PMC10243144; doi:10.1021/acs.jproteome.2c00780)
Supplement: Supplementary file 3 — pr2c00780_si_003.zip [file pr2c00780_si_003.zip › All UV Data for paper.pptx]

## Slide 1
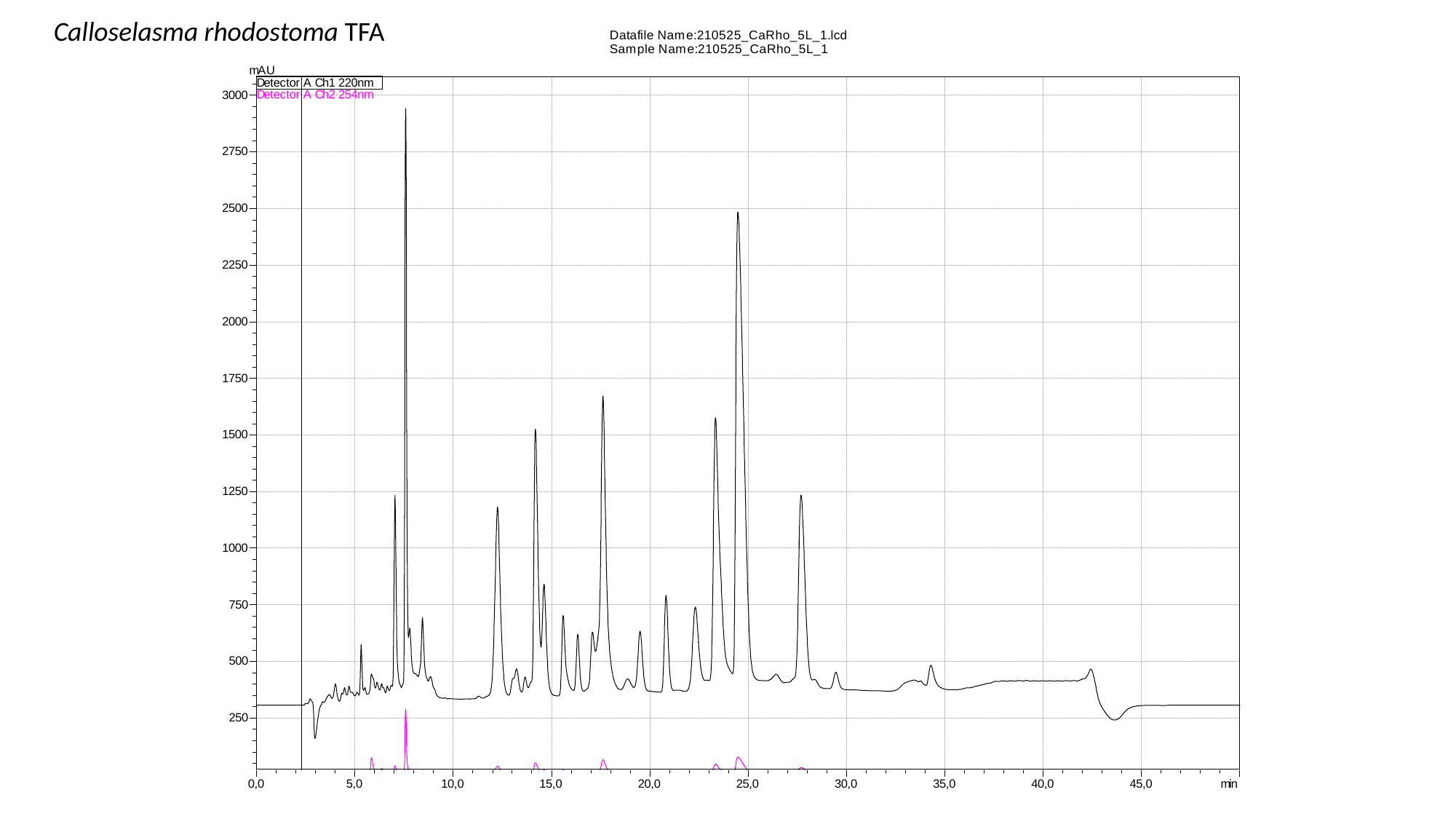

Calloselasma rhodostoma TFA

## Slide 2
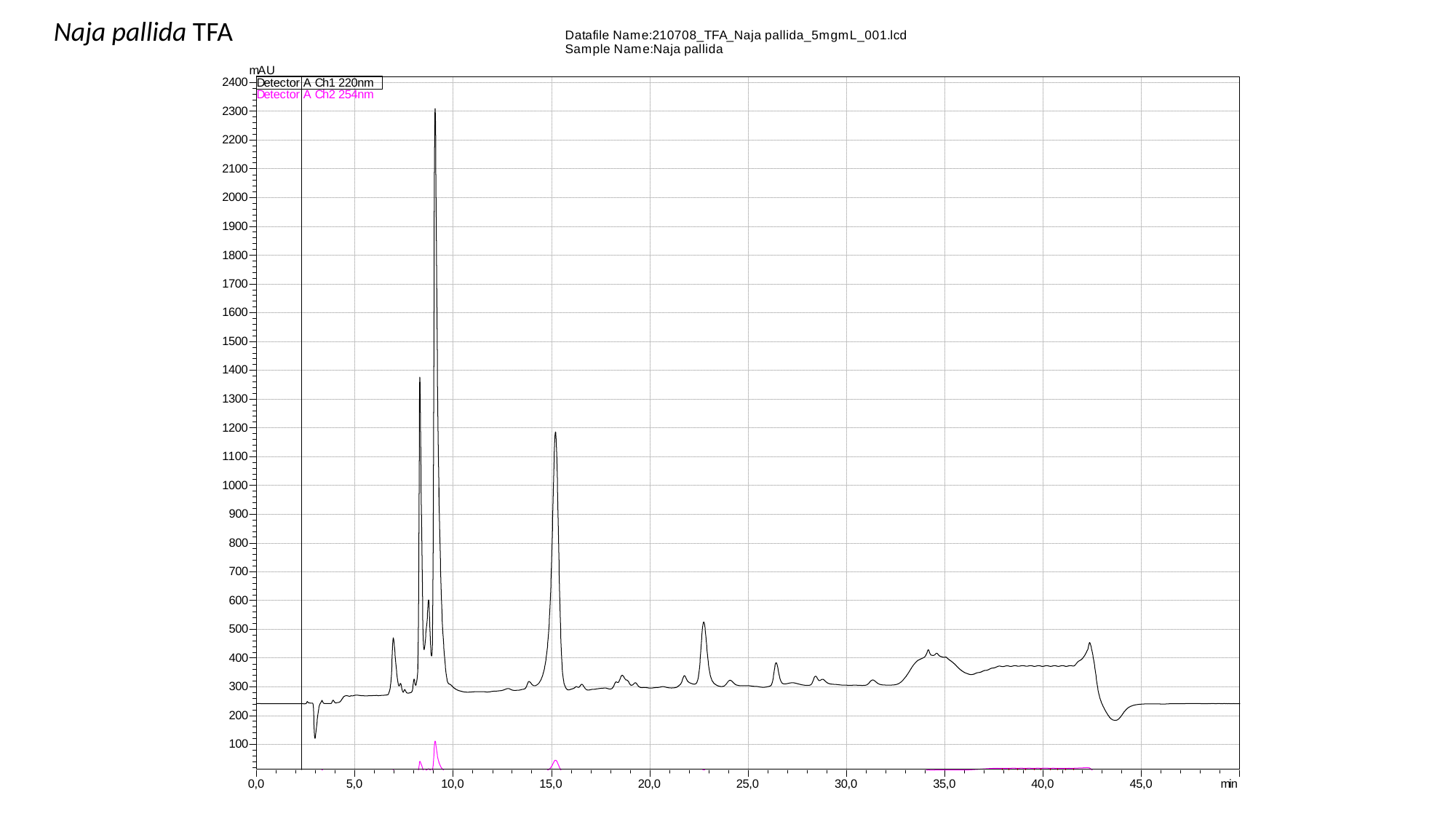

Naja pallida TFA

## Slide 3
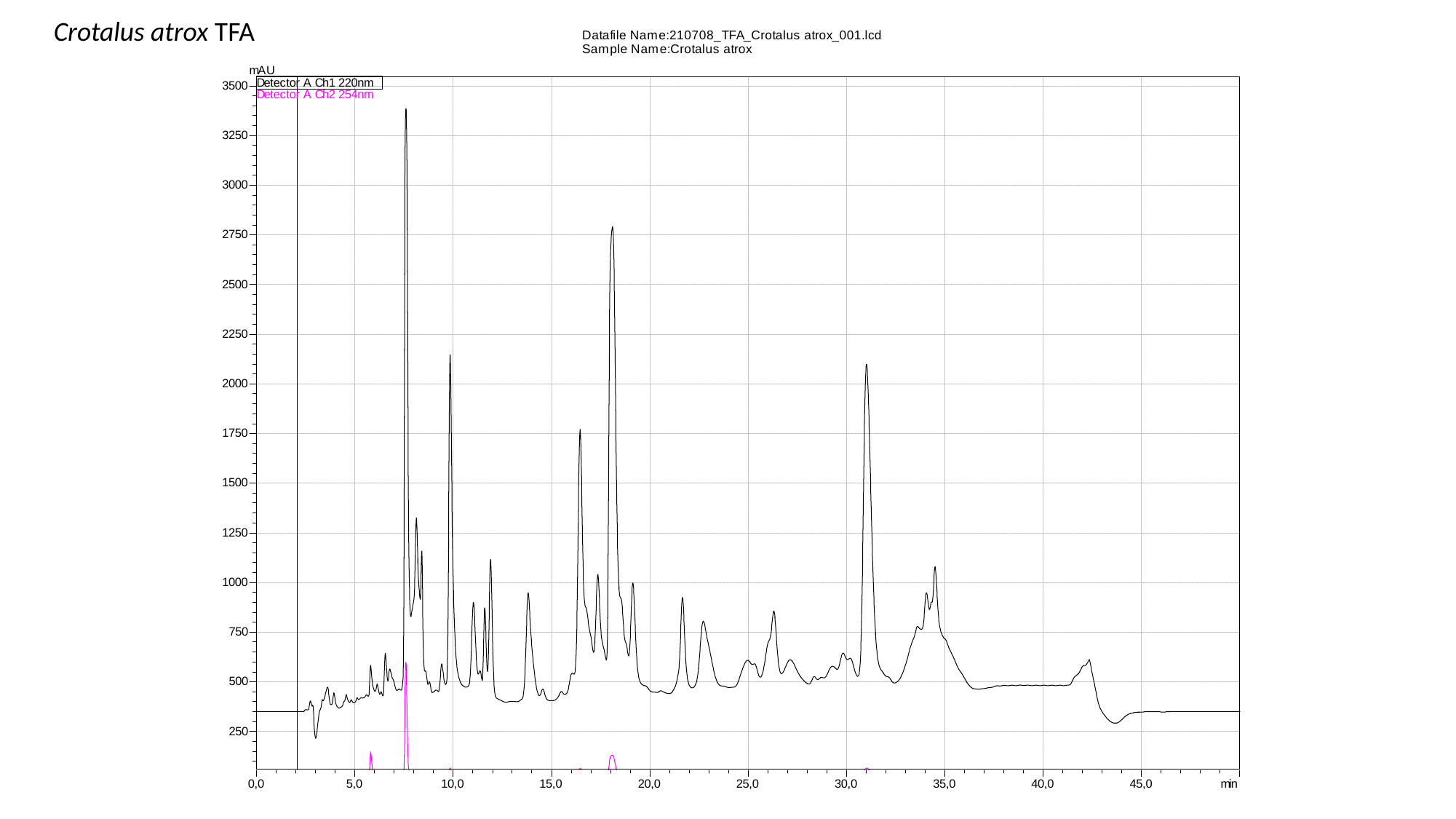

Crotalus atrox TFA

## Slide 4
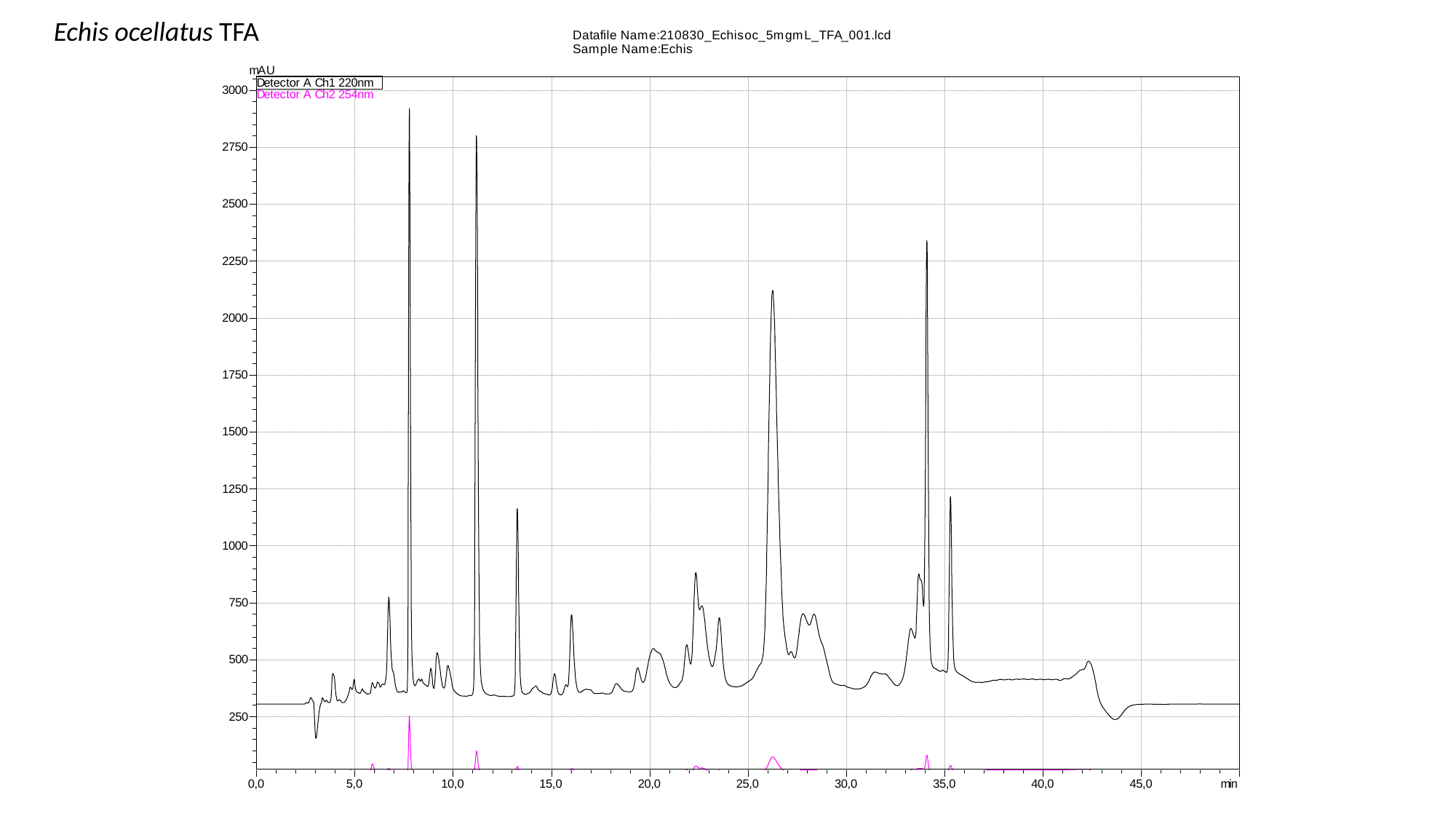

Echis ocellatus TFA

## Slide 5
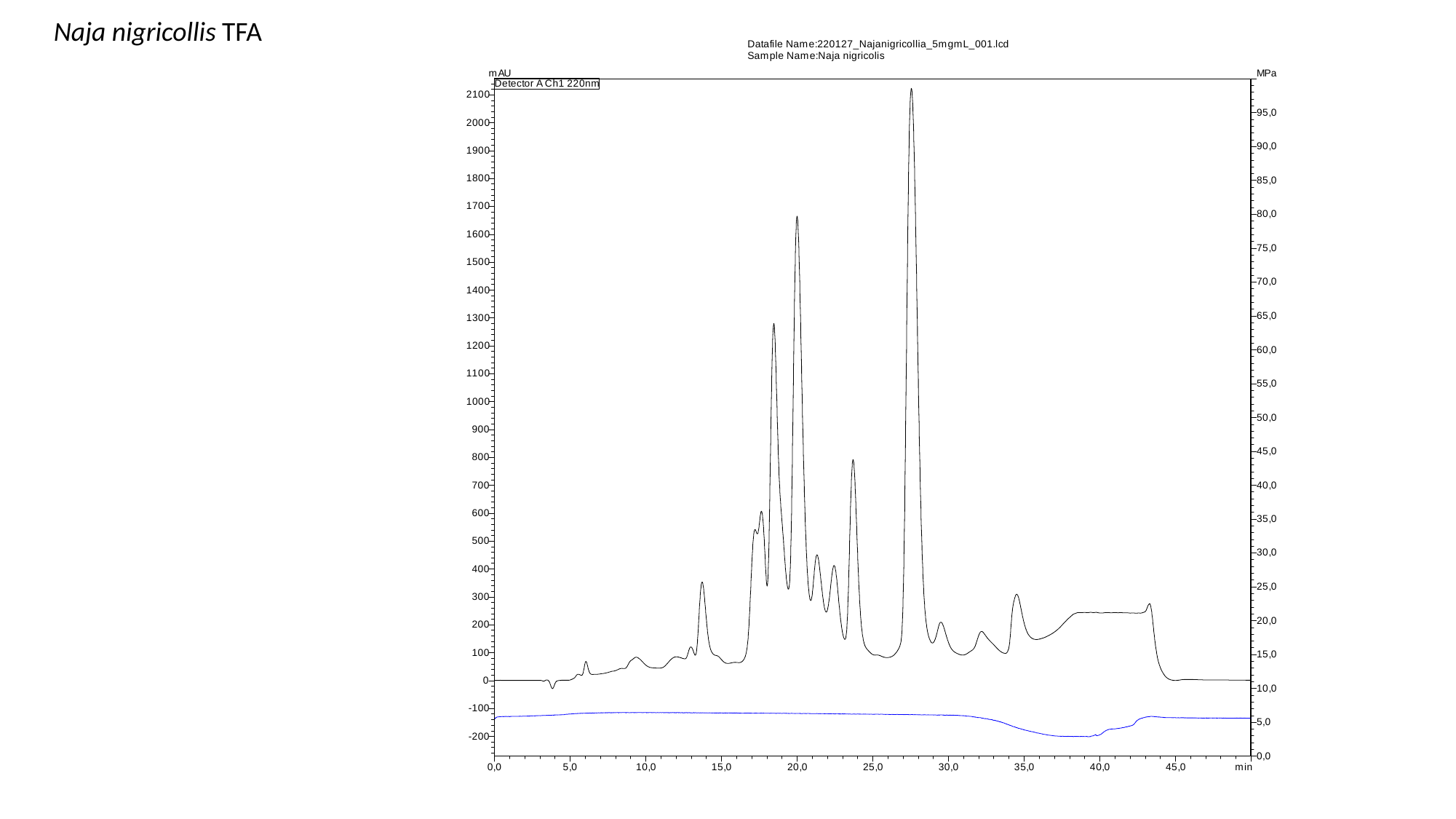

Naja nigricollis TFA

## Slide 6
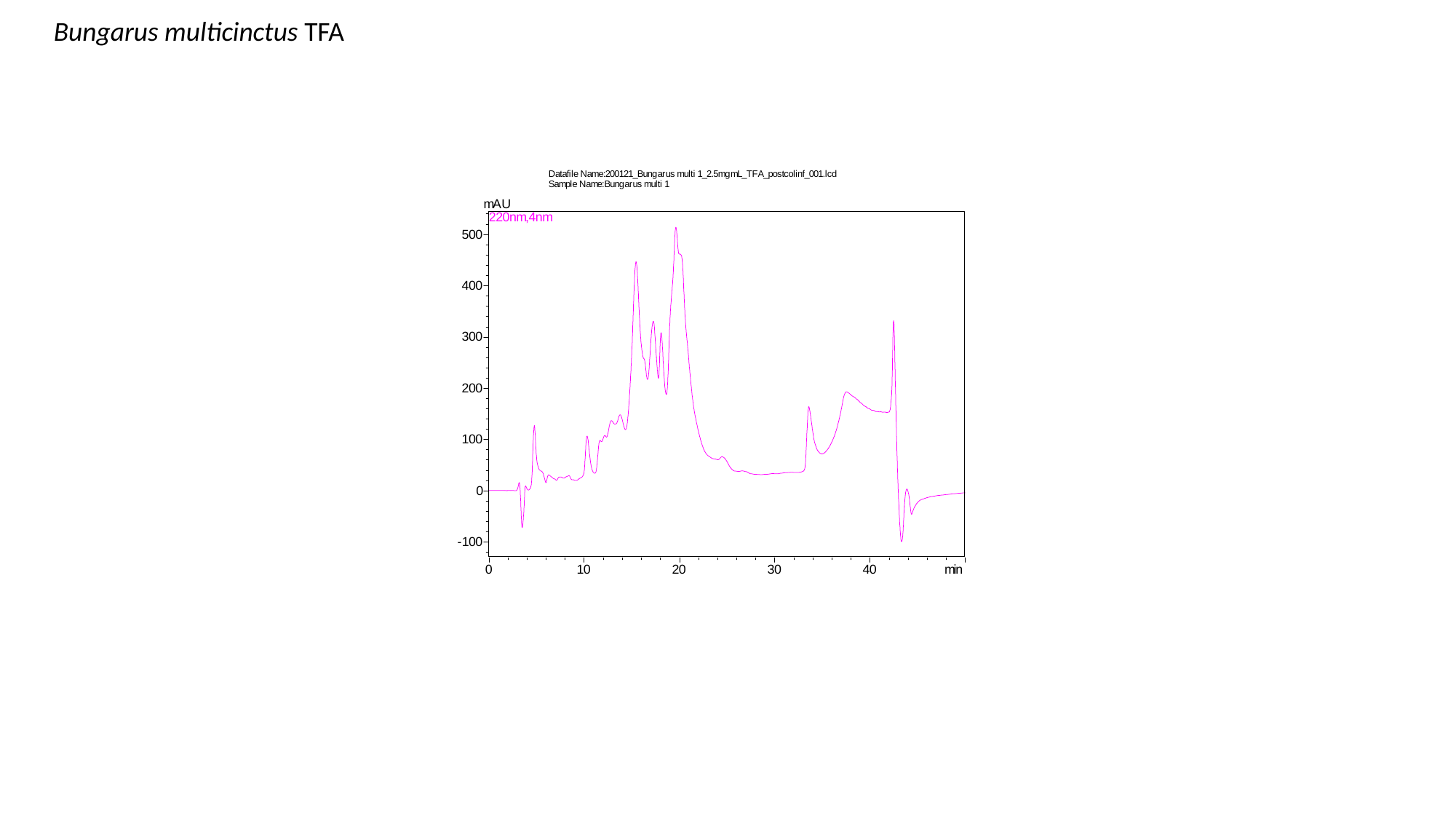

Bungarus multicinctus TFA

## Slide 7
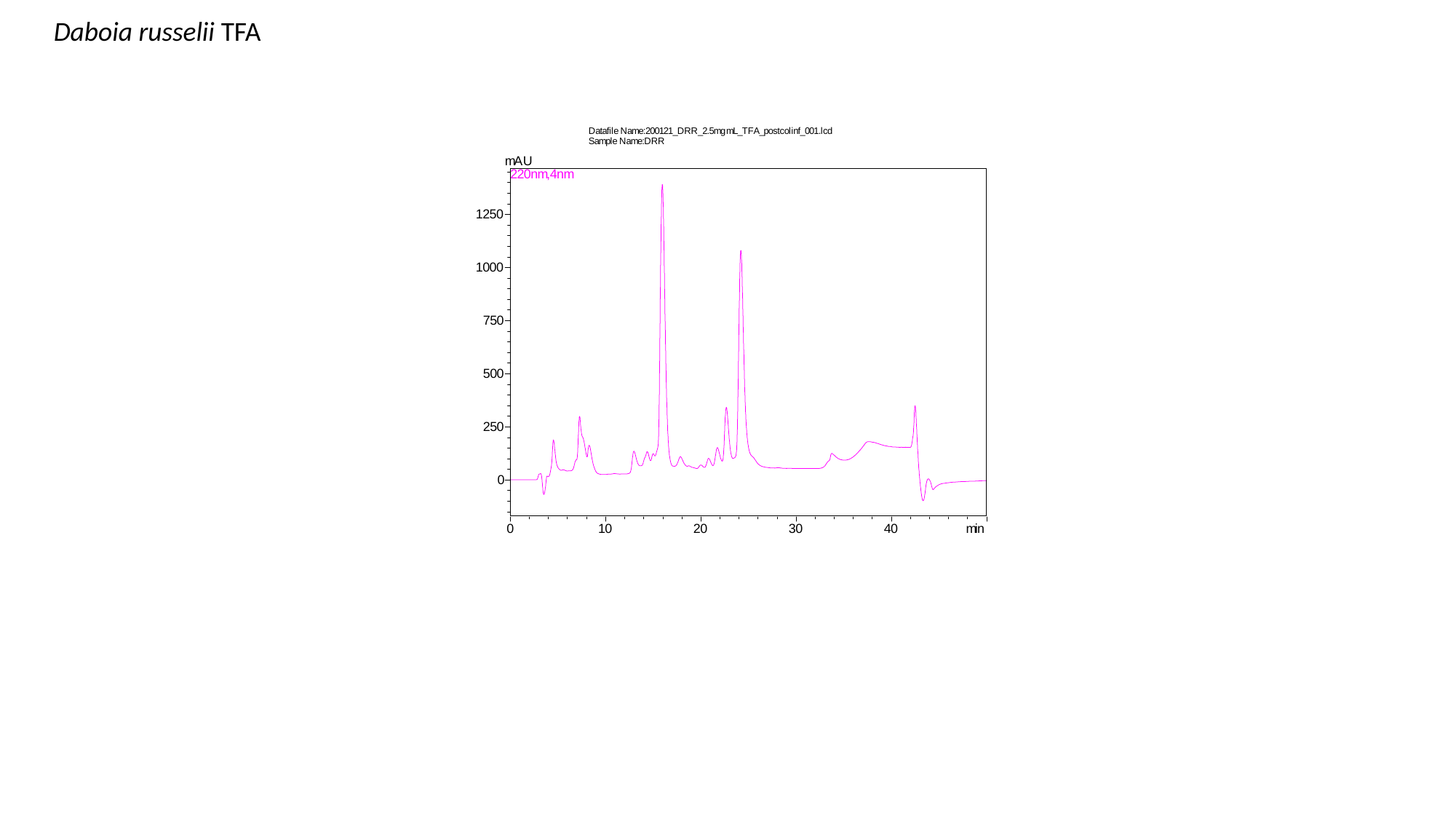

Daboia russelii TFA

## Slide 8
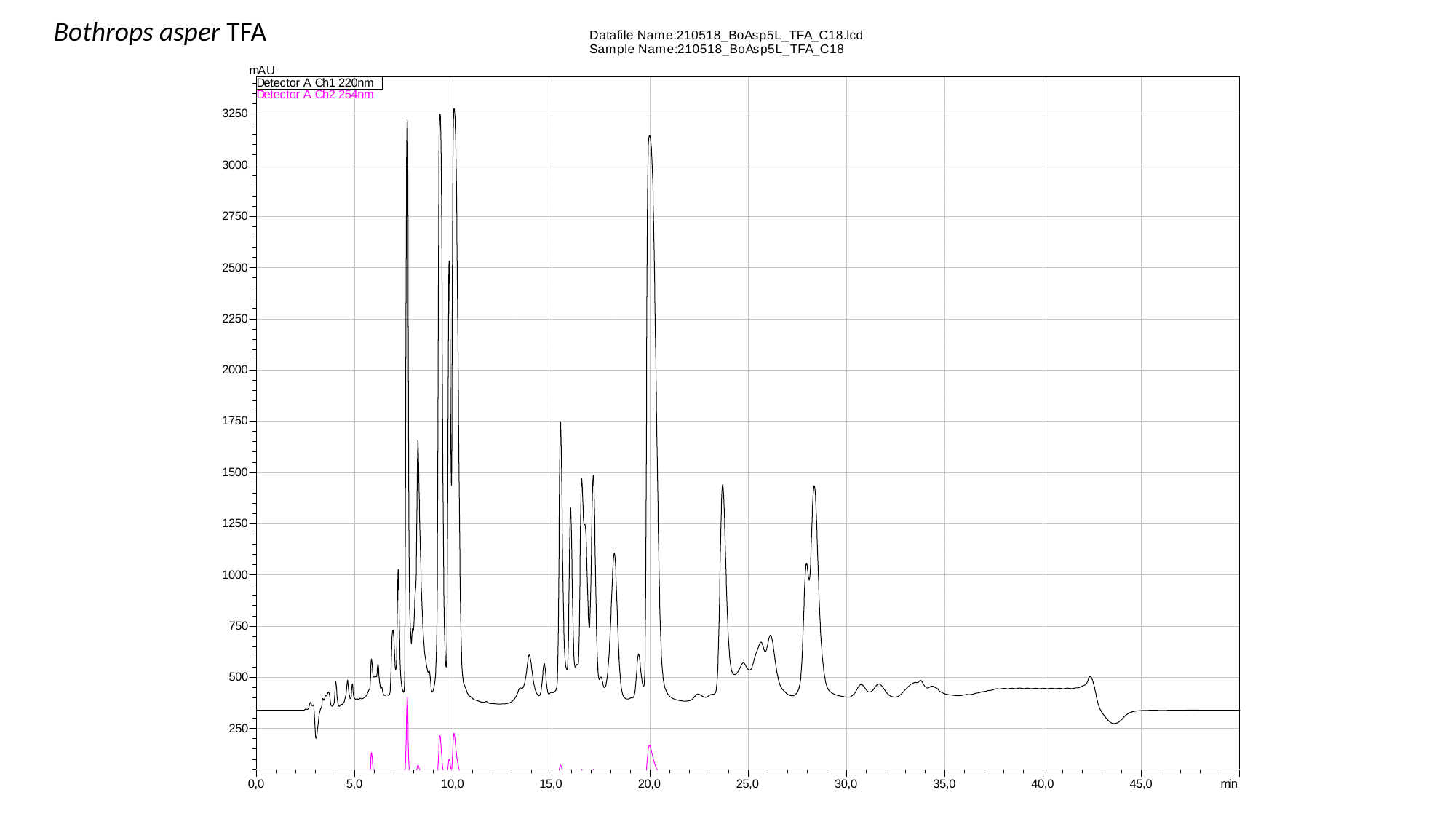

Bothrops asper TFA

## Slide 9
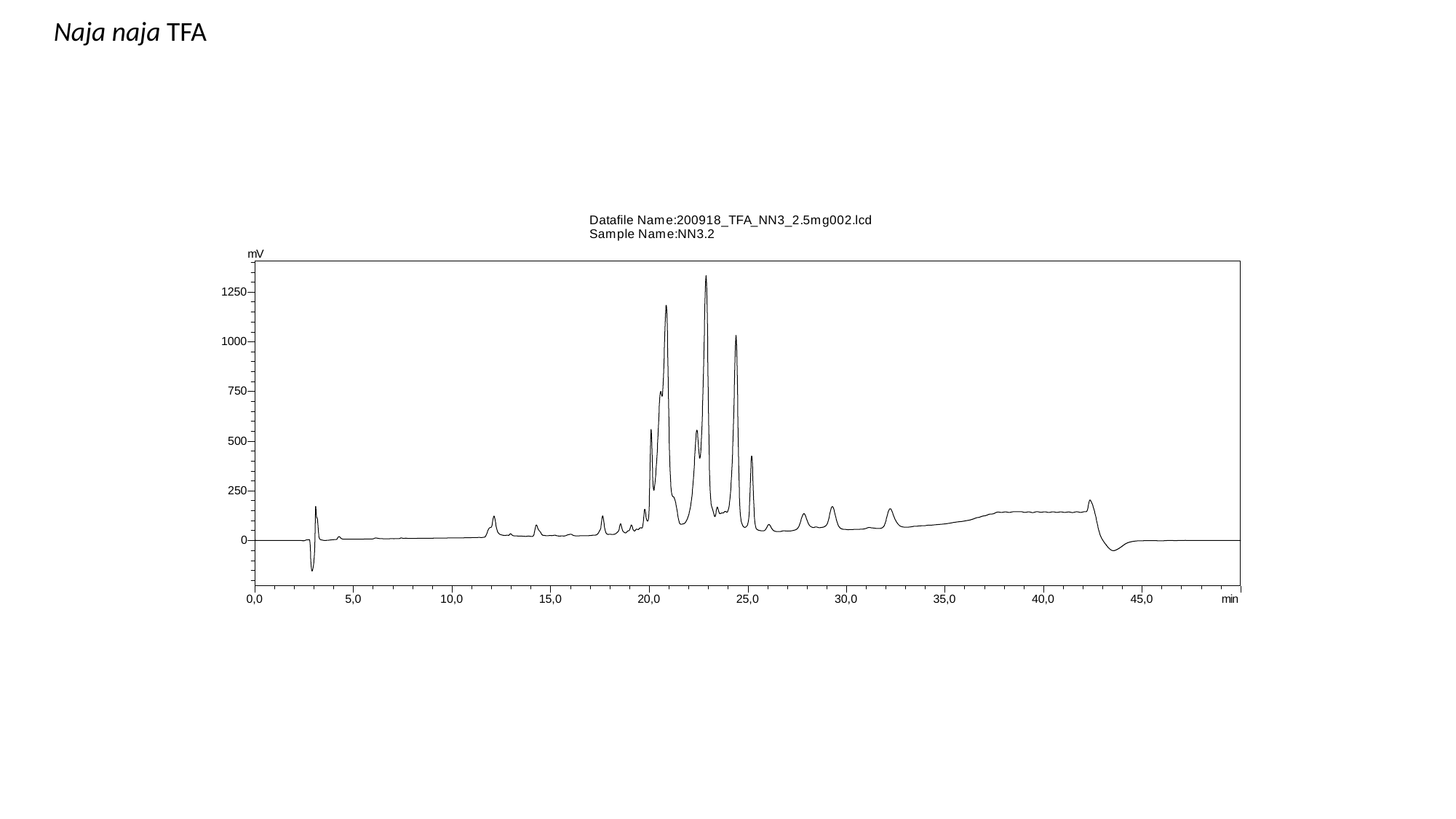

Naja naja TFA

## Slide 10
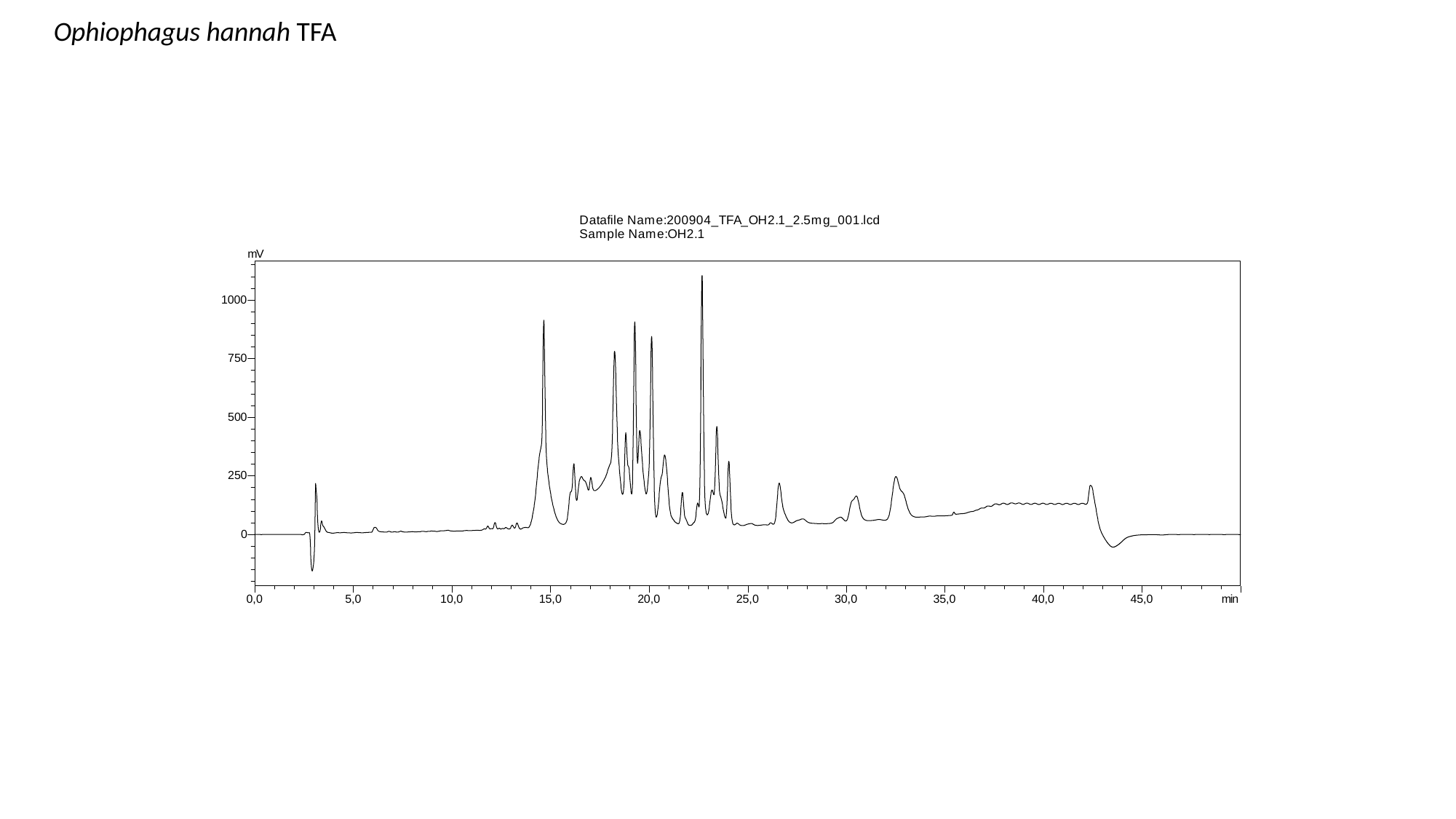

Ophiophagus hannah TFA

## Slide 11
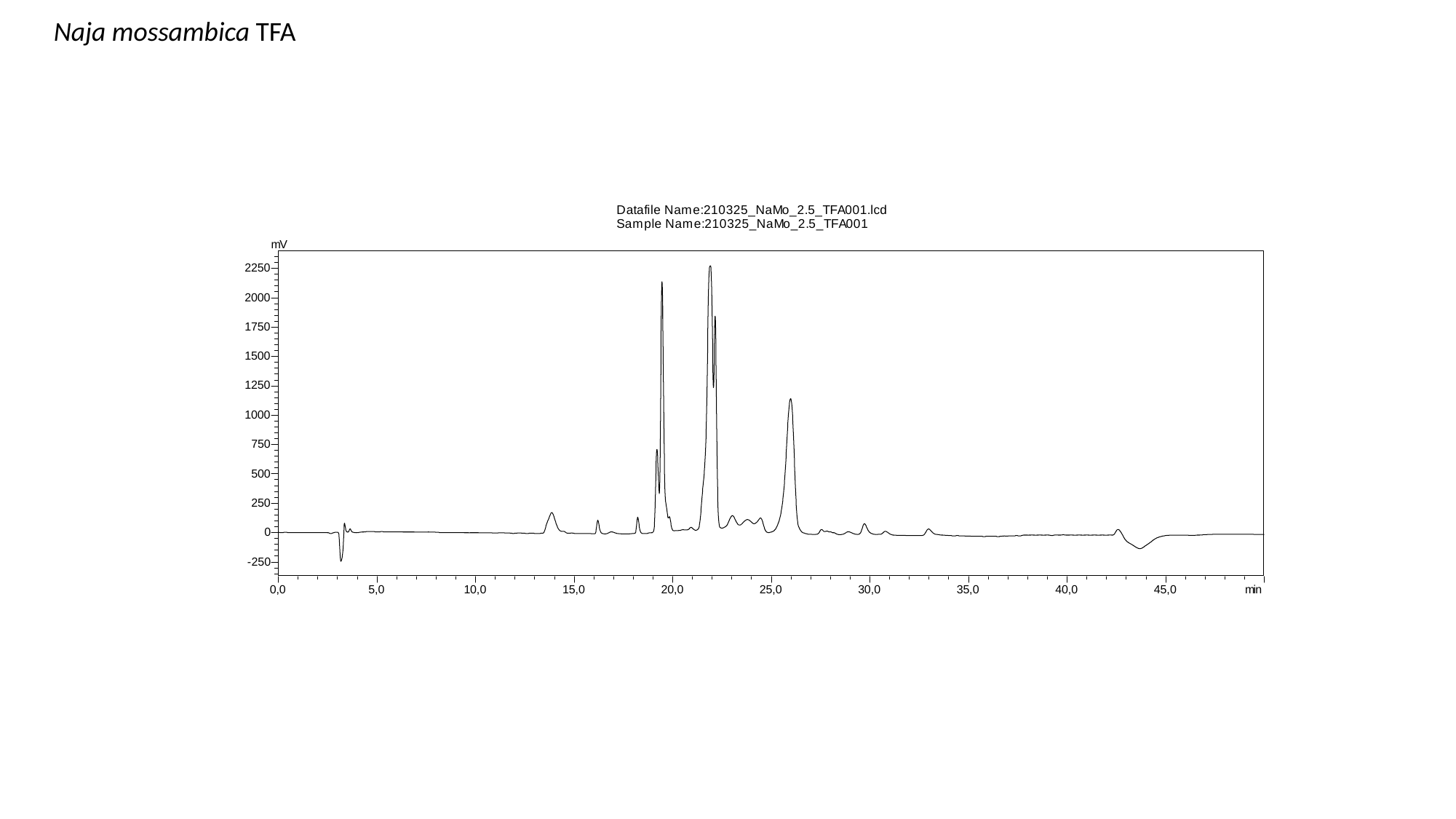

Naja mossambica TFA
