# Supplementary material for: High-Throughput Venomics
Source: J Proteome Res. 2023 Apr 3;22(6):1734–46. doi: 10.1021/acs.jproteome.2c00780 (PMC10243144; doi:10.1021/acs.jproteome.2c00780)
Supplement: Supplementary file 9 — pr2c00780_si_009.zip [file pr2c00780_si_009.zip › Manual files/Manual HT Venomics workflow.docx]

**HT Venomics workflow for Mascot database searches**

The workflow described in this manual was performed on snake venoms that were separated with RPLC and fractionated onto 384-well plates. In-solution tryptic digestions were performed onto the toxins fractionated in the wells of the well plates and the contents of each of the wells were then analysed with nanoLC-MS/MS. The MS and MS/MS data obtained were then converted into MGF files (using the software of the mass spectrometer used; in our case Bruker software).

*Note: Each of these (MGF files containing) data sets provided in the SI belongs to a single fractionated snake venom. Using these data sets, from each data set protein score chromatograms can be plotted based on the protein scores of the different toxins found in each well for each venom analysed.*

*(Note 2: This plotting of protein score chromatograms (PSCs) is not applicable when well plates containing unique samples in each of the wells are analysed. The complete workflow up to plotting the PSCs is only valid when analysing toxins fractionated from a venom after RPLC on well plates using the complete HT venomics workflow.*

*Note 3: R version 4.2.1 was used.*

**Before the R scripts steps**

1. Convert your MS/MS data to MGF files (since Mascot is used for the database searches in this study). Or use one of the MGF files data sets provided in the SI to work with existing data measured for this study.
2. Submit your MGF files to Mascot for database searching
   1. By using Mascot Daemon you can process all your files in one batch

**R script steps**

1. Open R
2. Download and install the following packages: tidyverse, httr, ggrepel, patchwork and pracma.

R script 1: Extract all CSV files for all your searches from the Mascot search log

1. Look into the MASCOT search log. Look at the first and at the last job number of all searches performed dealing with your data set (Figure 1).
2. Create an Excel file (for example “mascot_export_all_384_wells.xlsx”; this file is provided in the SI) which contains information on your Mascot Database searches including date of search, first job number up to last job number and output file names (Figure 1 and 2)


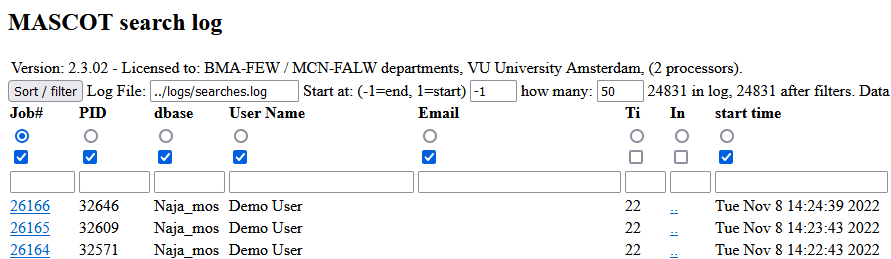


Date

Job number

Figure 1. Example Mascot search log in the Mascot server


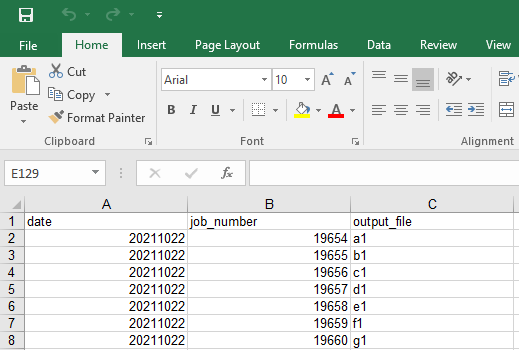


Figure 2. The example Excel file layout needed for R script 1. The date and job numbers are extracted from the Mascot search log and the output file names are filled in by the user themselves. As in this study, fractionations were performed in serpentine fashion over 384-well plates, the output files are names according to well identifier numbers for clarity. An example Excel file with these output file names as template is provided in the SI as Excel document “mascot_export_all_384_wells.xlsx”

1. When you have the Excel file ready with the correct Date and Job numbers and your desired output file names you can load Script 1_export in R by pressing File -> open script.


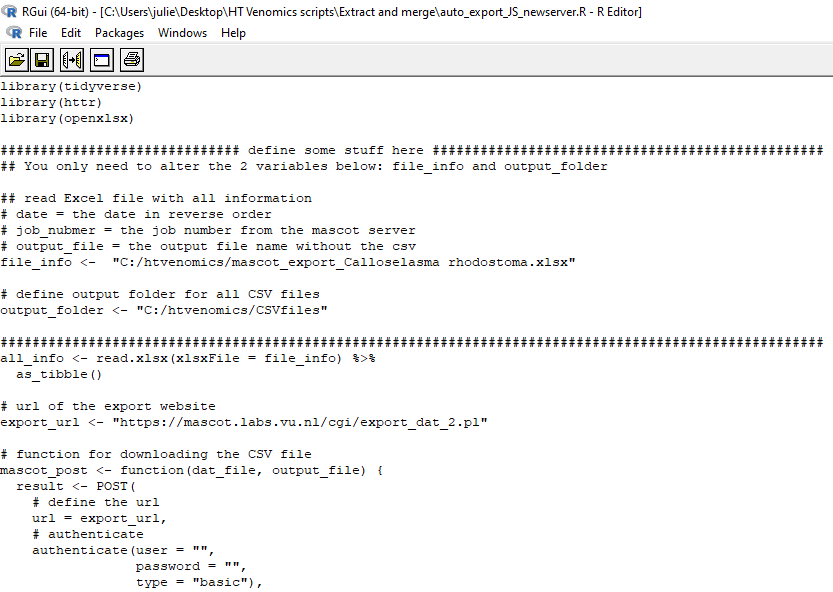


Figure 3. Script 1_export

1. In Figure 3 you see part of Script 1 in which a few things are important to do:
   1. Fill in the location and the name of your Excel file at file_info
   2. Define an output folder for your CSV files. In the example in Figure 3, this is given as “output_folder <- C:/htvenomics/CSVfiles”
      1. Create this output folder beforehand.
   3. Add the url of the export website your lab is using. In the example in Figure 3, this is given as “export_url <- https/ mascot.labs.vu.n/cgi/export_dat_2.pl”
      1. When using your own Mascot server there is a web page that you need to access in order to export the CSV files. In our case it is mascot.labs.vu.n/cgi/export_dat_2.pl. However, this is unique to your Mascot server but should always include: /cgi/export_dat_2.pl
   4. Add username: user = “username”
   5. Add password: password = “password”
2. Now you can run the script (Edit -> Run all) and CSV files should appear in your output folder
3. When all the CSV files for each of your searches are exported you can load the second script: Script 2_merge_export. This script merges certain information from all the CSV files into one Excel file. The information that is merged is: output name (well), species (protein identifier), protein accession, protein score, protein mass, sequence coverage, protein description, link to original Mascot search, complete protein sequence and sequences of the peptides found.


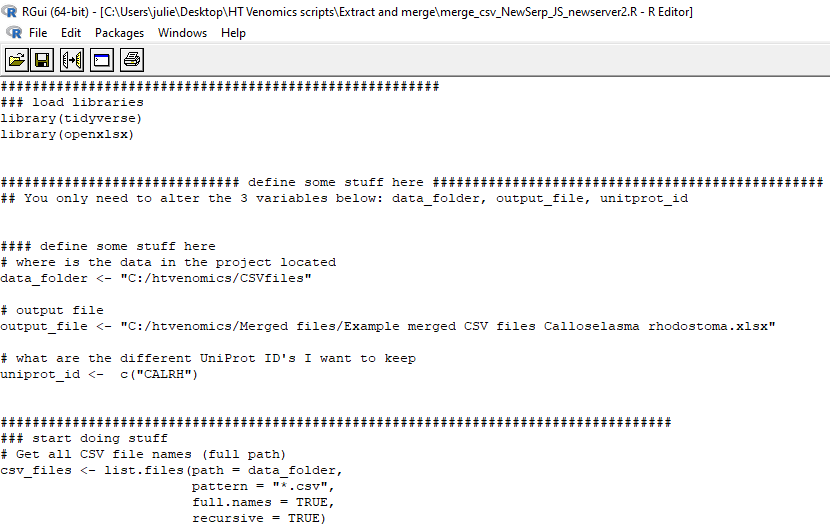


Figure 4. Script 2_merge_export.

1. Now load script 2. Part of script 2 is shown in Figure 4. Also for this script a few things have to be done:
   1. Define the folder which contains your CSV files. In the example in Figure 4, this is given as “C:/htvenomics/CSVfiles”
      1. Which you made at step 5C.
   2. Define a folder and a name for your output file which will contain all the merged data from each of your CSV files after running Script 2. In the example in Figure 4, this is given as “C:/htvenomics/merged files/Example merged CSV files Calloselasma rhodostoma.xslx”
      1. Create the folder beforehand.
      2. Output file is generated by the script into the folder created. In the example in Figure 4 this is given as “C:/htvenomics/merged files/Example merged CSV files Calloselasma rhodostoma.xslx”. In which the folder is “merged files” and the file name “Example merged CSV files Calloselasma rhodostoma.xslx”
   3. Define what protein IDs you want to filter out of your CSV files depending on the database used, sample analysed and what information you want to obtain. (You can filter out everything or just 1 parameter, depending on what you are interested in).
      1. As an example of the definition of a protein ID (from a search using Uniprot), in Figure 4 this is given: uniprot_id <- c(“CALRH”). *Note: CALRH is the Uniprot ID of Calloselasma rhodostoma. In this example, thus only results for Calloselasma rhodostoma will be used.*
   4. Run Script 2. (Edit -> Run all)
2. Now you have generated an Excel file with the merged data. An example of this data is shown in Figure 5 and is provided in the SI named: “Example merged CSV files Calloselasma rhodostoma.xlsx”.


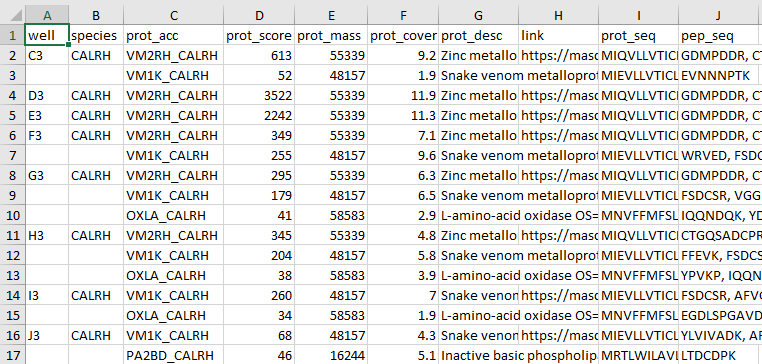


Figure 5. Example of a merged Excel file containing all specified information of the selected CSV files.

1. Now that the merged Excel file is generated, we can plot X (fractionation time) vs. Y (protein score [protein scores were used in this study, but also sequence coverage could be chosen for example if desired]) data for each of the proteins found in our analysis. This is based on the principle that proteins elute over a period of time (into different wells) and when the protein concentration increases the protein score increases too. This will result in so called protein score chromatograms (PSCs) with a highly similar shape and retention time compared to the in parallel acquired UV and Mass Spectrometry traces.
2. Now load script 3. Part of script 3 is shown in Figure 6. The important things to do:
   1. In Figure 6 you see:

“source ("C:/Users/julie/Desktop/Rico script stuff/211014_new_scripts/Protein chromatogram plotting/functionsNEW4.R") Here the location of the “functions” script, which is provided in the SI, has to be defined. It is called functionsNEW4.R.

- 1. Create a folder that you want your files to be put in. For example, “proteinchromsother”.
     1. In the script this is now shown as “proteinchromsother”.
  2. The location of the input file, which is the merged file generated with script 2, has to be defined.
     1. In the script this is shown as:

input_file <- "C:/htvenomics/proteinchromsother/Example merged CSV files Calloselasma rhodostoma.xlsx"

- 1. Define the retention time difference between fractions used for your analysis. The data sets provided in the SI have retention time frames of 0.2 min (12 sec).
  2. Set the output file name of the Total Protein Chromatogram generated (called tic_output in script 3). And its output folder.
     1. In the script this is shown as:

plot_output <- "C:/htvenomics/proteinchromsother/Example merged CSV files Calloselasma rhodostoma/Example merged CSV files Calloselasma rhodostoma.png"

tic_output <- "C:/htvenomics/proteinchromsother/Example merged CSV files Calloselasma rhodostoma/TIC_Example merged CSV files Calloselasma rhodostoma.csv"

- - 1. The Total Protein Chromatogram is a sum of all protein scores found from each of the proteins in each well.
  1. Define the location and name of the Excel files with the X Y data.
     1. In the script this is shown as:

plot_output_folder <- file.path("C:/htvenomics/proteinchromsother", "Example merged CSV files Calloselasma rhodostoma")

- 1. Define the location of the integration data.
     1. In the script this is shown as:

output_excel <- file.path("C:/htvenomics/proteinchromsother", "Example merged CSV files Calloselasma rhodostoma", "peak_detection_Example merged CSV files Calloselasma rhodostoma.xlsx")

- 1. Define the start and end retention times. Usually this starts at 0 and ends whenever the RPLC run was finished. In the SI data sets this was always 50 (i.e., 50 min).
     1. In the script shown as: rt_start <- ..

rt_end <- ..

- - 1. For our study this will then be: rt_start <- 0

rt_end <- 50

- - 1. The rest of the settings in the script can be adjusted for the more advanced user to the user’s preferences/data.
  1. Run Script 3. (Edit -> Run all)


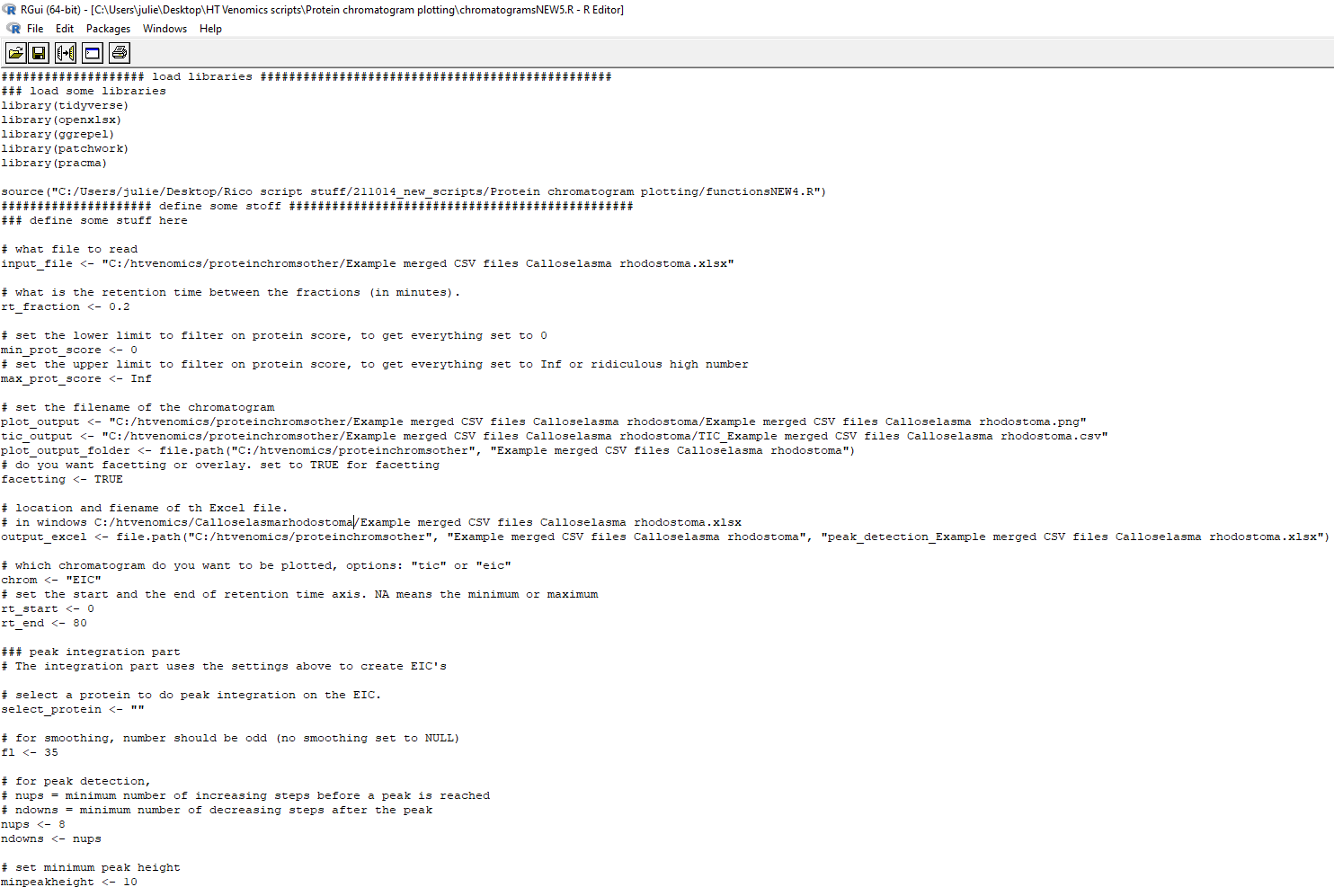


Figure 6. Script 3_Protein chromatogram plotting and Integration

1. Now all the CSV files are present in your defined folder: XY data of all the found proteins and their integration data (called peak_detection in the script). See Figure 7 for example.
   1. Note: remove the TIC file (See step 11 F) from this folder before using Script 4 since this script processes the CSV files generated with Script 3 and the TIC file needs to be excluded from this.


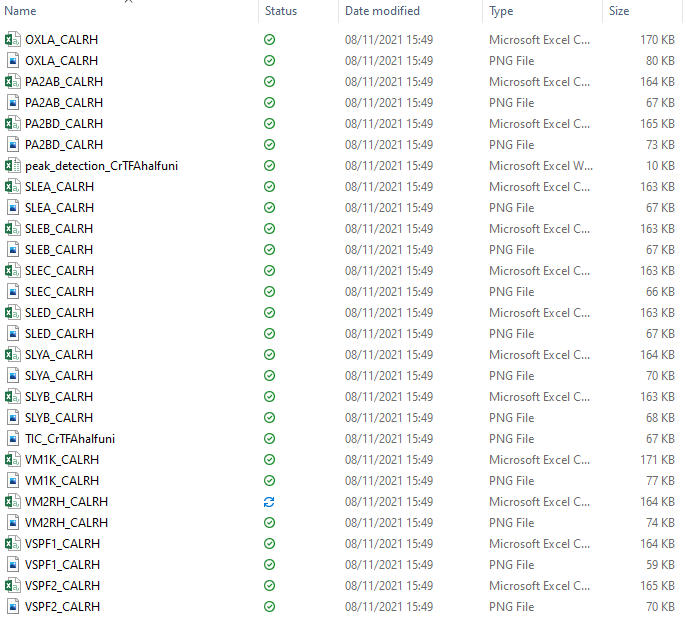


Figure 7. XY data for each of the found proteins and their integration data (peak detection).

1. Now load Script 4_ combine Y values protein chroms (see Figure 8). This script merges all XY data generated with Script 3 into a single Excel file which makes it easier to plot all this data in for example GraphPad Prism. In addition, integration of all the found proteins takes place and this data is placed in an Excel file as seen in Figure 7 as peak_detection_CrTFAhalfuni.
2. Important things in this script:
   1. Define pre-made work folder (folder which contains the folder with the XY data files generated with Script 3, in Figure 6 called “htvenomics”).
   2. Define pre-made folder which contains the XY data files generated with Script 3 (In Figure 6 called: “Example merged CSV files Calloselasma rhodostoma”).
   3. Define a pre-made export folder for the two merged files (In figure 8 called: Toxins_Yvalues). Two files will be generated. One called Y and one called YS. Y will be the raw XY data and YS will be smoothened XY data. The smoothed (YS) data looks visually more appealing when plotted but yields the same results as Y.
   4. Define the file names for the two merged files that will be generated (In Figure 8 called: “all_merged_y_Example merged CSV files Calloselasma rhodostoma.csv” and “all_merged_ys_Example merged.csv” CSV files Calloselasma rhodostoma).
   5. Define the number of fields for the protein names. For example, OXLA_CALRH will need a value of 2, since the accession consists of two parts OXLA and CALRH. If the name were to be OXLA_2_CALRH the value needs to be 3. *Note: For venom toxin IDs in Uniprot, the number is usually 2.*
   6. Run Script 4. (Edit -> Run all)


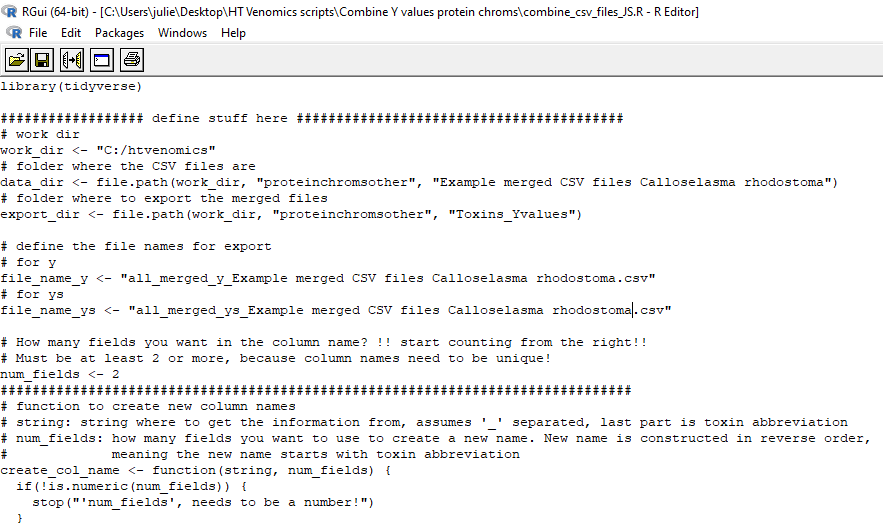


Figure 8. Script 4_ combine Y values protein chroms.

1. Now the merged XY data can be plotted in GraphPad Prism or another software package and the protein score chromatograms will be plotted as shown in Figures 9 and 10.


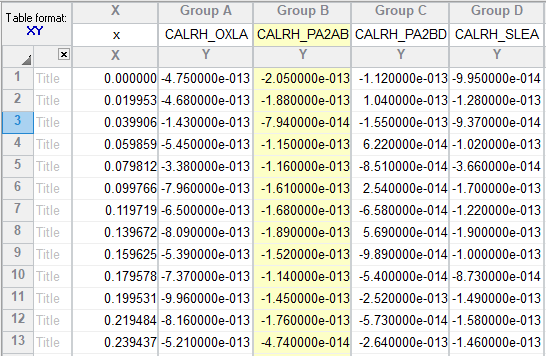


Figure 9. XY data plotted in GrapPad Prism.


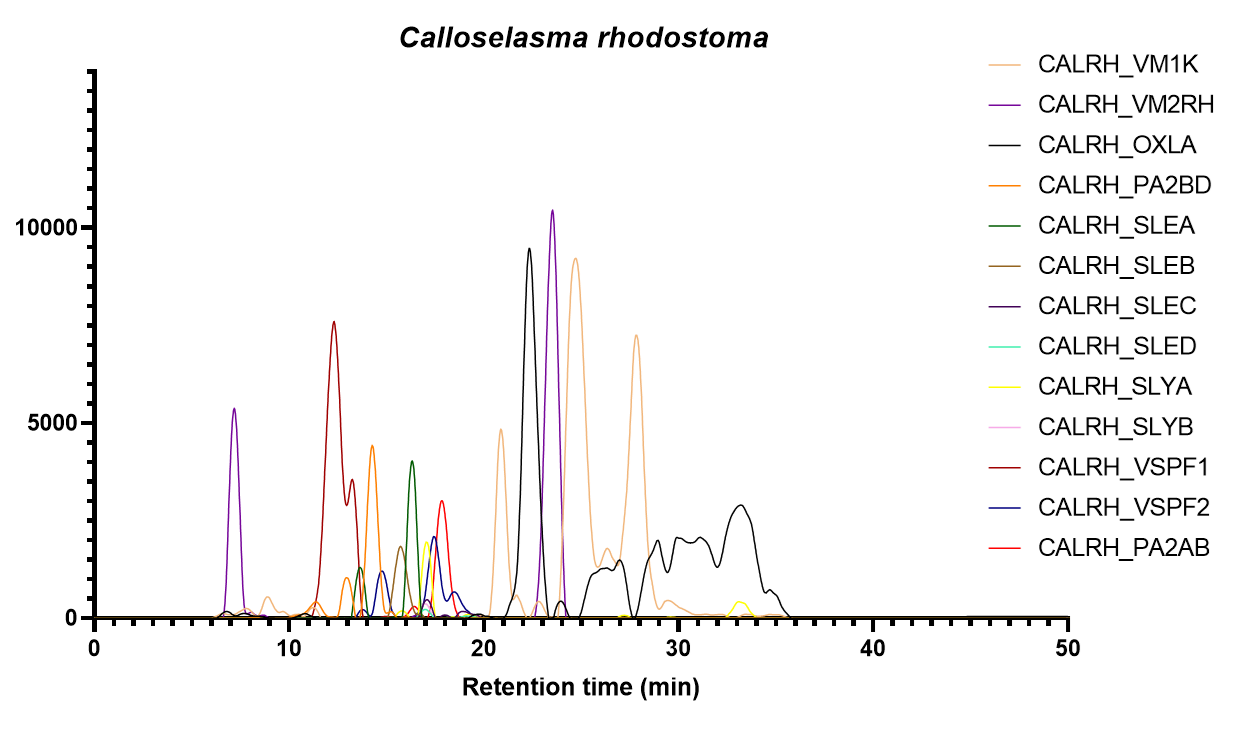


Figure 10. Protein Score Chromatograms plotted from smoothened XY data.
